# Hospital-treated infectious diseases and polygenic susceptibility in relation to heart failure and cardiac remodelling: evidence from the UK Biobank and ARIC cohorts

**DOI:** 10.1093/ehjopen/oeag036

**Published:** 2026-02-21

**Authors:** Jiazhen Zheng, Shaojun Tang, Quan Yang, Zhen Zhou, Zhuoni Zhang, S W Ricky Lee

**Affiliations:** Bioscience and Biomedical Engineering Thrust, Systems Hub, The Hong Kong University of Science and Technology (Guangzhou), No. 1 Duxue Road, Dongchong Town, Nansha District, Guangzhou, Guangdong 511453, China; Bioscience and Biomedical Engineering Thrust, Systems Hub, The Hong Kong University of Science and Technology (Guangzhou), No. 1 Duxue Road, Dongchong Town, Nansha District, Guangzhou, Guangdong 511453, China; Cardiac and Vascular Center, The University of Hong Kong-Shenzhen Hospital, No. 1 Haiyuan First Road, Futian District, Shenzhen, Guangdong 518053, China; School of Public Health and Preventive Medicine, Monash University, 553 St Kilda Rd, Melbourne, VIC 3004, Australia; Urban Governance and Design Thrust, Society Hub, The Hong Kong University of Science and Technology (Guangzhou), No. 1 Duxue Road, Dongchong Town, Nansha District, Guangzhou, Guangdong 511453, China; Bioscience and Biomedical Engineering Thrust, Systems Hub, The Hong Kong University of Science and Technology (Guangzhou), No. 1 Duxue Road, Dongchong Town, Nansha District, Guangzhou, Guangdong 511453, China

**Keywords:** Infectious disease, Heart failure, Polygenetic risk score, Cardiac remodelling

## Abstract

**Aims:**

Infectious diseases (IDs) are increasingly recognized as precipitants of heart failure (HF). However, the impact of diverse pathogens and genetic susceptibility on HF and cardiac remodelling remains unclear.

**Methods and results:**

We analysed 480 154 UK Biobank (UKB) participants and 9816 Atherosclerosis Risk in Communities (ARIC) participants free of HF at baseline. Hospital-treated IDs were ascertained with 931 ICD-10 codes and modelled as time-varying exposures. Incident HF and HF with preserved (HFpEF) and reduced ejection fraction (HFrEF) were identified through hospital records and adjudication. Polygenic risk scores (PRSs) for ID—overall and pathway-specific for TGF-β, acute inflammation, and myocardial fibrosis—were derived from genome-wide association study data. Multivariable Cox and Fine–Gray models estimated hazard ratios (HRs); cardiac structure and function were assessed by cardiac MRI in UKB and echocardiography in ARIC. Over median follow-up of 13.5 years (UKB) and 22.4 years (ARIC), any hospital-treated ID was associated with higher HF risk (UKB HR 1.54, 95% CI 1.46–1.63; ARIC HR 1.84, 1.68–2.00). Risks were similar for bacterial, viral, fungal, and parasitic infections, peaked within 180 days (HR 5.88), and persisted >1 year. Infectious diseases increased both HFpEF (sub-HR 1.81) and HFrEF (2.03). Imaging revealed higher left-ventricular mass, wall thickness, mass-to-volume ratio and filling pressures, and lower ejection fraction among infected individuals. In infection-free participants, higher overall ID-PRS predicted incident HF (per-SD HR 1.07 UKB; 1.11 ARIC) and adverse cardiac remodelling, with strongest effects for TGF-β and acute-inflammation pathways.

**Conclusion:**

Hospital-treated IDs and genetic predisposition to infection are independently associated with HF and adverse cardiac phenotypes. Integrating infection history and ID-PRS may enhance HF risk stratification and motivate trials targeting fibro-inflammatory pathways.

## Graphical Abstract

**Graphical Abstract:**
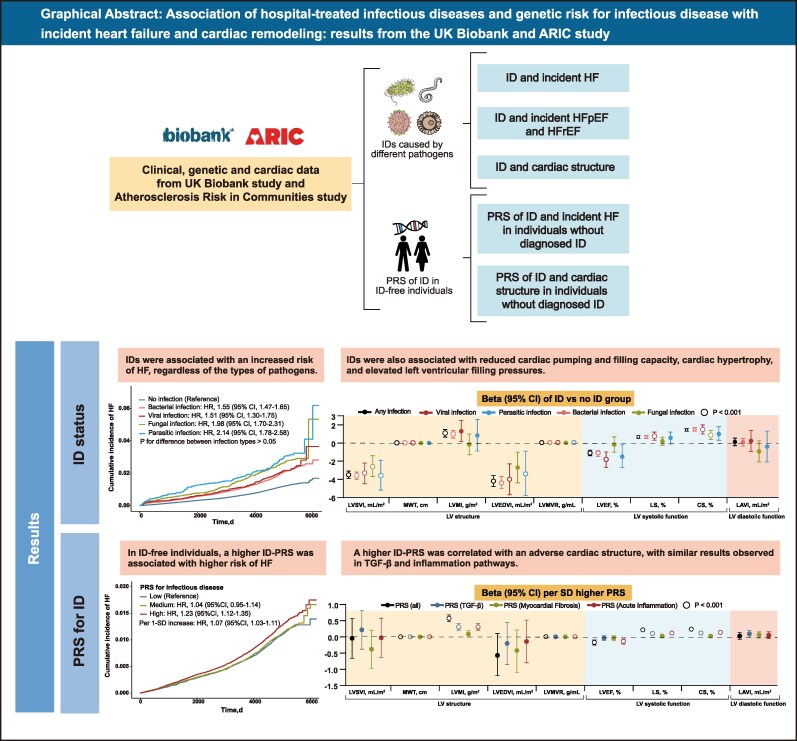

## Introduction

Infectious diseases (IDs) continue to be a major global health concern, driving substantial morbidity and mortality. In 2019, IDs contributed to around 704 million disability-adjusted life years, representing 27.7% of the global disease burden.^1^ Increasing evidence suggests that infection plays a crucial role in the pathogenesis and progression of different heart failure (HF) phenotypes.^2^ Reflecting this, the 2021 guidelines from the European Society of Cardiology updated the CHAMP acronym to CHAMPIT, incorporating infection (I) and tamponade (T) as conditions that needed to be treated urgently.^3^ Though ID is recognized as a factor that worsens HF, whether it serves as a risk factor for increasing subsequent HF risk and whether the association between ID and HF is limited to specific microbe remain poorly understood.

Previous investigations have explored the association between specific ID and HF, though the scope and findings have varied. Eurich *et al*.^4^ found that community-acquired pneumonia substantially increases the risk of HF by 61%. In a self-controlled case series, Sin *et al*.^5^ reported that the hospitalization rate for HF is 33 times higher in the first week following laboratory-confirmed influenza infection compared to the control period. Chronic hepatitis C virus infection was reported to increase the risk of HF.^6,7^ In a self-controlled case-series design, the risk of HF was approximately nine times greater for patients hospitalized with dengue infection compared to those not admitted.^8^ However, these studies were mostly limited by their cross-sectional design, smaller sample sizes, and shorter follow-up durations and often focused on single or select types of infections. Additionally, they did not differentiate between HF subtypes [i.e. HF with preserved ejection fraction (HFpEF) and HF with reduced ejection fraction (HFrEF)].

Structural abnormalities in the heart have been linked to adverse cardiovascular outcomes, including HF. Certain pathogens, like *Francisella tularensis* in sepsis, can cause myocardial damage with electrical and structural changes, such as prolonged QRS intervals and left ventricular dysfunction.^9^ Viral infections, like Coxsackie B virus, also result in myocardial damage, including necrosis and viral crystal formation.^10^ However, some studies show conflicting results, suggesting that certain viral infections, like EBV and HHV-6, can cause structural damage to the heart, while others may not affect heart architecture.^11^ Prior research has been constrained by focusing on specific infections and limited cardiac phenotypes, often with small sample sizes.

Recent developments in large-scale genetic studies and biobanks have facilitated the discovery of numerous genetic variants associated with complex diseases. Polygenic risk scores (PRSs) are numerical values reflecting an individual’s genetic predisposition to a disease based on the cumulative effects of numerous genetic variants and have emerged as useful markers for ID.^12^ Cardiovascular health was reported to be affected by PRS for other conditions like diabetes and schizophrenia.^13,14^ This study aims to extend previous findings by exploring the association between PRS for ID and HF in individuals without ID and also investigates the fibro-inflammatory gene pathway PRS, due to their relevance to ID, HF, and cardiac fibrosis.^15^ This analysis aims to improve risk classification in non-infected individuals and foster early HF prevention strategies in genetically predisposed individuals.

In this study, we utilized data from the Atherosclerosis Risk in Communities (ARIC) study and the UK Biobank (UKB) cohort to examine the association between a broad spectrum of ID and the incidence of HF, along with parameters of cardiac morphology and function. Within the ARIC cohort, we further assessed the risk associated with HFpEF and HFrEF. Additionally, we investigated the association of overall PRS and fibro-inflammatory pathway-specific PRS for ID with incident HF and cardiac phenotypes in participants without overt infection.

## Methods

### Study population

Participants from the UKB, a cohort study involving more than 500 000 UK residents since 2006, were included in this research. The study’s goals, participant demographics, and data collection methods have been previously documented.^16^ Briefly, data collection involved questionnaires, interviews, regular assessment centre visits, and health record linkages, covering diverse psychosocial, sociodemographic, physical, and genetic variables. Ethical approval was obtained from the North West Multicenter Research Ethics Committee, with informed consent given by all participants at the first visit. The UKB application number for this study is 97089.

The ARIC study enrolled 15 792 participants from four US communities, with the initial visit occurring between 1987 and 1989.^17^ Participants attended follow-up visits and received annual phone calls, with the frequency increasing to semi-annual calls starting in 2012. The baseline for this analysis is Visit 4, conducted between 1996 and 1998, with 11 656 participants in attendance.

**Figure 1:**
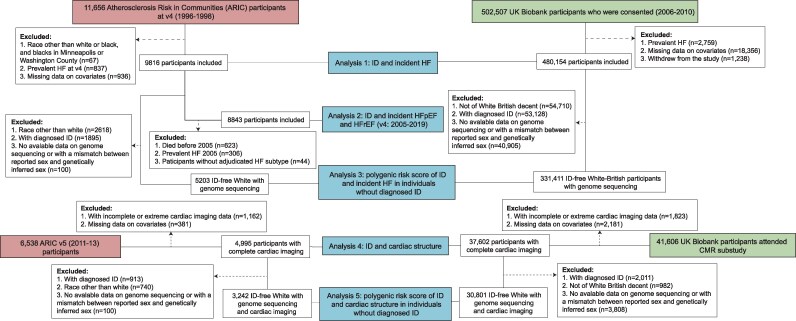
Flow chart of the study. Analysis 2 was only performed in ARIC due to HF classification being unavailable in UKB. ID, infectious disease; HFpEF, heart failure with preserved ejection fraction; HFrEF, heart failure with reduced ejection fraction; HF, heart failure.

For the Analysis 1, the primary analysis in the current study, participants were excluded if they had a history of HF or missing data on covariates, were Black in Minnesota and Washington County or individuals from racial groups other than Black or White (for ARIC), or withdrew from the study. Ultimately, in Analysis 1, ARIC included 9816 participants, while UKB included 480 154 participants. The flow chart for Analyses 1–5 is shown in *Figure 1*.

### Ascertainment of infectious diseases

Individuals in the UKB were connected to national health registries. In ARIC, hospitalizations were confirmed through telephone calls and local hospital record. We examined hospital-treated IDs as the main exposure, utilizing 931 International Classification of Diseases, 10th Revision (ICD-10) codes to identify IDs that were documented as the primary cause for hospital admissions. Infectious diseases were classified into bacterial, viral, fungal, and parasitic infections based on the type of pathogen. Our categorization for ID type is based on an earlier study.^18^  Supplementary material online, *Table S10* provides the ICD codes for these disease categories. In this study, ID was analysed as a time-varying exposure, with ID and outcome dates recorded for exposed participants. Individuals were categorized as exposed if they had an ID before or during the study, while those without ID remained unexposed. Participants who developed an ID during follow-up contributed to the unexposed group until the ID diagnosis and then shifted to the exposed group. To evaluate the short- and long-term effects of IDs on HF, HRs were calculated for events within the first 180 days, between Days 181 and 365, and after 1 year (Day 366+).

### Incident heart failure assessment

The primary outcome was incident HF, defined as the first HF-related hospitalization or death occurring after instance 0 in UKB or Visit 4 in ARIC, with follow-up ending on 1 July 2023 for UKB and 31 December 2019 for ARIC. In Analysis 1, hospitalizations caused by incident HF were ascertained using the ICD-10 code I50. Those diagnosed before the baseline time were considered to have prevalent HF. From 2005, ARIC implemented additional physician adjudication for HF events, facilitating the assessment of HF subtypes.^19^ In Analysis 2, HFpEF was identified by a left ventricular ejection fraction of ≥50% at the time of the first HF hospitalization, whereas HFrEF was defined as a left ventricular ejection fraction <50% at incident HF hospitalization.

### Derivation of pathway-based polygenic risk score for infectious disease

We constructed overall and pathway-based PRSs for IDs using summary statistics from a previous genome-wide association study (GWAS) and PRSice-2,^12^ following standard quality-control and linkage disequilibrium–pruning procedures (details in Supplementary material online, *eMethods*). Pathway-based PRSs focused on transforming growth factor-beta (TGF-β) signalling, myocardial fibrosis, and acute inflammation, using gene sets from the Molecular Signatures Database.^20^

### Cardiac magnetic resonance imaging protocol and echocardiography assessment

The UKB Imaging Study, launched in 2018 (average 9.7 years after the original baseline assessment), is an ongoing study that aims to collect detailed imaging data from up to 100 000 participants from the original UKB cohort. The UKB cardiovascular magnetic resonance (CMR) protocol was described previously.^21^ Cardiac morphology in ARIC was examined during Visit 5 (2011–13) (average 14.8 years after the Visit 4 baseline) by certified sonographers, using a comprehensive echocardiographic protocol that has been documented earlier.^22^ Detailed information on cardiac metrics collection are described in Supplementary material online, *eMethods*. Cardiac phenotypes were selected based on previous UKB and ARIC imaging studies, with a preference for variables that were present in both studies. Where necessary, these phenotypes were adjusted for body surface area. Cardiac metrics in this study included left ventricular stroke volume index (LVSVI) (UKB only), mean wall thickness (MWT), left ventricular mass index (LVMI), left ventricular end-diastolic volume index (LVEDVI), left ventricular mass-to-volume ratio (LVMVR), left ventricular ejection fraction (LVEF), longitudinal strain (LS), circumferential strain (CS), left atrial volume index (LAVI), Septal e′ (ARIC only), and E/e′ ratio (ARIC only). Left ventricular mass-to-volume ratio is calculated by dividing the LVM by the LVEDV.

### Ascertainment of covariates

In the UKB and ARIC study, covariates were determined by structured surveys, clinical assessments, and lab analyses. Sociodemographic factors included age, sex, ethnicity, Townsend Deprivation Index (TDI), household income, and level of education. Health related index included height, weight, systolic blood pressure, diastolic blood pressure, HbA1c, LDL-C, triglycerides, and estimated glomerular filtration rate (eGFR). Personal medical condition included diabetes, hypertension, chronic kidney disease (CKD), atrial fibrillation (AF), and coronary heart disease (CHD).

Area-level socio-economic status was assessed using the TDI, a composite score derived from national census data on unemployment, non-car ownership, non-home ownership, and household overcrowding in the participant’s residential postcode area, with higher values indicating greater deprivation. Trained nurses measured blood pressure, with mean blood pressure calculated from either two automated or two manual readings. Estimated glomerular filtration rate was calculated based on the CKD-EPI equation.^23^ Measurements of HbA1c, LDL-C, and triglycerides have been described previously. An eGFR below 60 mL/min/1.73 m² indicated CKD. Hypertension was defined by either antihypertensive medication uses or blood pressure readings of 140/90 mmHg or higher. Diabetes mellitus criteria included self-reported diagnosis, glucose-lowering medication use, a fasting glucose level of at least 126 mg/dL, or a non-fasting glucose level of at least 200 mg/dL. In the UKB, AF and CHD were identified using a combination of self-reports and diagnosis records from linked databases. In ARIC, AF was detected through study electrocardiograms and ICD codes from hospitalization surveillance data. Prevalent CHD was defined by self-reported CHD or confirmed cases of fatal or nonfatal myocardial infarction, adjudicated by two physicians based on cardiac symptoms, electrocardiogram results, and cardiac enzyme levels or evidence of prior myocardial infarction on electrocardiograms.

### Statistical analysis

Continuous variables were reported as mean ± standard deviation (SD), while categorical variables were shown as number (percentage). *P* values were determined using the χ² test for categorical variables and the Mann–Whitney *U* test for continuous variables to assess differences across ID categories. We used Kaplan–Meier analysis with the log-rank test to compare HF incidence across different ID categories. To assess the association between baseline ID, ID-PRS, and incident HF risk, we employed multivariable Cox proportional hazards regression, calculating hazard ratios (HRs) and 95% confidence intervals (CIs). The Fine–Gray sub-distribution hazards model was also used to examine baseline ID’s impact on HF subtypes (HFpEF and HFrEF), considering competing risks, including death, alternative HF subtypes, and unclassified HF due to unavailable ejection fraction data.^24^ Multivariable linear regression was used to explore the relationship of the ID and ID-PRS (by 1-SD increase) and cardiac metrics, reporting standardized beta coefficients, 95% CIs, and *P* values. We used two multivariable models to account for potential confounders. The first model (Model 1) adjusted for sex and age (time scale). Model 2 further controlled for ethnicity, level of education, smoking status, alcohol drinking, height, weight, hypertension, diabetes, CKD, and LDL cholesterol.

Subgroup analysis was performed to assess possible modification effects of the following variables: sex (female or male), age (<60 or ≥60 in UKB; <65 or ≥65 in ARIC), ethnicity (White or non-White), smoking status (never or ever), obesity (BMI ≥30 kg/m^2^ or not), diabetes (absent or present), hypertension (absent or present), AF (absent or present), and CHD (absent or present). In Model 2, we assessed possible interactions by adding product terms between the exposure and the key stratified factors.

To validate our results, we performed several sensitivity analyses, which included (i) performing a competing risk analysis with non-HF death as a competing event for HF, (ii) adding adjustments for baseline CHD and AF, (iii) excluding patients infected with more than one type of pathogen (e.g. individuals infected simultaneously by bacterial and viral pathogens), and (iv) excluding individuals without any hospitalization records.

All statistical analyses were performed using R version 4.0.3, with a two-sided significance threshold of *P* < 0.05. To account for multiple comparisons, the Bonferroni correction was applied, adjusting the significance level to 0.001 (0.05/45).

## Results

### Population characteristics

*Table 1* shows baseline characteristics of participants from UKB and ARIC. Among 480 154 participants from UKB (mean age 56.3 years, 55.9% women), 90 258 (18.8%) were hospitalized for ID, in which 72659 were classified as bacterial infection, 8547 were viral infection, 6626 were fungal infection, and 4285 were parasitic infection. Among 9816 participants from ARIC (mean age 63.0 years, 58.5% women), 3854 (39.3%) were hospitalized for ID, in which 2876 were classified as bacterial infection, 485 were viral infection, 398 were fungal infection, and 418 were parasitic infection (see Supplementary material online, *Table S1*). Adults hospitalized for ID tended to be older, male (UKB), non-White (UKB), more educated (UKB), current smokers (UKB), and never drinkers (UKB) and had a higher prevalence of comorbidities.

**Table 1:** Baseline characteristics of participants in the UK Biobank (2006–10) and ARIC study (Visit 4) by infection status

	UKB				ARIC			
	Overall	No ID	ID	*P* value	Overall	No ID	ID	*P* value
No. of participants	480 154	389 896	90 258		9816	5962	3854	
Age	56.3 (8.1)	55.8 (8.1)	58.1 (7.9)	<0.001	63.0 (5.5)	62.8 (5.5)	63.4 (5.7)	<0.001
Women	268 374 (55.9)	218 732 (56.1)	49 642 (55.0)	<0.001	5739 (58.5)	3446 (57.8)	2293 (59.5)	0.098
White	451 164 (94.0)	366 502 (94.0)	84 662 (93.8)	0.023	7198 (73.3)	4334 (72.7)	2864 (74.3)	0.081
More than high school	225 366 (46.9)	185 201 (47.5)	40 165 (44.5)	<0.001	3189 (32.5)	1979 (33.2)	1210 (31.4)	0.066
Townsend Deprivation Index	−1.3 (3.1)	−1.4 (3.0)	−1.0 (3.2)	<0.001	–	–	–	–
House hold income < 18 000£	113 316 (23.6)	92 132 (23.6)	21 184 (23.5)	0.148	–	–	–	–
Current smoker	49 944 (10.4)	38 210 (9.8)	11 734 (13.0)	<0.001	1513 (15.4)	912 (15.3)	601 (15.6)	0.712
Never drinker	147 083 (30.6)	117 749 (30.2)	29 334 (32.5)	<0.001	1986 (20.2)	1169 (19.6)	817 (21.2)	0.059
Height, cm	169.7 (9.8)	169.6 (10.0)	169.8 (10.2)	<0.001	168.8 (9.5)	168.9 (9.6)	168.6 (9.5)	0.011
Weight, kg	75.8 (15.3)	75.7 (15.3)	76.1 (15.5)	<0.001	77.9 (16.6)	78.0 (16.7)	77.8 (16.5)	0.587
Diabetes	24 759 (5.2)	19 885 (5.1)	4874 (5.4)	<0.001	1291 (13.2)	763 (12.8)	528 (13.7)	0.207
Hypertension	270 782 (56.4)	218 342 (56.0)	52 440 (58.1)	<0.001	4151 (42.3)	2432 (40.8)	1719 (44.6)	<0.001
CKD	6333 (1.3)	5069 (1.3)	1264 (1.4)	0.018	180 (1.8)	95 (1.6)	85 (2.2)	0.033
Atrial fibrillation	4592 (1.0)	3509 (0.9)	1083 (1.2)	<0.001	308 (3.1)	173 (2.9)	135 (3.5)	0.107
Coronary heart disease	17 737 (3.7)	14 036 (3.6)	3701 (4.1)	<0.001	828 (8.4)	489 (8.2)	339 (8.8)	0.318
Systolic blood pressure, mmHg	138.3 (18.1)	138.2 (18.3)	138.7 (18.7)	<0.001	125.8 (18.5)	125.5 (18.4)	126.3 (19.2)	<0.001
Diastolic blood pressure, mmHg	82.4 (10.1)	82.3 (10.1)	82.8 (10.3)	<0.001	71.2 (10.2)	70.9 (10.1)	71.6 (10.2)	0.018
HbA1c, %	5.5 (0.6)	5.5 (0.6)	5.6 (0.7)	0.237	5.7 (0.5)	5.7 (0.5)	5.8 (0.7)	<0.001
LDL-C, mmol/L	3.55 (1.7)	3.54 (1.8)	3.58 (2.1)	<0.001	3.16 (0.9)	3.12 (0.9)	3.23 (0.9)	<0.001
Triglycerides, mmol/L	1.48 (0.8)	1.48 (0.9)	1.49 (1.1)	0.540	1.51 (0.8)	1.51 (0.8)	1.52 (0.8)	0.225
eGFR, mL/min/1.73 m^2^	95.6 (13.8)	95.7 (14.0)	95.3 (14.6)	<0.001	86.7 (14.1)	87.0 (14.1)	86.2 (14.3)	<0.001

Data are presented as no. (%) or mean (SD).

ID, infectious disease; CKD, chronic kidney disease; CHD, coronary heart disease; HbA1c, haemoglobin A1c; LDL-C, LDL cholesterol; eGFR, estimated glomerular filtration rate.

*P values were obtained from either a χ² test or a Mann–Whitney *U* test comparing difference between the ID and No ID group.

### Infectious disease treated in the hospital and incident heart failure

**Figure 2:**
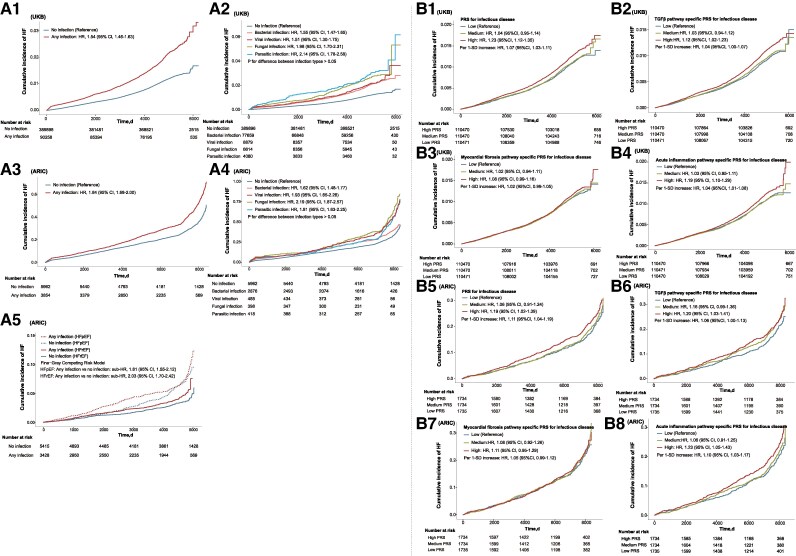
Association between (*A*) infectious diseases and (*B*) pathway-based polygenetic risk score for infectious disease and heart failure in the UK Biobank and ARIC study. Cox proportional hazards models were adjusted for sex, age (time scale), ethnicity (Section A only), level of education, smoking status, alcohol drinking, height, weight, hypertension, diabetes, chronic kidney disease, and LDL cholesterol. A2 and A4 classified infectious diseases based on ICD-10 codes, and differences in the impact of various infections were assessed by comparing the value of events/person-years. A5 used Fine−Gray Competing Risk Model, accounting for the competing risks of other subtypes of heart failure. In Section B, genetic pathways associated with TGF-β signalling, myocardial fibrosis, and acute inflammation were selected from the Molecular Signatures Database (version 7.4) to calculate the pathway-based polygenic risk score. The polygenic risk score was then divided into tertiles, with the lowest group serving as the control. Only individuals without a history of diagnosed infectious diseases were included in the analysis for the polygenic risk score. HR, hazard ratio; sub-HR, subfraction HR; HF, heart failure; HFpEF, heart failure with preserved ejection fraction; HFrEF, heart failure with reduced ejection fraction.

In UKB, over a median 13.5-year follow-up, we documented 6484 incident HF. In ARIC, 2062 incident HF (a median follow-up of 22.4 years) were recorded. Survival curves revealed a higher risk of incident HF in participants with ID treated in the hospital compared with the individuals without ID treated in the hospital (*Figure 1*). After controlling for multiple variables in COX model, the HRs associated with any ID treated in the hospital, bacterial infection, viral infection, fungal infection, and parasitic infection for incident HF in the UKB are 1.54 (95% CI 1.46–1.63), 1.55 (95% CI 1.47–1.65), 1.51 (95% CI 1.30–1.75), 1.98 (95% CI 1.70–2.31), and 2.14 (95% CI 1.78–2.58), respectively (*Figure 2A1* and *A2*; Supplementary material online, *Table S2*). In the ARIC study, these HRs are 1.84 (95% CI 1.68–2.00), 1.62 (95% CI 1.48–1.77), 1.93 (95% CI 1.65–2.26), 2.19 (95% CI 1.87–2.57), and 1.91 (95% CI 1.63–2.25), respectively (*Figure 2A3* and *A4*; Supplementary material online, *Table S2*). The *P* value for the difference in HF incidence among individuals with different infection types was greater than 0.05 in both UKB and ARIC study. In the ARIC study, over a median follow-up period of 14.1 years, 628 cases of HFpEF and 506 cases of HFrEF were identified. Using a Fine−Gray Competing Risk Model, ID treated in the hospital was link to a higher risk for both HFpEF (sub-HR 1.81, 95% CI, 1.55–2.12) and HFrEF (2.03, 95% CI, 1.70–2.42) (*Figure 2A5*).

**Figure 3:**
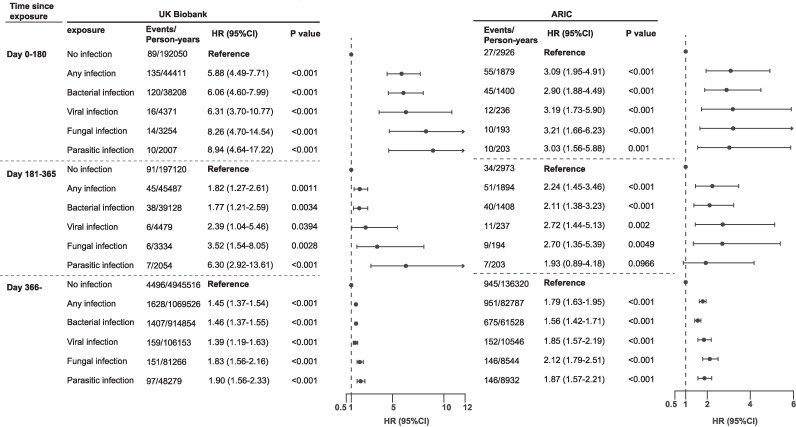
Risk of heart failure associated with any infectious disease and bacterial, viral, fungal, and parasitic infections by time since infection in the UK Biobank and ARIC study. Cox proportional hazards models were adjusted for sex, age (time scale), ethnicity, level of education, smoking status, alcohol drinking, height, weight, hypertension, diabetes, chronic kidney disease, and LDL cholesterol. HR, hazard ratio.

In the UKB, any infection within 0–180 days was associated with a substantially higher HF risk (HR 5.88, 95% CI 4.49–7.71) (*Figure 3*). Bacterial, viral, fungal, and parasitic infections were all significantly associated with increased risk during this period, with fungal (HR 8.26, 95% CI 4.70–14.54) and parasitic infections (HR 8.94, 95% CI 4.84–17.22) posing the highest risks. A similar pattern was observed in ARIC, with viral infection showing the highest risk (HR 3.19, 95% CI 1.73–5.90). At 181–365 days, infection-related HF risk persisted, although at lower levels in both cohorts. The risk persisted but diminished after 366 days across all infection types.

**Figure 4:**
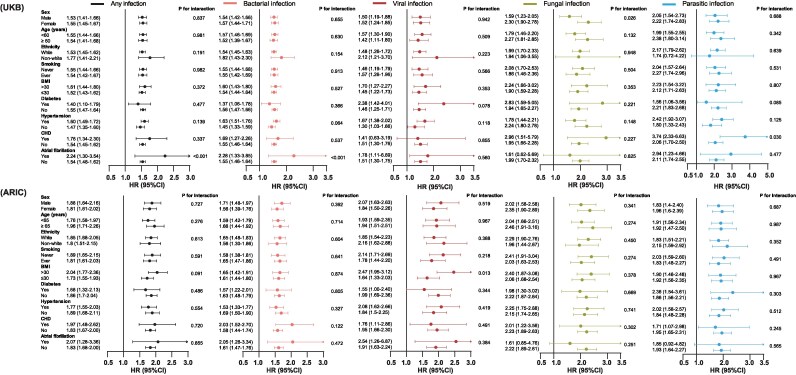
Subgroup analyses of infectious diseases and risk of heart failure in the UK Biobank and ARIC study. Cox proportional hazards models were adjusted for sex, age (time scale), ethnicity, level of education, smoking status, alcohol drinking, height, weight, hypertension, diabetes, chronic kidney disease, and LDL cholesterol. HR, hazard ratio; BMI, body mass index; CHD, coronary heart disease.

*Figure 4* presents HRs for HF across different infection types (any, bacterial, viral, fungal, and parasitic) in the UKB and ARIC cohorts, stratified by various risk factors. In UKB, individuals having AF showed a substantially higher HF risk associated with any infection (HR 2.24, 95% CI 1.30–3.54) and bacterial infection (HR 2.28, 95% CI 1.33–3.85), with the interaction *P* value being less than 0.001. After applying the Bonferroni correction (with significance set at *P* < 0.001), the other stratified variables did not show a significant impact on the link between ID and incident HF (*P* for interaction > 0.001).

Consistent results were obtained across several sensitivity analyses conducted separately, including the use of Fine–Gray models for competing risks, additional adjustments for CHD and AF, further adjustments for TDI and annual household income, the exclusion of patients infected with more than one pathogen, and the exclusion of individuals without any hospitalization records (see Supplementary material online, *Table S3*).

### Pathway-based polygenic risk score for infectious disease and incident heart failure

Baseline characteristics according to tertiles of pathway-based PRS for ID in the UKB and ARIC study are shown in Supplementary material online, *Table S4*. Distribution density plots of pathway-based PRS for ID in UKB and ARIC study across different stages of analysis are presented in Supplementary material online, *Figure S1*. Across both cohorts, individuals with higher PRS for ID generally showed an increased incidence of HF compared to those with lower PRS (*Figure 2*). In the UKB, compared with the low PRS reference group, high PRS for ID was associated with HF, with an HR of 1.23 (95% CI, 1.12–1.35) and a per 1-SD increase HR of 1.07 (95% CI, 1.03–1.11). High PRS for the TGF-β pathway was associated with an HR of 1.12 (95% CI, 1.02–1.23), with a per 1-SD increase HR of 1.04 (95% CI, 1.00–1.07) (see Supplementary material online, *Table S5*). The acute inflammation pathway showed an HR of 1.19 (95% CI, 1.10–1.29), with a per 1-SD increase HR of 1.04 (95% CI, 1.01–1.08). Similarly, in the ARIC cohort, the overall PRS, TGF-β pathway, and acute inflammation pathway-specific PRS also showed significant associations, with per 1-SD increases showing HRs of 1.11, 1.06, and 1.10, respectively (see Supplementary material online, *Table S5*).

No significant interaction effect was observed between overall PRS, specific PRS (TGF-β pathway, myocardial fibrosis pathway, and acute inflammation pathway), and potential risk factors on the risks of incident HF (*P* > 0.05) (see Supplementary material online, *Figure S2*).

### Infectious disease treated in the hospital and cardiac structure and function

**Figure 5:**
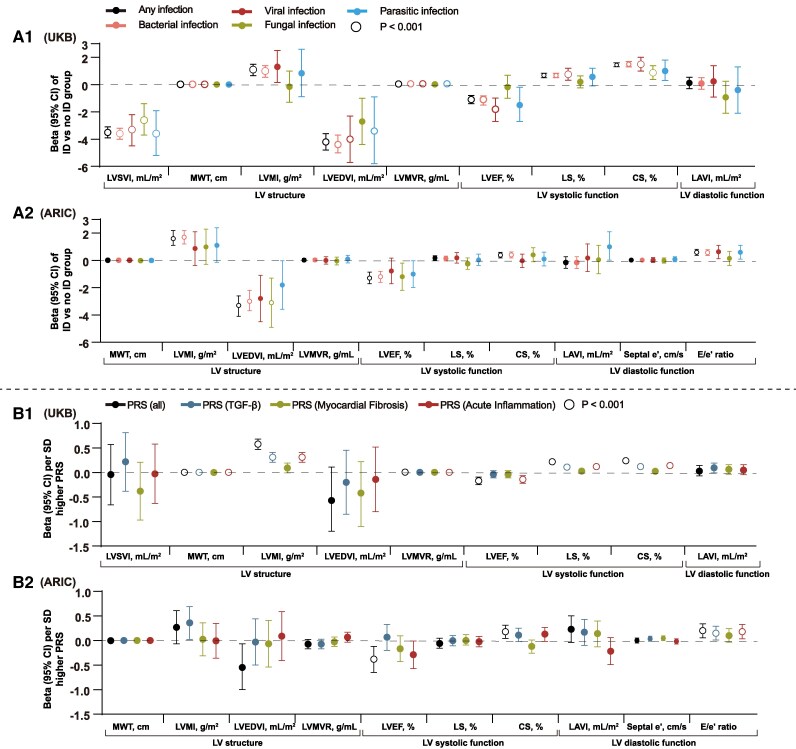
Multivariable regression results for the association between (*A*) infectious diseases and (*B*) pathway-based polygenetic risk score for infectious disease and cardiovascular measurements in the UK Biobank (CMR sub-study) and ARIC study (Visit 5). Model was adjusted for sex, age, ethnicity (Section A only), level of education, smoking status, alcohol drinking, height, weight, hypertension, diabetes, chronic kidney disease, and LDL cholesterol. Only individuals without a history of diagnosed infectious diseases were included in the analysis for the polygenic risk score. LVSVI, left ventricular stroke volume index; MWT, mean wall thickness; LVMI, left ventricular mass index; LVEDVI, left ventricular end-diastolic volume index; LVMVR, left ventricular mass-to-volume ratio; LVEF, left ventricular ejection fraction; CS, circumferential strain; LS, longitudinal strain; LAVI, left atrial volume index.

Participants in the UKB CMR sub-study were younger and predominantly White and had a relatively low prevalence of diabetes and atrial fibrillation, whereas ARIC imaging participants were older and carried a higher burden of cardiometabolic comorbidities (see Supplementary material online, *Table S6*). In the UKB, although there was some variability across specific infection types, infections were generally associated with significant reductions in LVSVI, LVEDVI, and LVEF, along with increases in MWT, LVMI, LVMVR, LS, and CS (*Figure 5*; Supplementary material online, *Table S7*). For example, any infection and bacterial, viral, and parasitic infections were linked to reductions in LVSVI of 3.5 mL/m², 3.6 mL/m², 3.3 mL/m², 2.6 mL/m², and 3.6 mL/m² and increases in CS of 1.4%, 1.5%, 1.5%, 0.88%, and 1.0%, respectively. The ARIC data generally mirrored these findings, though with slightly less pronounced coefficients. Specifically, ARIC measures of LVMI, LVEDVI, LVEF, CS, and the E/e′ ratio exhibited notable changes in individuals with any infection. The E/e′ ratio increased by 0.59, 0.57, 0.63, and 0.60 in any infection and in bacterial, viral, and parasitic infections, respectively.

### Pathway-based polygenic risk score for infectious disease and cardiac structure and function

Participants in Analysis 5 differed across cohorts, with the UKB CMR sub-study including younger, predominantly White individuals, and ARIC including older participants with more cardiometabolic risk factors (see Supplementary material online, *Table S8*). In the UKB, a 1-SD increase in overall PRS was associated with increases in MWT (0.0051 cm, *P* < 0.001), LVMI (0.58 g/m², *P* < 0.001), LVMVR (0.0082 g/mL, *P* < 0.001), LS (0.22%, *P* < 0.001), and CS (0.24%, *P* < 0.001), along with a reduction in LVEF of 0.17% (*Figure 5*; Supplementary material online, *Table S9*). Polygenic risk score related to the TGF-β pathway and acute inflammation pathway showed a similar trend in changes to cardiac structure as seen with overall PRS. The ARIC cohort findings generally mirrored those in the UKB, though the changes were less pronounced. In ARIC, a 1-SD increase in overall PRS was linked to a 0.0055 mL/m² increase in CS (*P* < 0.001), a 0.2% increase in the E/e’ ratio (*P* = 0.006), a 0.55 mL/m² decrease in LVEDVI (*P* = 0.026), and a 0.38% decrease in LVEF (*P* = 0.005). The impacts on cardiac measurements were generally consistent in PRS related to the TGF-β pathway and the acute inflammation pathway.

## Discussion

In two nationwide US and UK cohorts, we explored the associations between a full spectrum of hospital-confirmed ID and HF risk. After adjusting for a wide range of potential confounders, hospital-treated ID was associated with a 1.5-fold increased risk of HF over a 13.5-year follow-up in approximately 480 000 participants in the UKB and a 1.8-fold increased risk over a 22.4-year follow-up in about 9800 participants in the ARIC study. In ARIC, similar results were observed for both incident HFpEF and HFrEF. These associations were consistent across bacterial, viral, fungal, and parasitic infections. Although the risk decreased over time, it remained elevated long term. A significant interaction was found between ID and AF on HF risk. In cardiac imaging analyses, IDs were associated with adverse cardiac remodelling. We extended these findings by showing that higher PRS for ID in ID-free individuals was also associated with a higher incidence of HF and adverse cardiac phenotype, particularly through the TGF-β and acute inflammatory pathways.

To the best of our knowledge, this study is among the first to comprehensively evaluate the association between a wide range of IDs and incident HF. Earlier evidence regarding specific infections supports our findings. A population-based clinical registry by Eurich *et al*.^4^ found that community-acquired pneumonia increases the risk of HF by 61%. This is consistent with our results, which show an HR of 1.54 (95% CI 1.46–1.63) for any ID. The associations showed little specificity by infection type, including bacterial, viral, fungal, and parasitic infections. We complement existing research on the associations between a broad spectrum of pathogens and HF risk.

Although HFrEF and HFpEF differ in their predominant pathophysiological mechanisms, severe IDs may activate a set of shared biological pathways that plausibly increase the risk of both phenotypes. Systemic and persistent inflammation, endothelial dysfunction, microvascular impairment, neurohormonal activation, and fibro-inflammatory myocardial remodelling may all contribute to adverse cardiac structural and functional changes after infection, potentially manifesting as either reduced or preserved ejection fraction HF depending on the underlying myocardial substrate and comorbidity profile. In our study, the associations between hospital-treated IDs and incident HFrEF and HFpEF were of broadly similar magnitude with overlapping confidence intervals. However, this should not be taken as evidence that the underlying mechanisms are identical; HF phenotypes remain heterogeneous, and further mechanistic, imaging, and phenotyping studies are needed to determine whether and how the infection–HF relationship differs across subtypes.

We found a substantial increase in HF risk during the first 6 months after ID hospitalization though the risk declined progressively over time. These findings align with previous studies, such as those by Sipilä *et al*.,^25^ which revealed significant short-term links between respiratory, urinary tract, and intestinal infections and major cardiovascular events, with a slight increased risk in the long term. However, a study involving 4389 hospitalized medical patients in Northern Denmark found no change in cardiovascular risk over 6 months following bacteraemia.^26^

The significant interaction between ID and AF on HF risk observed in our study aligns with existing evidence that AF amplifies cardiovascular risks in various contexts.^27–29^ Atrial fibrillation has been reported to increase haemodynamic stress,^27^ potentially exacerbating the above-mentioned myocardial dysfunction caused by ID. The interplay between ID and AF suggests that targeted therapeutic strategies may help prevent HF in this vulnerable population.

Furthermore, our study extends these findings to the general population in the absence of overt infection by showing that the PRS for ID in ID-free individuals is also associated with a higher risk of HF and adverse cardiac phenotypes. An integrative PRS, representing multiple markers or diseases, is more effective in disease prediction than a PRS for a single marker.^30^ Thus, applying ID-PRS for HF risk stratification may help identify individuals at high HF risk who may not be detected with HF-PRS alone. Notably, PRS related to TGF-β and acute inflammatory pathways showed similar results. Deteriorating diastolic function, a feature of cardiac ageing, is driven by fibrosis, with TGF-β as a key mediator.^31^ Elevated TGF-β production has been observed in the myocardium of HF patients from various causes and in several animal models where ischaemia triggered HF.^32^ Moreover, variants in the TGF-β gene, *TGFB1*, influence vulnerability to ID.^33^ Hence, the ID-PRS in our study should be viewed as exploratory tools that may, in the future, complement conventional HF risk scores. If validated in further studies, such scores could help to identify individuals with heightened infection-related susceptibility to adverse cardiac remodelling and HF and to prioritize them for intensified prevention or for enrolment into trials of targeted anti-inflammatory or anti-fibrotic interventions.

The precise mechanism linking ID to HF remains unclear, though several potential pathways have been proposed. First, infections activate the innate immune system, leading to a cytokine storm and excessive inflammatory responses,^34^ which trigger cardiomyocyte apoptosis and activate fibrotic pathways like TGF-β,^35^ promoting HF development. Second, direct infection by pathogens, such as viral myocarditis, causes damage to cardiomyocytes and cardiac remodelling, impairing heart function. Third, infection-induced metabolic dysregulation,^36^ particularly oxidative stress and mitochondrial dysfunction, further disrupts myocardial energy metabolism, accelerating HF progression.

### Strengths and limitations of this study

The strengths of this study include the following: first, a comprehensive assessment of hospitalizations for more than 900 IDs from two large-scale nationwide cohorts in the UK and USA, with generally consistent findings across the cohorts, strengthening the robustness and generalizability of our results. Second, we incorporated detailed cardiac imaging analyses, which allowed us to identify structural and functional cardiac differences associated with ID. Third, we explored genetic impact of PRS for ID in an ID-free population, revealing an association of genetic susceptibility to ID with HF and adverse cardiac phenotypes, providing new insights into the underlying mechanisms of infection-related HF. This approach could avoid potential confounding factors, such as secondary effects of dietary changes or the use of anti-infective agents.

Nevertheless, we acknowledge several limitations. First, focusing solely on severe, hospital-treated infections may have led to an overestimation of the relative risk of major cardiovascular events linked to infections while underestimating the total contribution of infections to the HF burden, as milder infections—representing the majority—were not captured. Additionally, unrecognized HF events may not have been captured, and some misclassification cannot be excluded. Second, although we excluded individuals with prevalent HF at baseline and modelled infections as time-varying exposures, the observational and largely cross-sectional nature of the imaging analyses means that we cannot definitively determine the sequence of infection and cardiac changes, and some degree of reverse causality remains possible. Third, the PRS analyses were conducted exclusively in individuals of European descent and should be regarded as exploratory and hypothesis-generating, limiting their generalizability to other racial and ethnic groups and to populations with different characteristics. Fourth, socio-economic status and lifestyle behaviours are likely to play an important role in shaping both the occurrence of serious infections and the subsequent risk of HF. Individuals with lower socio-economic status and adverse lifestyle profiles may be more exposed to infection risk factors, have delayed access to care, and also carry a higher burden of cardiometabolic disease. In our analyses, we adjusted for education and several key lifestyle-related factors, and in UKB, we additionally showed that further adjustment for the Townsend Deprivation Index and household income did not materially change the results. Nevertheless, residual confounding by socio-economic status and lifestyle cannot be ruled out and may partly contribute to the observed associations. Fifth, we did not have detailed, time-resolved data on specific anti-inflammatory therapies (e.g. corticosteroids or biologic agents targeting TGF-β and related pathways) or on the composition and duration of anti-infective regimens during and after hospital-treated IDs. Some of these agents are known to influence cardiac conduction, repolarization, haemodynamics, or myocardial function, and we were therefore unable to disentangle the effects of the infectious process itself from those of the drugs used to treat it. Treatment regimens also vary between pathogen groups; however, in our analyses, the associations with HF were broadly similar and of relatively strong magnitude across bacterial, viral, fungal, and parasitic infections and in both cohorts, suggesting that specific drug classes are unlikely to be the sole explanation for the overall pattern. These unmeasured treatment patterns may nevertheless have contributed to our findings to some extent and warrant investigation in future work using richer medication data.

## Conclusions

The results showed that ID was linked to an increased risk of HF, regardless of HFpEF or HFrEF, and associated with cardiovascular remodelling. In participants without overt infection, a higher PRS for ID was associated with increased HF risk and adverse cardiac phenotypes, with the involvement of TGF-β and inflammatory pathways. Our results suggest that integrating prior ID status and PRS for ID into HF screening has the potential to help identify high-risk individuals. Further trials and pharmacological studies are needed to clarify the mechanisms linking fibro-inflammatory pathway-specific PRS for ID with HF and explore potential risk reduction interventions.

## Supplementary Material

oeag036_Supplementary_Data

## Data Availability

Data are available in a public, open access repository. Data from the UK Biobank and ARIC are available to researchers on application.

## Additional information

This research has been conducted using the UK Biobank Resource under application no. 97089.
