# Supplementary material for: Hospital-treated infectious diseases and polygenic susceptibility in relation to heart failure and cardiac remodelling: evidence from the UK Biobank and ARIC cohorts
Source: Eur Heart J Open. 2026 Feb 21;6(3):oeag036. doi: 10.1093/ehjopen/oeag036 (PMC13262538; doi:10.1093/ehjopen/oeag036)
Supplement: oeag036_Supplementary_Data [file oeag036_supplementary_data.docx]

*Supplemental Materials*

CONTENTS

[eMethods 2](#_Toc214207102)

[Supplementary Table 1. Baseline characteristics according to infection category in the UK Biobank (2006-2010) and ARIC study (visit 4). 5](#_Toc214207103)

[Supplementary Table 2. Association between different types of infectious diseases and heart failure in the UK biobank and ARIC study. 6](#_Toc214207104)

[Supplementary Table 3. Sensitivity analyses for the association between infectious disease and incident heart failure. 7](#_Toc214207105)

[Supplementary Table 4. Baseline characteristics according to tertiles of pathway-based polygenetic score in the UK Biobank (2006-2010) and ARIC study (visit 4). 8](#_Toc214207106)

[Supplementary Table 5. Association between pathway-based polygenetic risk score for infectious disease and heart failure in the UK biobank and ARIC study. 12](#_Toc214207107)

[Supplementary Table 6. Baseline characteristics of participants included in analysis 4 in the UK Biobank (CMR sub-study) and ARIC study (visit 5). 14](#_Toc214207108)

[Supplementary Table 7. Association between infectious diseases and cardiovascular measurements in the UK biobank (CMR sub study) and ARIC study (visit 5). 15](#_Toc214207109)

[Supplementary Table 8. Baseline characteristics of participants included in analysis 5 in the UK Biobank (CMR sub-study) and ARIC study (visit 5). 16](#_Toc214207110)

[Supplementary Table 9. Association between pathway-based PRS for infectious disease and cardiovascular measurements in the UK biobank (CMR sub study) and ARIC study (visit 5). 17](#_Toc214207111)

[Supplementary Figure 1. Distribution density plots of pathway-based polygenetic risk score in UK Biobank and Atherosclerosis Risk in Communities (ARIC) study across different stages of analysis 18](#_Toc214207112)

[Supplementary Figure 2. Subgroup analyses of infectious diseases and risk of heart failure in the UK biobank and ARIC study 19](#_Toc214207113)

[Supplementary Table 10. Classification of type-specific hospital-treated infectious diseases in the study on diagnosis level 20](#_Toc214207114)

[All infectious diseases 20](#_Toc214207115)

[All bacterial infections 47](#_Toc214207116)

[All viral infections 61](#_Toc214207117)

[Fungal infections (mycoses) 66](#_Toc214207118)

[Parasitic infections 68](#_Toc214207119)

# eMethods

**Cardiac magnetic resonance imaging protocol and analysis (UKB)**

Recruitment for the imaging subsection of the UKB was conducted through a randomized invitation strategy.^1^ Detailed previously, the standardized protocol for Cardiac Magnetic Resonance (CMR) acquisition employs 1.5-Tesla Siemens Healthineers scanners (Erlangen, Germany).^2^ Short-axis cine stacks were analyzed through automated segmentation utilizing a deep learning neural network, which has achieved parity in performance with human experts and has been specifically optimized for UKB image datasets.^3^ Subsequent to segmentation, these images were subjected to post-processing to quantify biventricular volumes at end-systole and end-diastole, as well as stroke volumes, adhering to standardized protocols.^4^ Although cardiac MRI is highly accurate and reproducible in measuring absolute cardiac structure sizes, these measurements are significantly influenced by body size.^5^ To evaluate relative differences independent of body size, measurements are commonly adjusted by indexing to body surface area (BSA), which is determined using the Mosteller formula.^6^ Raw values of cardiac phenotypes are then indexed by division by BSA.

**Echocardiography in Atherosclerosis Risk in Communities (ARIC) study**

In the ARIC study, echocardiographic assessments during visit 5 were executed as per protocols outlined in earlier documentation. These assessments were uniformly performed at four designated field centers by sonographers who had undergone specific training and certification in the ARIC imaging protocol. Each center utilized the same model of echocardiographic equipment (Philips iE33) and probes (Philips XMatrix), provided by Koninklijke Philips, The Netherlands. Quantitative echocardiographic measurements were centrally processed in a blinded manner at the Brigham and Women’s Hospital’s echocardiography core laboratory in Boston, MA, USA, following the American Society of Echocardiography’s standards.

**Genotyping and imputation processing**

Genotyping, imputation, and quality control procedures for the UK Biobank have been previously documented.^7^ In brief, the UK Biobank processed 487,409 blood samples using two distinct genotyping platforms: the Applied Biosystems UK BiLEVE Axiom Array and the Applied Biosystems UKB Axiom Array, both products of Affymetrix under Thermo Fisher Scientific, Waltham, MA. Genome-wide imputation was conducted by the UK Biobank leveraging the reference panels from both the 1000 Genomes Project Phase 3 and the UK10K Project.

The ARIC study's procedures for genotyping, imputation, and quality control have been documented previously.^8^ To genotype genomic DNA from whole blood, the Genome-Wide Human SNP Array 6.0 from Affymetrix, USA, was utilized. To expand the set of genetic markers, race-specific imputation to the TOPMed reference panel (freeze 5b) was conducted.^9^ Individuals identified as first-degree relatives, genetic outliers, or those with inconsistent genotype data across platforms were excluded from the dataset before imputation. Principal components analysis using EIGENSTRAT was then performed on the GWAS data to identify population substructure or genetic ancestry.^10^

**Derivation of pathway-based PRS for ID**

Previously established protocols for genotyping, imputation, and quality control were followed for both the UKB and ARIC. An SNP set was constructed using Plink (version 1.9), achieving approximate linkage equilibrium through linkage disequilibrium pruning with an r² less than 0.25 within a 200-kb window. Using PRSice-2, we derived the marker weights and p-value association statistics for individual single-nucleotide polymorphisms (SNPs) from an earlier genome-wide association study (GWAS) focused on IDs in White populations.^12^ A total of 11,762,722 SNPs were used in the microarray for the PRS. We calculated the PRS for ID using a weighted method, where each SNP was coded as 0, 1, or 2 based on the number of risk alleles. Eighteen SNPs significantly associated with ID (P < 5×10⁻⁶) were weighted by their β coefficients. The PRS was generated using the formula: PRS = β₁ × SNP₁ + β₂ × SNP₂ + ... + βₙ × SNPₙ, where SNPᵢ represents the count of risk alleles for each SNP.^20^ A higher PRS indicates greater genetic susceptibility to ID. Participants were classified into "low" (lowest tertile), "medium" (medium tertile), or "high" (highest tertile) PRS groups. For the PRS-related analysis, we have exclusively included individuals of White descent, as the SNPs with significant influence on ID were derived from this population.

To elucidate the fibro-inflammatory processes connecting the PRS for ID with cardiac changes, we utilized genetic pathways from the Molecular Signatures Database (version 7.4)^21^ related to transforming growth factor-beta (TGF-β) signaling,^22^ myocardial fibrosis and acute inflammation in calculating the pathway-based PRS. Gene-set-based PRSs for ID were calculated using PRSet in PRSice-2, targeting genetic pathways associated with acute inflammation, TGF-β signaling, and myocardial fibrosis. We set a p-value threshold of 1.00 for PRSet^23^ due to the limited proportion of SNPs in gene-set PRS, which might not accurately represent the entire gene sets.

**Molecular Signatures Database (version 7.4) gene pathways used in pathway-based analyses**

BIOCARTA_TGFB_PATHWAY > TGF beta signaling pathway APC CDH1 CREBBP EP300 MAP2K1 MAP3K7 MAPK3 SKIL SMAD2 SMAD3 SMAD4 SMAD7 TAB1 TGFB1 TGFB2 TGFB3 TGFBR1 TGFBR2 ZFYVE9

HP_MYOCARDIAL_FIBROSIS > Myocardial fibrosis ALMS1 FKTN FLNC JPH2 KIF20A LAMP2 MYPN PPA2 RPL3L SERPINE1 TNNI3 TNNT2 TRIM37

GOBP_ACUTE_INFLAMMATORY_RESPONSE > Inflammation which comprises a rapid, short-lived, relatively uniform response to acute injury or antigenic challenge and is characterized by accumulations of fluid, plasma proteins, and granulocytic leukocytes. An acute inflammatory response occurs within a matter of minutes or hours, and either resolves within a few days or becomes a chronic inflammatory response. [GO_REF:0000022, GOC:add, ISBN:0781735149] A2M ACVR1 ADAM8 ADORA1 ADRA2A AHSG ALOX5AP ANO6 APCS APOA2 APOL2 ASH1L ASS1 B4GALT1 BTK C2CD4A C2CD4B C3 CCR7 CD163 CD6 CEBPB CNR1 CREB3L3 CRP CTNNBIP1 DNASE1 DNASE1L3 EDNRB EIF2AK1 ELANE EPO EXT1 F12 F2 F3 F8 FCGR1A FCGR2B FCGR3A FFAR2 FFAR3 FN1 FUT7 GATA3 GSTP1 HAMP HLA-E HP HPR IGHG1 IL1A IL1B IL20RB IL22 IL31RA IL4 IL6 IL6R IL6ST INS ITIH4 KL KLKB1 LBP MBL2 MIR92A1 MRGPRX1 MYLK3 NLRP3 NLRP6 NPY NPY5R NUPR1 OGG1 OPRM1 ORM1 ORM2 OSM OSMR PARK7 PIK3CG PLA2G2D PLSCR1 PRCP PTGER3 PTGES PTGS2 REG3A REG3G RHBDD3 S100A8 SAA1 SAA2 SAA4 SELENOS SERPINA1 SERPINA3 SERPINF2 SIGIRR TACR1 TFR2 TFRC TNF TNFRSF11A TNFSF11 TNFSF4 TREM1 TRPV1 UGT1A1 VCAM1 VNN1 ZP3

# Supplementary Table 1. Baseline characteristics according to infection category in the UK Biobank (2006-2010) and ARIC study (visit 4).

|  | **UKB** | | | | | **ARIC** | | | | |
| --- | --- | --- | --- | --- | --- | --- | --- | --- | --- | --- |
|  | **Bacterial** | **Viral** | **Fungal** | **Parasitic** | **P value** | **Bacterial** | **Viral** | **Fungal** | **Parasitic** | **P value** |
| **No. of participants** | 72659 | 8547 | 6626 | 4285 |  | 2876 | 485 | 398 | 418 |  |
| **Age** | 58.1 (8.0) | 57.8 (8.3) | 58.2 (8.3) | 58.0 (8.1) | <0.001 | 63.4 (5.6) | 63.1 (5.5) | 63.6 (5.8) | 63.3 (5.6) | <0.001 |
| **Women** | 39599 (54.5) | 4718 (55.2) | 3724 (56.2) | 2421 (56.5) | 0.004 | 1705 (59.3) | 290 (59.7) | 224 (56.2) | 255 (61.1) | 0.563 |
| **White** | 68299 (94.0) | 8128 (95.1) | 6129 (92.5) | 4058 (94.7) | <0.001 | 2085 (72.5) | 384 (79.2) | 291 (73.0) | 302 (72.3) | 0.021 |
| **More than high school** | 32551 (44.8) | 3692 (43.2) | 2902 (43.8) | 1838 (42.9) | 0.002 | 906 (31.5) | 151 (31.2) | 134 (33.7) | 119 (28.5) | 0.449 |
| **Current smoker** | 9736 (13.4) | 1068 (12.5) | 875 (13.2) | 510 (11.9) | 0.006 | 452 (15.7) | 73 (15.0) | 62 (15.5) | 73 (17.4) | 0.776 |
| **Never drinker** | 23542 (32.4) | 2718 (31.8) | 2173 (32.8) | 1440 (33.6) | 0.196 | 618 (21.5) | 85 (17.5) | 103 (25.8) | 96 (22.9) | 0.022 |
| **Height, cm** | 169.8 (10.2) | 170.2 (11.0) | 169.5 (13.1) | 170 (12.4) | <0.001 | 168.7 (9.6) | 168.6 (9.8) | 168.5 (9.7) | 168.6 (9.8) | 0.082 |
| **Weight, kg** | 76.2 (15.6) | 76.4 (15.2) | 75.8 (16.8) | 75.5 (15.9) | <0.001 | 77.6 (16.7) | 77.9 (16.9) | 78.2 (17.0) | 78.1 (16.8) | 0.030 |
| **Diabetes** | 3924 (5.4) | 487 (5.7) | 318 (4.8) | 227 (5.3) | 0.102 | 400 (13.9) | 65 (13.5) | 62 (15.5) | 44 (10.6) | 0.179 |
| **Hypertension** | 42142 (58.0) | 4974 (58.2) | 3896 (58.8) | 2455 (57.3) | 0.443 | 1240 (43.1) | 225 (46.3) | 208 (52.2) | 175 (41.8) | 0.003 |
| **Chronic kidney disease** | 945 (1.3) | 145 (1.7) | 80 (1.2) | 64 (1.5) | 0.013 | 55 (1.9) | 14 (2.8) | 17 (4.2) | 4 (1.0) | 0.004 |
| **Atrial fibrillation** | 872 (1.2) | 94 (1.1) | 106 (1.6) | 43 (1.0) | 0.012 | 95 (3.3) | 30 (6.1) | 10 (2.5) | 16 (3.9) | 0.009 |
| **Coronary heart disease** | 2906 (4.0) | 385 (4.5) | 245 (3.7) | 180 (4.2) | 0.061 | 244 (8.5) | 41 (8.5) | 31 (7.8) | 51 (12.2) | 0.072 |
| **Systolic blood pressure, mmHg** | 138.6 (18.8) | 139.3 (19.1) | 139.0 (18.7) | 138.4 (19.6) | <0.001 | 126.1 (19.3) | 126.5 (19.5) | 126.3 (19.2) | 126.6 (19.3) | 0.155 |
| **Diastolic blood pressure, mmHg** | 82.8 (10.2) | 82.4 (11.2) | 83.6 (13.2) | 83.0 (12.6) | <0.001 | 71.5 (10.4) | 71.9 (10.5) | 71.8 (10.8) | 71.7 (10.8) | 0.237 |
| **HbA1c, %** | 5.6 (0.7) | 5.5 (0.8) | 5.6 (0.8) | 5.6 (0.7) | 0.311 | 5.8 (0.7) | 5.8 (0.7) | 5.9 (0.9) | 5.7 (0.8) | 0.588 |
| **LDL-C, mmol/L** | 3.57 (2.2) | 3.56 (2.3) | 3.52 (2.1) | 3.61 (2.7) | <0.001 | 3.25 (0.9) | 3.22 (1.0) | 3.24 (1.1) | 3.18 (1.3) | 0.143 |
| **Triglycerides, mmol/L** | 1.50 (1.2) | 1.46 (1.6) | 1.44 (2.1) | 1.58 (2.0) | <0.001 | 1.52 (0.8) | 1.48 (0.8) | 1.55 (1.0) | 1.51 (0.8) | 0.036 |
| **eGFR, mL/min/1.73 m^2^** | 95.5 (14.7) | 94.7 (16.2) | 95.1 (15.0) | 95.6 (14.8) | <0.001 | 86.4 (14.3) | 86.3 (14.5） | 85.7 (15.0) | 85.3 (15.4) | 0.011 |

Data are presented as No. (%) or mean (SD). ID, infectious disease; HbA1c, hemoglobin A1c; LDL-C, low-density lipoprotein cholesterol; eGFR, estimated glomerular filtration rate. * P values were obtained from either a chi-square test or a Mann-Whitney U test comparing difference between the ID and No ID group.

# Supplementary Table 2. Association between different types of infectious diseases and heart failure in the UK biobank and ARIC study.

|  | **Number of event/ person years** | **Total number** | **HR (95% CI) for model 1** | **P value for model 1** | **HR (95% CI) for model 2** | **P value for model 2** |
| --- | --- | --- | --- | --- | --- | --- |
| **UK Biobank** |  |  |  |  |  |  |
| **Non-infection group** | 4676/5334686 | 389896 | **Reference** |  | **Reference** |  |
| **Any infectious disease** | 1808/1159424 | 90258 | 1.81 (1.72-1.92) | <0.001 | 1.54 (1.46-1.63) | <0.001 |
| **Bacterial infections** | 1565/992190 | 77659 | 1.84 (1.74-1.95) | <0.001 | 1.55 (1.47-1.65) | <0.001 |
| **Viral infections** | 181/115003 | 8879 | 1.83 (1.57-2.12) | <0.001 | 1.51 (1.30-1.75) | <0.001 |
| **Fungal infections** | 171/87854 | 6614 | 2.22 (1.91-2.59) | <0.001 | 1.98 (1.70-2.31) | <0.001 |
| **Parasitic infections** | 114/52340 | 4080 | 2.53 (2.10-3.05) | <0.001 | 2.14 (1.78-2.58) | <0.001 |
| **ARIC** |  |  |  |  |  |  |
| **Non-infection group** | 1006/142219 | 5962 | **Reference** |  | **Reference** |  |
| **Any infectious disease** | 1056/86560 | 3854 | 1.95 (1.78-2.12) | <0.001 | 1.84 (1.68-2.00) | <0.001 |
| **Bacterial infections** | 759/64336 | 2876 | 1.67 (1.53-1.83) | <0.001 | 1.62 (1.48-1.77) | <0.001 |
| **Viral infections** | 174/11019 | 485 | 2.08 (1.78-2.43) | <0.001 | 1.93 (1.65-2.26) | <0.001 |
| **Fungal infections** | 165/8931 | 398 | 2.35 (2.01-2.76) | <0.001 | 2.19 (1.87-2.57) | <0.001 |
| **Parasitic infections** | 163/9338 | 418 | 2.09 (1.78-2.45) | <0.001 | 1.91 (1.63-2.25) | <0.001 |

Model 1 was adjusted for sex and age (time scale). Model 2 were adjusted for sex, age (time scale), ethnicity, level of education, smoking status, alcohol drinking, height, weight, hypertension, diabetes, chronic kidney disease and low-density lipoprotein cholesterol. HR = hazard ratio. CI = confidence interval.

# Supplementary Table 3. Sensitivity analyses for the association between infectious disease and incident heart failure.

|  | **Any infectious disease** | | **Bacterial infections** | | **Viral infections** | | **Fungal infections** | | **Parasitic infections** | |
| --- | --- | --- | --- | --- | --- | --- | --- | --- | --- | --- |
|  | **HR (95%CI)** | **P value** | **HR (95%CI)** | **P value** | **HR (95%CI)** | **P value** | **HR (95%CI)** | **P value** | **HR (95%CI)** | **P value** |
| **UK Biobank** |  |  |  |  |  |  |  |  |  |  |
| **Multivariable model 2 ^a^** | 1.55 (1.47-1.65) | <0.001 | 1.57 (1.48-1.65) | <0.001 | 1.53 (1.32-1.76) | <0.001 | 2.01 (1.71-2.32) | <0.001 | 2.16 (1.81-2.60) | <0.001 |
| **Multivariable model 2 ^b^** | 1.51 (1.42-1.59) | <0.001 | 1.52 (1.44-1.62) | <0.001 | 1.48 (1.28-1.73) | <0.001 | 1.93 (1.65-2.28) | <0.001 | 2.07 (1.70-2.51) | <0.001 |
| **Multivariable model 2 ^c^** | 1.43 (1.33-1.52) | <0.001 | 1.45 (1.33-1.53) | <0.001 | 1.42 (1.17-1.68) | <0.001 | 1.79 (1.42-2.15) | <0.001 | 2.03 (1.55-2.51) | <0.001 |
| **Multivariable model 2 ^d^** | 1.45 (1.34-1.54) | <0.001 | 1.46 (1.34-1.54) | <0.001 | 1.40 (1.16-1.65) | <0.001 | 1.85 (1.46-2.23) | <0.001 | 1.89 (1.44-2.33) | <0.001 |
| **Multivariable model 2 ^e^** | 1.49 (1.40-1.1.58) | <0.001 | 1.52 (1.42-1.62) | <0.001 | 1.47 (1.25-1.69) | <0.001 | 1.88 (1.61-2.17) | <0.001 | 1.96 (1.61-2.35) | <0.001 |
| **ARIC** |  |  |  |  |  |  |  |  |  |  |
| **Multivariable model 2 ^f^** | 1.86 (1.71-2.03) | <0.001 | 1.67 (1.54-1.80) | <0.001 | 1.94 (1.66-2.27) | <0.001 | 2.22 (1.90-2.59) | <0.001 | 1.93 (1.65-2.26) | <0.001 |
| **Multivariable model 2 ^b^** | 1.76 (1.62-1.91) | <0.001 | 1.59 (1.42-1.73) | <0.001 | 1.81 (1.52-2.13) | <0.001 | 2.01 (1.71-2.42) | <0.001 | 1.79 (1.50-2.11) | <0.001 |
| **Multivariable model 2 ^g^** | 1.78 (1.58-2.01) | <0.001 | 1.60 (1.42-1.79) | <0.001 | 1.84 (1.50-2.17) | <0.001 | 2.10 (1.66-2.60) | <0.001 | 1.83 (1.44-2.23) | <0.001 |
| **Multivariable model 2 ^h^** | 1.71 (1.52-1.91) | <0.001 | 1.52 (1.33-1.70) | <0.001 | 1.78 (1.37-2.15) | <0.001 | 1.99 (1.48-2.51) | <0.001 | 1.76 (1.32-2.18) | <0.001 |

Model 2 were adjusted for sex, age (time scale), ethnicity, level of education, smoking status, alcohol drinking, height, weight, hypertension, diabetes, chronic kidney disease and low-density lipoprotein cholesterol. HR = hazard ratio. CI = confidence interval.

a Used Fine & Gray Models for Competing Risk. There were 19,521 deaths as competing events for heart failure.

b Added adjustment for coronary heart disease and atrial fibrillation.

c After excluding patients infected with multiple types of pathogens, the analysis Included 83,883, 72,258, 5,588, 3,790, 2,247 cases for any infectious disease, bacterial infections, viral infections, fungal infections and parasitic infections.

d Excluded 81,240 individuals without any hospitalization records.

e In addition to the variables in Model 2, the model was further adjusted for the Townsend Deprivation Index (TDI) and annual household income.

f Used Fine & Gray Models for Competing Risk. There were 375 deaths as competing events for heart failure.

g After excluding patients infected with multiple types of pathogens, the analysis Included 2,968, 2,228, 271, 207, 262 cases for any infectious disease, bacterial infections, viral infections, fungal infections and parasitic infections.

h Excluded 1,816 individuals without any hospitalization records.

# Supplementary Table 4. Baseline characteristics according to tertiles of pathway-based polygenetic score in the UK Biobank (2006-2010) and ARIC study (visit 4).

|  | **UKB** | | | | | **ARIC** | | | | |
| --- | --- | --- | --- | --- | --- | --- | --- | --- | --- | --- |
|  | **Overall** | **Low PRS** | **Medium PRS** | **High PRS** | **P Value** | **Overall** | **Low PRS** | **Medium PRS** | **High PRS** | **P Value** |
| **PRS (All)** |  |  |  |  |  |  |  |  |  |  |
| **No. of participants** | 331411 | 110471 | 110470 | 110470 |  | 5203 | 1735 | 1734 | 1734 |  |
| **Age** | 55.9 (8.0) | 55.7 (8.1) | 55.8 (7.9) | 56.1 (8.0) | <0.001 | 62.7 (5.5) | 62.5 (5.6) | 62.8 (5.6) | 62.7 (5.5) | 0.216 |
| **Women** | 185614 (56.0) | 61888 (56.0) | 62084 (56.2) | 61642 (55.8) | 0.165 | 3007 (57.8) | 1010 (58.2) | 995 (57.4) | 1002 (57.8) | 0.969 |
| **More than high school** | 155984 (47.1) | 52032 (47.1) | 52252 (47.3) | 51700 (46.8) | 0.0605 | 1738 (33.4) | 567 (32.7) | 595 (34.3) | 576 (33.2) | 0.582 |
| **Current smoker** | 32091 (9.7) | 10627 (9.6) | 10638 (9.6) | 10826 (9.8) | 0.273 | 806 (15.5) | 281 (16.2) | 251 (14.5) | 274 (15.8) | 0.541 |
| **Never drinker** | 99422 (30.0) | 32699 (29.6) | 33472 (30.3) | 33251 (30.1) | 0.0011 | 1061 (20.4) | 383 (22.1) | 310 (17.9) | 368 (21.2) | 0.014 |
| **Height, cm** | 169.6 (9.7) | 169.5 (10.1) | 169.8 (9.7) | 169.6 (9.9) | <0.001 | 168.9 (9.6) | 169.1 (9.7) | 168.6 (9.6) | 168.9 (9.7) | <0.001 |
| **Weight, kg** | 75.9 (15.2) | 75.7 (15.4) | 75.9 (15.2) | 76.0 (15.1) | <0.001 | 78.1 (16.7) | 78.2 (16.6) | 78.0 (16.8) | 78.0 (16.7) | 0.102 |
| **Diabetes** | 16902 (5.1) | 5303 (4.8) | 5744 (5.2) | 5855 (5.3) | <0.001 | 656 (12.6) | 196 (11.3) | 168 (9.7) | 292 (16.8) | <0.001 |
| **Hypertension** | 185370 (55.9) | 61643 (55.8) | 61974 (56.1) | 61753 (55.9) | 0.351 | 2190 (42.1) | 652 (37.6) | 707 (40.8) | 831 (47.9) | <0.001 |
| **CKD** | 4419 (1.3) | 1657 (1.5) | 1326 (1.2) | 1436 (1.3) | <0.001 | 109 (2.1) | 14 (0.8) | 43 (2.5) | 52 (3.0) | <0.001 |
| **Atrial fibrillation** | 3226 (1.0) | 773 (0.7) | 1127 (1.0) | 1326 (1.2) | <0.001 | 182 (3.5) | 36 (2.1) | 76 (4.4) | 70 (4.0) | <0.001 |
| **Coronary heart disease** | 12041 (3.6) | 3535 (3.2) | 4529 (4.1) | 3977 (3.6) | <0.001 | 552 (10.6) | 203 (11.7) | 127 (7.3) | 222 (12.8) | <0.001 |
| **Systolic blood pressure, mmHg** | 138.0 (18.2) | 137.8 (18.3) | 138.0 (18.1) | 138.2 (18.5) | <0.001 | 125.6 (18.7) | 125.8 (18.7) | 125.5 (18.6) | 125.5 (18.7) | 0.027 |
| **Diastolic blood pressure, mmHg** | 82.3 (10.1) | 82.6 (10.1) | 82.3 (10.3) | 82.0 (10.1) | <0.001 | 70.8 (10.1) | 71.0 (10.2) | 70.8 (10.1) | 70.7 (9.9) | 0.510 |
| **HbA1c, %** | 5.5 (0.6) | 5.6 (0.7) | 5.4 (0.6) | 5.5 (0.6) | <0.001 | 5.7 (0.5) | 5.8 (0.5) | 5.7 (0.6) | 5.7 (0.5) | 0.088 |
| **LDL-C, mmol/L** | 3.54 (1.7) | 3.55 (1.7) | 3.53 (1.8) | 3.55 (1.8) | 0.136 | 3.13 (1.0) | 3.16 (0.9) | 3.09 (0.9) | 3.12 (1.0) | 0.055 |
| **Triglycerides, mmol/L** | 1.48 (0.9) | 1.50 (1.0) | 1.47 (0.8) | 1.48 (0.8) | <0.001 | 1.53 (0.8) | 1.55 (1.0) | 1.52 (0.9) | 1.53 (0.9) | 0.410 |
| **eGFR, mL/min/1.73 m2** | 95.7 (14.1) | 95.9 (14.1) | 95.7 (14.0) | 95.6 (14.5) | <0.001 | 87.0 (14.3) | 86.7 (14.5) | 87.1 (14.2) | 87.2 (14.4) | 0.311 |
| **PRS (TGF-β)** |  |  |  |  |  |  |  |  |  |  |
| **No. of participants** | 331411 | 110471 | 110470 | 110470 |  | 5203 | 1735 | 1734 | 1734 |  |
| **Age** | 55.9 (8.0) | 55.6 (8.2) | 55.8 (8.0) | 56.2 (8.0) | <0.001 | 62.7 (5.5) | 62.5 (5.7) | 62.8 (5.6) | 62.8 (5.5) | 0.116 |
| **Women** | 185614 (56.0) | 61897 (56.0) | 62084 (56.2) | 61633 (55.8) | 0.152 | 3007 (57.8) | 965 (55.6) | 1025 (59.1) | 1017 (58.7) | 0.077 |
| **More than high school** | 155984 (47.1) | 52142 (47.2) | 52363 (47.4) | 51479 (46.6) | <0.001 | 1738 (33.4) | 633 (36.5) | 534 (30.8) | 571 (32.9) | 0.002 |
| **Current smoker** | 32091 (9.7) | 10495 (9.5) | 10605 (9.6) | 10991 (9.9) | <0.001 | 806 (15.5) | 245 (14.1) | 272 (15.7) | 289 (16.7) | 0.112 |
| **Never drinker** | 99422 (30.0) | 32589 (29.5) | 33583 (30.4) | 33250 (30.1) | <0.001 | 1061 (20.4) | 429 (24.7) | 305 (17.6) | 327 (18.9) | <0.001 |
| **Height, cm** | 169.6 (9.7) | 169.6 (10.0) | 169.7 (9.7) | 169.5 (10.1) | <0.001 | 168.9 (9.6) | 169.2 (9.8) | 169.0 (9.7) | 168.7 (9.7) | 0.088 |
| **Weight, kg** | 75.9 (15.2) | 75.7 (15.4) | 76.0 (15.1) | 76.0 (15.2) | <0.001 | 78.1 (16.7) | 78.1 (16.7) | 78.3 (17.0) | 77.8 (16.5) | 0.109 |
| **Diabetes** | 16902 (5.1) | 5535 (5.0) | 5634 (5.1) | 5733 (5.2) | 0.159 | 656 (12.6) | 142 (8.2) | 265 (15.3) | 249 (14.4) | <0.001 |
| **Hypertension** | 185370 (55.9) | 61753 (55.9) | 61532 (55.7) | 62085 (56.2) | 0.058 | 2190 (42.1) | 647 (37.3) | 725 (41.8) | 818 (47.2) | <0.001 |
| **CKD** | 4419 (1.3) | 1878 (1.7) | 1436 (1.3) | 1105 (1.0) | <0.001 | 109 (2.1) | 16 (0.9) | 43 (2.5) | 50 (2.9) | <0.001 |
| **Atrial fibrillation** | 3226 (1.0) | 994 (0.9) | 994 (0.9) | 1238 (1.1) | <0.001 | 182 (3.5) | 29 (1.7) | 71 (4.1) | 82 (4.7) | <0.001 |
| **Coronary heart disease** | 12041 (3.6) | 3425 (3.1) | 4441 (4.0) | 4175 (3.8) | <0.001 | 552 (10.6) | 187 (10.8) | 116 (6.7) | 249 (14.4) | <0.001 |
| **Systolic blood pressure, mmHg** | 138.0 (18.2) | 138.0 (18.4) | 138.0 (18.1) | 138.1 (18.4) | 0.264 | 125.6 (18.7) | 125.9 (18.6) | 125.5 (18.7) | 125.4 (18.7) | 0.064 |
| **Diastolic blood pressure, mmHg** | 82.3 (10.1) | 82.3 (10.1) | 82.2 (10.2) | 82.3 (10.1) | 0.410 | 70.8 (10.1) | 70.9 (10.2) | 71.9 (10.2) | 69.7 (9.9) | 0.072 |
| **HbA1c, %** | 5.5 (0.6) | 5.5 (0.7) | 5.4 (0.6) | 5.5 (0.6) | 0.355 | 5.7 (0.5) | 5.7 (0.5) | 5.7 (0.6) | 5.8 (0.6) | 0.102 |
| **LDL-C, mmol/L** | 3.54 (1.7) | 3.55 (1.7) | 3.54 (1.9) | 3.54 (1.8) | 0.711 | 3.13 (1.0) | 3.14 (0.9) | 3.11 (0.9) | 3.13 (1.1) | 0.080 |
| **Triglycerides, mmol/L** | 1.48 (0.9) | 1.51 (1.1) | 1.47 (0.8) | 1.47 (0.9) | <0.001 | 1.53 (0.8) | 1.54 (1.0) | 1.52 (1.0) | 1.55 (0.9) | 0.510 |
| **eGFR, mL/min/1.73 m2** | 95.7 (14.1) | 95.7 (14.0) | 95.9 (14.1) | 95.5 (14.5) | <0.001 | 87.0 (14.3) | 86.9 (14.4) | 87.0 (14.2) | 87.1 (14.5) | 0.774 |
| **PRS (Myocardial Fibrosis)** |  |  |  |  |  |  |  |  |  |  |
| **No. of participants** | 331411 | 110471 | 110470 | 110470 |  | 5203 | 1735 | 1734 | 1734 |  |
| **Age** | 55.9 (8.0) | 55.7 (8.1) | 55.8 (8.0) | 56.1 (8.1) | <0.001 | 62.7 (5.5) | 62.3 (5.6) | 62.5 (5.5) | 63.4 (5.7) | <0.001 |
| **Women** | 185614 (56.0) | 61974 (56.1) | 62084 (56.2) | 61556 (55.7) | 0.058 | 3007 (57.8) | 1025 (59.1) | 992 (57.2) | 990 (57.1) | 0.413 |
| **More than high school** | 155984 (47.1) | 51943 (47.0) | 52252 (47.3) | 51789 (46.9) | 0.132 | 1738 (33.4) | 505 (29.1) | 621 (35.8) | 612 (35.3) | <0.001 |
| **Current smoker** | 32091 (9.7) | 10274 (9.3) | 10716 (9.7) | 11101 (10.0) | <0.001 | 806 (15.5) | 264 (15.2) | 333 (19.2) | 209 (12.1) | <0.001 |
| **Never drinker** | 99422 (30.0) | 32589 (29.5) | 33362 (30.2) | 33471 (30.3) | <0.001 | 1061 (20.4) | 361 (20.8) | 430 (24.8) | 270 (15.6) | <0.001 |
| **Height, cm** | 169.6 (9.7) | 169.4 (10.2) | 169.8 (9.8) | 169.6 (10.0) | <0.001 | 168.9 (9.6) | 169.3 (9.8) | 168.9 (9.7) | 168.7 (9.7) | 0.101 |
| **Weight, kg** | 75.9 (15.2) | 75.8 (15.3) | 76.0 (15.1) | 75.9 (15.2) | <0.001 | 78.1 (16.7) | 78.3 (16.7) | 78.2 (17.1) | 77.7 (16.6) | 0.210 |
| **Diabetes** | 16902 (5.1) | 5744 (5.2) | 5556 (5.0) | 5602 (5.1) | 0.166 | 656 (12.6) | 172 (9.9) | 260 (15.0) | 224 (12.9) | <0.001 |
| **Hypertension** | 185370 (55.9) | 61864 (56.0) | 61653 (55.8) | 61853 (56.0) | 0.596 | 2190 (42.1) | 743 (42.8) | 690 (39.8) | 757 (43.7) | 0.053 |
| **CKD** | 4419 (1.3) | 1436 (1.3) | 1657 (1.5) | 1326 (1.2) | <0.001 | 109 (2.1) | 49 (2.8) | 29 (1.7) | 31 (1.8) | 0.033 |
| **Atrial fibrillation** | 3226 (1.0) | 884 (0.8) | 1143 (1.0) | 1199 (1.1) | <0.001 | 182 (3.5) | 43 (2.5) | 68 (3.9) | 71 (4.1) | 0.017 |
| **Coronary heart disease** | 12041 (3.6) | 3866 (3.5) | 4198 (3.8) | 3977 (3.6) | <0.001 | 552 (10.6) | 191 (11.0) | 153 (8.8) | 208 (12.0) | 0.008 |
| **Systolic blood pressure, mmHg** | 138.0 (18.2) | 138.2 (18.4) | 138.1 (18.2) | 137.9 (18.3) | <0.001 | 125.6 (18.7) | 126.1 (18.6) | 125.4 (18.8) | 125.3 (18.5) | 0.105 |
| **Diastolic blood pressure, mmHg** | 82.3 (10.1) | 82.1 (10.3) | 82.4 (10.2) | 82.3 (10.2) | <0.001 | 70.8 (10.1) | 71.2 (10.2) | 71.6 (10.3) | 69.9 (9.9) | 0.011 |
| **HbA1c, %** | 5.5 (0.6) | 5.5 (0.7) | 5.5 (0.6) | 5.4 (0.7) | <0.001 | 5.7 (0.5) | 5.6 (0.5) | 5.8 (0.7) | 5.8 (0.6) | 0.028 |
| **LDL-C, mmol/L** | 3.54 (1.7) | 3.54 (1.7) | 3.54 (1.8) | 3.55 (1.8) | 0.467 | 3.13 (1.0) | 3.12 (0.9) | 3.11 (1.0) | 3.14 (1.2) | 0.310 |
| **Triglycerides, mmol/L** | 1.48 (0.9) | 1.50 (1.2) | 1.48 (0.8) | 1.47 (1.0) | <0.001 | 1.53 (0.8) | 1.55 (1.1) | 1.52 (1.1) | 1.54 (0.9) | 0.411 |
| **eGFR, mL/min/1.73 m2** | 95.7 (14.1) | 95.6 (14.0) | 96.0 (14.2) | 95.6 (14.5) | <0.001 | 87.0 (14.3) | 86.8 (14.7) | 87.2 (14.2) | 87.0 (14.5) | 0.562 |
| **PRS (Acute Inflammation)** |  |  |  |  |  |  |  |  |  |  |
| **No. of participants** | 331411 | 110470 | 110471 | 110470 |  | 5203 | 1735 | 1734 | 1734 |  |
| **Age** | 55.9 (8.0) | 55.8 (8.2) | 55.8 (8.0) | 56.0 (8.1) | <0.001 | 62.7 (5.5) | 62.4 (5.7) | 62.7 (5.7) | 62.9 (5.4) | 0.019 |
| **Women** | 185614 (56.0) | 62195 (56.3) | 61974 (56.1) | 61445 (55.6) | 0.004 | 3007 (57.8) | 970 (55.9) | 1014 (58.5) | 1023 (59.0) | 0.142 |
| **More than high school** | 155984 (47.1) | 52142 (47.2) | 52031 (47.1) | 51811 (46.9) | 0.357 | 1738 (33.4) | 616 (35.5) | 550 (31.7) | 572 (33.0) | 0.055 |
| **Current smoker** | 32091 (9.7) | 10716 (9.7) | 10605 (9.6) | 10770 (9.7) | 0.481 | 806 (15.5) | 298 (17.2) | 264 (15.2) | 244 (14.1) | 0.038 |
| **Never drinker** | 99422 (30.0) | 32147 (29.1) | 33693 (30.5) | 33582 (30.4) | <0.001 | 1061 (20.4) | 274 (15.8) | 368 (21.2) | 419 (24.2) | <0.001 |
| **Height, cm** | 169.6 (9.7) | 169.2 (10.3) | 169.9 (9.8) | 169.7 (10.1) | <0.001 | 168.9 (9.6) | 169.2 (9.8) | 169.0 (9.7) | 168.7 (10.1) | 0.614 |
| **Weight, kg** | 75.9 (15.2) | 76.0 (15.1) | 75.8 (15.1) | 75.9 (15.3) | <0.001 | 78.1 (16.7) | 78.1 (16.7) | 78.3 (17.0) | 77.8 (17.5) | 0.358 |
| **Diabetes** | 16902 (5.1) | 6076 (5.5) | 5192 (4.7) | 5634 (5.1) | <0.001 | 656 (12.6) | 137 (7.9) | 224 (12.9) | 295 (17.0) | <0.001 |
| **Hypertension** | 185370 (55.9) | 62306 (56.4) | 61902 (56.0) | 61162 (55.4) | <0.001 | 2190 (42.1) | 762 (43.9) | 687 (39.6) | 741 (42.7) | 0.029 |
| **CKD** | 4419 (1.3) | 1988 (1.8) | 1215 (1.1) | 1216 (1.1) | <0.001 | 109 (2.1) | 59 (3.4) | 24 (1.4) | 26 (1.5) | <0.001 |
| **Atrial fibrillation** | 3226 (1.0) | 994 (0.9) | 1118 (1.0) | 1114 (1.0) | 0.009 | 182 (3.5) | 19 (1.1) | 47 (2.7) | 116 (6.7) | <0.001 |
| **Coronary heart disease** | 12041 (3.6) | 4198 (3.8) | 4971 (4.5) | 2872 (2.6) | <0.001 | 552 (10.6) | 88 (5.1) | 229 (13.2) | 235 (13.6) | <0.001 |
| **Systolic blood pressure, mmHg** | 138.0 (18.2) | 137.9 (18.3) | 138.2 (18.2) | 138.0 (18.2) | <0.001 | 125.6 (18.7) | 126.0 (18.5) | 125.6 (18.8) | 125.3 (18.3) | 0.290 |
| **Diastolic blood pressure, mmHg** | 82.3 (10.1) | 82.3 (10.2) | 82.1 (10.5) | 82.4 (10.2) | <0.001 | 70.8 (10.1) | 71.4 (10.2) | 71.5 (10.2) | 69.8 (10.1) | 0.059 |
| **HbA1c, %** | 5.5 (0.6) | 5.4 (0.8) | 5.5 (0.6) | 5.5 (0.7) | 0.153 | 5.7 (0.5) | 5.6 (0.7) | 5.6 (0.6) | 5.9 (0.6) | 0.002 |
| **LDL-C, mmol/L** | 3.54 (1.7) | 3.56 (1.7) | 3.53 (1.9) | 3.54 (1.8) | <0.001 | 3.13 (1.0) | 3.11 (0.9) | 3.13 (1.1) | 3.14 (1.2) | 0.772 |
| **Triglycerides, mmol/L** | 1.48 (0.9) | 1.48 (1.1) | 1.49 (0.8) | 1.48 (1.1) | 0.611 | 1.53 (0.8) | 1.54 (1.1) | 1.52 (1.2) | 1.55 (0.9) | 0.210 |
| **eGFR, mL/min/1.73 m2** | 95.7 (14.1) | 95.7 (14.3) | 95.7 (14.2) | 95.8 (14.1) | 0.510 | 87.0 (14.3) | 87.1 (14.9) | 87.1 (14.4) | 86.7 (14.4) | 0.089 |

Data are presented as No. (%) or mean (SD). ID, infectious disease; CKD, chronic kidney disease; CHD, coronary heart disease; HbA1c, hemoglobin A1c; LDL-C, low-density lipoprotein cholesterol; eGFR, estimated glomerular filtration rate. * P values were obtained from either a chi-square test or a Mann-Whitney U test comparing difference between tertiles of the PRS.

# Supplementary Table 5. Association between pathway-based polygenetic risk score for infectious disease and heart failure in the UK biobank and ARIC study.

|  | **Number of event/ person years** | **Total number** | **HR (95% CI) for model 1** | **P value for model 1** | **HR (95% CI) for model 2** | **P value for model 2** |
| --- | --- | --- | --- | --- | --- | --- |
| **UKB** |  |  |  |  |  |  |
| **PRS (All)** |  |  |  |  |  |  |
| Low | 1138/1430599 | 110471 | **Reference** |  | **Reference** |  |
| Medium | 1179/1422854 | 110470 | 1.08 (0.98-1.17) | 0.061 | 1.04 (0.95-1.14) | 0.112 |
| High | 1361/1427272 | 110470 | 1.32 (1.19-1.44) | <0.001 | 1.23 (1.12-1.35) | <0.001 |
| Per 1-SD increase |  |  | 1.11(1.07-1.16) | <0.001 | 1.07 (1.03-1.11) | 0.035 |
| **PRS (TGF-β)** |  |  |  |  |  |  |
| Low | 1129/1430578 | 110471 | **Reference** |  | **Reference** |  |
| Medium | 1197/1422879 | 110470 | 1.08 (0.99-1.17) | 0.053 | 1.03 (0.94-1.12) | 0.561 |
| High | 1352/1427268 | 110470 | 1.19 (1.08-1.29) | <0.001 | 1.12 (1.02-1.23) | 0.043 |
| per 1-SD increase |  |  | 1.07 (1.03-1.10) | 0.024 | 1.04 (1.00-1.07) | 0.046 |
| **PRS (Myocardial Fibrosis)** |  |  |  |  |  |  |
| Low | 1187/1430498 | 110471 | **Reference** |  | **Reference** |  |
| Medium | 1213/1422889 | 110470 | 1.05 (0.97-1.14) | 0.074 | 1.02 (0.94-1.11) | 0.595 |
| High | 1278/1427338 | 110470 | 1.12 (1.03-1.20) | 0.041 | 1.08 (0.99-1.16) | 0.069 |
| Per 1-SD increase |  |  | 1.06 (1.03-1.09) | 0.026 | 1.02 (0.99-1.05) | 0.282 |
| **PRS (Acute Inflammation)** |  |  |  |  |  |  |
| Low | 1183/1430610 | 110470 | **Reference** |  | **Reference** |  |
| Medium | 1170/1422812 | 110471 | 1.04 (0.96-1.13) | 0.077 | 1.03 (0.95-1.11) | 0.515 |
| High | 1325/1427303 | 110470 | 1.23 (1.14-1.32) | <0.001 | 1.19 (1.10-1.29) | <0.001 |
| Per 1-SD increase |  |  | 1.09 (1.06-1.13) | <0.001 | 1.04 (1.01-1.08) | 0.045 |
| **ARIC** |  |  |  |  |  |  |
| **PRS (All)** |  |  |  |  |  |  |
| Low | 294/38690 | 1735 | **Reference** |  | **Reference** |  |
| Medium | 314/38772 | 1734 | 1.07 (0.91-1.25) | 0.433 | 1.06 (0.91-1.24) | 0.463 |
| High | 348/38616 | 1734 | 1.23 (1.05-1.43) | 0.01 | 1.19 (1.02-1.39) | 0.025 |
| Per 1-SD increase |  |  | 1.12 (1.05-1.20) | <0.001 | 1.11 (1.04-1.19) | <0.001 |
| **PRS (TGF-β)** |  |  |  |  |  |  |
| Low | 286/38672 | 1735 | **Reference** |  | **Reference** |  |
| Medium | 332/38725 | 1734 | 1.18 (1.01-1.38) | 0.043 | 1.16 (0.99-1.36) | 0.073 |
| High | 338/38681 | 1734 | 1.21 (1.04-1.42) | 0.016 | 1.20 (1.03-1.41) | 0.021 |
| per 1-SD increase |  |  | 1.07 (1.01-1.14) | 0.043 | 1.06 (1.00-1.13) | 0.048 |
| **PRS (Myocardial Fibrosis)** |  |  |  |  |  |  |
| Low | 307/38655 | 1735 | **Reference** |  | **Reference** |  |
| Medium | 321/38719 | 1734 | 1.05 (0.90-1.23) | 0.523 | 1.08 (0.92-1.26) | 0.345 |
| High | 328/38704 | 1734 | 1.07 (0.91-1.25) | 0.411 | 1.11 (0.95-1.29) | 0.208 |
| Per 1-SD increase |  |  | 1.03 (0.97-1.10) | 0.111 | 1.05 (0.99-1.12) | 0.063 |
| **PRS (Acute Inflammation)** |  |  |  |  |  |  |
| Low | 291/38701 | 1735 | **Reference** |  | **Reference** |  |
| Medium | 313/38762 | 1734 | 1.09 (0.92-1.27) | 0.316 | 1.06 (0.91-1.25) | 0.445 |
| High | 352/38615 | 1734 | 1.25 (1.07-1.46) | 0.004 | 1.23 (1.05-1.43) | 0.01 |
| Per 1-SD increase |  |  | 1.10 (1.03-1.18) | 0.004 | 1.10 (1.03-1.17) | 0.004 |

Model 1 was adjusted for sex and age (time scale). Model 2 were adjusted for sex, age (time scale), level of education, smoking status, alcohol drinking, height, weight, hypertension, diabetes, chronic kidney disease and low-density lipoprotein cholesterol. HR = hazard ratio. CI = confidence interval

# Supplementary Table 6. Baseline characteristics of participants included in analysis 4 in the UK Biobank (CMR sub-study) and ARIC study (visit 5).

|  | **UKB** | **ARIC** | **P for difference between two cohorts** |
| --- | --- | --- | --- |
| **No. of participants** | 37602 | 4995 |  |
| **Age** | 64.1 (7.4) | 76.0 (5.1) | <0.001 |
| **Women** | 19666 (52.3) | 3307 (66.2) | <0.001 |
| **White** | 36399 (96.8) | 4251 (85.1) | <0.001 |
| **More than high school** | 12634 (33.6) | 1538 (30.8) | <0.001 |
| **Current smoker** | 2407 (6.4) | 440 (8.8) | <0.001 |
| **Never drinker** | 1730 (4.6) | 969 (19.4) | <0.001 |
| **Height, cm** | 169.1 (9.3) | 165.2 (8.7) | <0.001 |
| **Weight, kg** | 76.0 (15.1) | 63.1 (9.2) | <0.001 |
| **Diabetes** | 2068 (5.5) | 964 (19.3) | <0.001 |
| **Hypertension** | 28051 (74.6) | 3536 (70.8) | <0.001 |
| **CKD** | 865 (2.3) | 185 (3.7) | <0.001 |
| **Atrial fibrillation** | 1391 (3.7) | 265 (5.3) | <0.001 |
| **Coronary heart disease** | 4023 (10.7) | 624 (12.5) | <0.001 |
| **Systolic blood pressure, mmHg** | 141.2 (14.0) | 131.2 (17.8) | <0.001 |
| **Diastolic blood pressure, mmHg** | 81.3 (6.8) | 65.0 (10.2) | <0.001 |

Data are presented as No. (%) or mean (SD). ID, infectious disease; CKD, chronic kidney disease; CHD, coronary heart disease. * P values were obtained from either a chi-square test or a Mann-Whitney U test comparing difference between UKB and ARIC.

# Supplementary Table 7. Association between infectious diseases and cardiovascular measurements in the UK biobank (CMR sub study) and ARIC study (visit 5).

|  | **UK Biobank** | | | | | | | **ARIC** | | | | | | |
| --- | --- | --- | --- | --- | --- | --- | --- | --- | --- | --- | --- | --- | --- | --- |
|  | **Indicator** | **No infection** | **Infection** | **Infection vs no infection--Coefficient (95% CI)  for model 1** | **P Value  for model 1** | **Infection vs no infection--Coefficient (95% CI)  for model 2** | **P Value  for model 2** | **No infection** | **Infection** | **Infection vs no infection--Coefficient (95% CI)  for model 1** | **P Value  for model 1** | **Infection vs no infection--Coefficient (95% CI)  for model 2** | **P Value  for model 2** |  |
| **All infection** | LVSVI, mL/m2 | 46.61±8.47 | 42.89±8.3 | -3.9 (-4.2, -3.5) | <0.001 | -3.5 (-3.9, -3.1) | <0.001 |  |  |  |  |  |  |  |
|  | MWT, cm | 0.71±0.08 | 0.73±0.1 | 0.022 (0.018, 0.025) | <0.001 | 0.02 (0.016, 0.024) | <0.001 | 0.98±0.14 | 0.98±0.15 | 0.0056 (-0.0022, 0.013) | 0.1602 | 0.0059 (-0.0019, 0.014) | 0.1378 |  |
|  | LVMI, g/m2 | 44.99±8.77 | 46.1±8.93 | 1.2 (0.78, 1.5) | <0.001 | 1.1 (0.66, 1.5) | <0.001 | 36.63±9.66 | 38.29±10.34 | 1.7 (1.1, 2.2) | <0.001 | 1.6 (1.1, 2.2) | <0.001 |  |
|  | LVEDVI, mL/m2 | 77.52±12.71 | 72.92±13.26 | -4.8 (-5.4, -4.3) | <0.001 | -4.2 (-4.8, -3.6) | <0.001 | 47.21±13.53 | 43.85±14.08 | -3.4 (-4.1, -2.6) | <0.001 | -3.3 (-4.1, -2.6) | <0.001 |  |
|  | LVMVR, g/mL | 0.6±0.16 | 0.66±0.19 | 0.064 (0.057, 0.072) | <0.001 | 0.055 (0.047, 0.062) | <0.001 | 0.86±0.77 | 0.9±3.63 | 0.035 (-0.083, 0.15) | 0.56 | 0.029 (-0.089, 0.15) | 0.6339 |  |
|  | LVEF, % | 55.79±6.76 | 54.77±6.97 | -0.89 (-1.2, -0.61) | <0.001 | -1.1 (-1.4, -0.79) | <0.001 | 65.39±7.19 | 64.12±8.17 | -1.3 (-1.7, -0.87) | <0.001 | -1.3 (-1.7, -0.86) | <0.001 |  |
|  | LS, % | -17.8±3.33 | -17.1±3.76 | 0.74 (0.6, 0.89) | <0.001 | 0.67 (0.5, 0.83) | <0.001 | -18.05±3.31 | -17.89±2.87 | 0.16 (-0.012, 0.33) | 0.0694 | 0.17 (-0.0044, 0.34) | 0.0562 |  |
|  | CS, % | -24.8±3.74 | -23.3±4.65 | 1.6 (1.4, 1.7) | <0.001 | 1.4 (1.3, 1.6) | <0.001 | -27.57±3.91 | -27.17±4.07 | 0.4 (0.19, 0.61) | <0.001 | 0.39 (0.18, 0.6) | <0.001 |  |
|  | LAVI, mL/m2 | 24.09±8.78 | 24.23±9.23 | 0.21 (-0.17, 0.59) | 0.2735 | 0.12 (-0.3, 0.54) | 0.5685 | 24.84±7.91 | 24.72±7.85 | -0.12 (-0.54, 0.3) | 0.5808 | -0.15 (-0.58, 0.27) | 0.4809 |  |
|  | Septal e', cm/s |  |  |  |  |  |  | 5.76±1.5 | 5.78±1.45 | 0.026 (-0.053, 0.11) | 0.5158 | 0.025 (-0.055, 0.1) | 0.5423 |  |
|  | E/e' ratio |  |  |  |  |  |  | 12.22±3.84 | 12.83±4.23 | 0.6 (0.39, 0.82) | <0.001 | 0.59 (0.38, 0.81) | <0.001 |  |
| **Bacterial** | LVSVI, mL/m2 | 46.61±8.47 | 42.87±8.28 | -3.9 (-4.3, -3.5) | <0.001 | -3.6 (-4, -3.2) | <0.001 |  |  |  |  |  |  |  |
|  | MWT, cm | 0.71±0.08 | 0.73±0.1 | 0.022 (0.018, 0.026) | <0.001 | 0.02 (0.016, 0.024) | <0.001 | 0.98±0.14 | 0.98±0.15 | 0.0043 (-0.0036, 0.012) | 0.2881 | 0.0046 (-0.0034, 0.013) | 0.2585 |  |
|  | LVMI, g/m2 | 44.99±8.77 | 46.1±8.89 | 1.1 (0.73, 1.6) | <0.001 | 1.0 (0.55, 1.4) | <0.001 | 36.66±9.72 | 38.37±10.27 | 1.7 (1.2, 2.3) | <0.001 | 1.7 (1.2, 2.2) | <0.001 |  |
|  | LVEDVI, mL/m2 | 77.52±12.71 | 72.89±13.23 | -4.9 (-5.5, -4.3) | <0.001 | -4.4 (-5, -3.7) | <0.001 | 47.01±13.58 | 44.04±14.08 | -3 (-3.7, -2.2) | <0.001 | -3 (-3.7, -2.2) | <0.001 |  |
|  | LVMVR, g/mL | 0.6±0.16 | 0.66±0.19 | 0.065 (0.057, 0.073) | <0.001 | 0.056 (0.047, 0.064) | <0.001 | 0.86±0.91 | 0.9±3.7 | 0.04 (-0.08, 0.16) | 0.5182 | 0.034 (-0.086, 0.15) | 0.5799 |  |
|  | LVEF, % | 55.79±6.76 | 54.72±7.04 | -0.88 (-1.2, -0.58) | <0.001 | -1.1 (-1.5, -0.83) | <0.001 | 65.34±7.22 | 64.12±8.18 | -1.2 (-1.6, -0.81) | <0.001 | -1.2 (-1.6, -0.8) | <0.001 |  |
|  | LS, % | -17.8±3.33 | -17.1±3.74 | 0.73 (0.57, 0.88) | <0.001 | 0.66 (0.5, 0.83) | <0.001 | -18.03±3.29 | -17.9±2.87 | 0.13 (-0.042, 0.31) | 0.1362 | 0.14 (-0.035, 0.31) | 0.1166 |  |
|  | CS, % | -24.8±3.74 | -23.3±4.63 | 1.5 (1.3, 1.7) | <0.001 | 1.5 (1.3, 1.7) | <0.001 | -27.56±3.91 | -27.15±4.09 | 0.41 (0.19, 0.63) | <0.001 | 0.4 (0.19, 0.62) | <0.001 |  |
|  | LAVI, mL/m2 | 24.09±8.78 | 24.22±9.2 | 0.18 (-0.23, 0.59) | 0.3991 | 0.083 (-0.35, 0.51) | 0.7058 | 24.85±7.91 | 24.71±7.85 | -0.13 (-0.57, 0.3) | 0.5412 | -0.16 (-0.6, 0.27) | 0.4571 |  |
|  | Septal e', cm/s |  |  |  |  |  |  | 5.76±1.5 | 5.78±1.46 | 0.025 (-0.057, 0.11) | 0.5502 | 0.023 (-0.058, 0.1) | 0.5742 |  |
|  | E/e' ratio |  |  |  |  |  |  | 12.25±3.85 | 12.82±4.24 | 0.58 (0.36, 0.8) | <0.001 | 0.57 (0.35, 0.79) | <0.001 |  |
| **Viral** | LVSVI, mL/m2 | 46.61±8.47 | 43.01±8.24 | -3.7 (-4.9, -2.6) | <0.001 | -3.3 (-4.5, -2.2) | <0.001 |  |  |  |  |  |  |  |
|  | MWT, cm | 0.71±0.08 | 0.73±0.1 | 0.02 (0.0093, 0.031) | <0.001 | 0.024 (0.013, 0.034) | <0.001 | 0.98±0.14 | 0.98±0.16 | 0.0074 (-0.011, 0.025) | 0.4247 | 0.0078 (-0.01, 0.026) | 0.3980 |  |
|  | LVMI, g/m2 | 44.99±8.77 | 46.36±9.26 | 1.3 (0.18, 2.5) | 0.0239 | 1.3 (0.16, 2.5) | 0.0259 | 37.15±9.87 | 38.05±11.08 | 0.91 (-0.33, 2.1) | 0.1497 | 0.87 (-0.37, 2.1) | 0.1683 |  |
|  | LVEDVI, mL/m2 | 77.52±12.71 | 73.11±13.17 | -4.6 (-6.3, -2.9) | <0.001 | -4.0 (-5.7, -2.3) | <0.001 | 46.21±13.79 | 43.38±13.88 | -2.8 (-4.5, -1.1) | 0.0013 | -2.8 (-4.5, -1.1) | 0.0015 |  |
|  | LVMVR, g/mL | 0.6±0.16 | 0.66±0.19 | 0.067 (0.045, 0.088) | <0.001 | 0.06 (0.038, 0.081) | <0.001 | 0.87±2.2 | 0.88±2 | 0.01 (-0.26, 0.28) | 0.9408 | 0.0011 (-0.27, 0.27) | 0.9938 |  |
|  | LVEF, % | 55.79±6.76 | 54.28±7.45 | -0.93 (-1.8, -0.076) | 0.0328 | -1.8 (-2.7, -0.99) | <0.001 | 65±7.54 | 64.21±7.9 | -0.8 (-1.7, 0.14) | 0.0966 | -0.78 (-1.7, 0.16) | 0.1030 |  |
|  | LS, % | -17.8±3.33 | -16.99±3.94 | 0.79 (0.34, 1.2) | <0.001 | 0.76 (0.32, 1.2) | <0.001 | -18±3.18 | -17.83±2.97 | 0.17 (-0.22, 0.57) | 0.3861 | 0.19 (-0.21, 0.58) | 0.3570 |  |
|  | CS, % | -24.8±3.74 | -23.25±4.76 | 1.6 (1.1, 2.1) | <0.001 | 1.5 (1, 2) | <0.001 | -27.44±3.98 | -27.44±3.84 | -0.01 (-0.5, 0.48) | 0.9683 | -0.027 (-0.52, 0.47) | 0.9139 |  |
|  | LAVI, mL/m2 | 24.09±8.78 | 24.28±9.54 | 0.19 (-0.99, 1.4) | 0.7536 | 0.24 (-0.91, 1.4) | 0.6797 | 24.79±7.92 | 25.01±7.36 | 0.21 (-0.77, 1.2) | 0.6681 | 0.17 (-0.81, 1.2) | 0.7362 |  |
|  | Septal e', cm/s |  |  |  |  |  |  | 5.76±1.49 | 5.79±1.48 | 0.027 (-0.16, 0.21) | 0.7745 | 0.025 (-0.16, 0.21) | 0.7943 |  |
|  | E/e' ratio |  |  |  |  |  |  | 12.4±3.98 | 13.04±4.11 | 0.65 (0.15, 1.1) | 0.0104 | 0.63 (0.13, 1.1) | 0.0129 |  |
| **Fungal** | LVSVI, mL/m2 | 46.61±8.47 | 43.41±8.62 | -3.7 (-5, -2.4) | <0.001 | -2.6 (-3.7, -1.4) | <0.001 |  |  |  |  |  |  |  |
|  | MWT, cm | 0.71±0.08 | 0.73±0.1 | 0.03 (0.017, 0.043) | <0.001 | 0.0083 (-0.0026, 0.019) | 0.1348 | 0.98±0.14 | 0.96±0.16 | -0.015 (-0.034, 0.0038) | 0.1175 | -0.015 (-0.034, 0.0039) | 0.1197 |  |
|  | LVMI, g/m2 | 44.99±8.77 | 45.94±8.71 | 2.1 (0.72, 3.4) | 0.0028 | -0.15 (-1.3, 1) | 0.807 | 37.15±9.91 | 38.15±10.26 | 1 (-0.29, 2.3) | 0.1284 | 0.99 (-0.3, 2.3) | 0.1318 |  |
|  | LVEDVI, mL/m2 | 77.52±12.71 | 73.76±13.78 | -4.6 (-6.6, -2.6) | <0.001 | -2.7 (-4.4, -1) | 0.0016 | 46.21±13.72 | 43.14±15.56 | -3.1 (-4.9, -1.3) | <0.001 | -3.1 (-4.9, -1.3) | <0.001 |  |
|  | LVMVR, g/mL | 0.6±0.16 | 0.65±0.17 | 0.073 (0.048, 0.099) | <0.001 | 0.023 (0.0011, 0.045) | 0.0394 | 0.87±2.18 | 0.83±2.49 | -0.041 (-0.33, 0.24) | 0.7787 | -0.043 (-0.33, 0.24) | 0.7682 |  |
|  | LVEF, % | 55.79±6.76 | 55.15±6.07 | -1.3 (-2.3, -0.26) | 0.0136 | -0.18 (-1, 0.68) | 0.6854 | 65.01±7.53 | 63.82±8.04 | -1.2 (-2.2, -0.21) | 0.0177 | -1.2 (-2.2, -0.2) | 0.0181 |  |
|  | LS, % | -17.8±3.33 | -17.11±3.65 | 1.3 (0.73, 1.8) | <0.001 | 0.2 (-0.24, 0.65) | 0.3732 | -17.98±3.18 | -18.23±2.96 | -0.24 (-0.66, 0.17) | 0.2459 | -0.24 (-0.65, 0.17) | 0.2504 |  |
|  | CS, % | -24.8±3.74 | -23.35±4.57 | 2.1 (1.5, 2.7) | <0.001 | 0.88 (0.38, 1.4) | <0.001 | -27.45±3.96 | -27.04±4.22 | 0.42 (-0.1, 0.93) | 0.1152 | 0.41 (-0.11, 0.93) | 0.1185 |  |
|  | LAVI, mL/m2 | 24.09±8.78 | 24.43±8.95 | 1.9 (0.5, 3.3) | 0.0078 | -0.93 (-2.1, 0.25) | 0.1218 | 24.8±7.89 | 24.86±7.96 | 0.055 (-0.97, 1.1) | 0.9158 | 0.045 (-0.98, 1.1) | 0.9309 |  |
|  | Septal e', cm/s |  |  |  |  |  |  | 5.77±1.49 | 5.75±1.37 | -0.013 (-0.21, 0.18) | 0.8959 | -0.013 (-0.21, 0.18) | 0.8916 |  |
|  | E/e' ratio |  |  |  |  |  |  | 12.42±3.97 | 12.57±4.45 | 0.15 (-0.37, 0.67) | 0.5656 | 0.15 (-0.37, 0.67) | 0.5767 |  |
| **Parasitic** | LVSVI, mL/m2 | 46.61±8.47 | 42.2±8.68 | -4.8 (-6.4, -3.2) | <0.001 | -3.6 (-5.2, -1.9) | <0.001 |  |  |  |  |  |  |  |
|  | MWT, cm | 0.71±0.08 | 0.72±0.1 | 0.013 (-0.0026, 0.029) | 0.1018 | 0.013 (-0.003, 0.028) | 0.1139 | 0.98±0.15 | 0.98±0.14 | 0.00026 (-0.018, 0.019) | 0.9783 | 0.00015 (-0.019, 0.019) | 0.9871 |  |
|  | LVMI, g/m2 | 44.99±8.77 | 45.31±9.1 | -0.24 (-1.9, 1.4) | 0.7791 | 0.84 (-0.88, 2.6) | 0.3375 | 37.14±9.89 | 38.26±10.75 | 1.1 (-0.15, 2.4) | 0.0848 | 1.1 (-0.14, 2.4) | 0.0820 |  |
|  | LVEDVI, mL/m2 | 77.52±12.71 | 71.83±13.87 | -6.3 (-8.8, -3.9) | <0.001 | -3.4 (-5.8, -0.9) | <0.001 | 46.16±13.8 | 44.37±13.93 | -1.8 (-3.6, -0.016) | 0.048 | -1.8 (-3.6, -0.027) | 0.0466 |  |
|  | LVMVR, g/mL | 0.6±0.16 | 0.66±0.21 | 0.059 (0.028, 0.09) | <0.001 | 0.064 (0.032, 0.096) | <0.001 | 0.87±2.23 | 0.97±0.58 | 0.099 (-0.18, 0.38) | 0.4916 | 0.1 (-0.18, 0.38) | 0.4816 |  |
|  | LVEF, % | 55.79±6.76 | 54.54±7.26 | -1.1 (-2.3, 0.16) | 0.0894 | -1.5 (-2.7, -0.19) | 0.0239 | 65.01±7.53 | 64.01±8.15 | -0.99 (-2, -0.021) | 0.0452 | -1 (-2, -0.025) | 0.0443 |  |
|  | LS, % | -17.8±3.33 | -17.33±3.79 | 0.34 (-0.3, 0.98) | 0.3041 | 0.57 (-0.091, 1.2) | 0.0914 | -17.99±3.19 | -17.95±2.69 | 0.042 (-0.37, 0.45) | 0.84 | 0.039 (-0.37, 0.45) | 0.8500 |  |
|  | CS, % | -24.8±3.74 | -23.75±4.7 | 0.98 (0.25, 1.7) | 0.0084 | 1 (0.3, 1.8) | 0.0061 | -27.44±3.96 | -27.34±4.14 | 0.098 (-0.41, 0.61) | 0.7071 | 0.1 (-0.41, 0.61) | 0.6950 |  |
|  | LAVI, mL/m2 | 24.09±8.78 | 23.53±9.49 | -0.85 (-2.5, 0.84) | 0.3241 | -0.4 (-2.1, 1.3) | 0.6439 | 24.76±7.88 | 25.79±8.08 | 1 (0.018, 2) | 0.0461 | 1 (0.029, 2.1) | 0.0437 |  |
|  | Septal e', cm/s |  |  |  |  |  |  | 5.76±1.49 | 5.85±1.47 | 0.083 (-0.11, 0.27) | 0.3943 | 0.084 (-0.11, 0.27) | 0.3909 |  |
|  | E/e' ratio |  |  |  |  |  |  | 12.4±3.96 | 13±4.53 | 0.6 (0.085, 1.1) | 0.0224 | 0.6 (0.09, 1.1) | 0.0213 |  |

Model 1 was adjusted for sex and sex. Model 2 was adjusted for sex, age, level of education, smoking status, alcohol drinking, height, weight, hypertension, diabetes, chronic kidney disease and low-density lipoprotein cholesterol. LVSVI, left ventricular stroke volume index; MWT = mean wall thickness; LVMI = left ventricular mass index; LVEDVI, left ventricular end-diastolic volume index; LVMVR = left ventricular mass-to-volume ratio; LVEF = left ventricular ejection fraction; LS = longitudinal strain; CS = circumferential strain; LAVI = left atrial volume index.

# Supplementary Table 8. Baseline characteristics of participants included in analysis 5 in the UK Biobank (CMR sub-study) and ARIC study (visit 5).

|  | **UKB** | **ARIC** | **P for difference between two cohorts** |
| --- | --- | --- | --- |
| **No. of participants** | 30801 | 3242 |  |
| **Age** | 63.8 (7.3) | 76.3 (5.3) | <0.001 |
| **Women** | 15986 (51.9) | 2159 (66.6) | <0.001 |
| **More than high school** | 10195 (33.1) | 1015 (31.3) | <0.001 |
| **Current smoker** | 1848 (6.0) | 266 (8.2) | <0.001 |
| **Never drinker** | 1509 (4.9) | 600 (18.5) | <0.001 |
| **Height, cm** | 169.0 (9.3) | 165.4 (8.8) | <0.001 |
| **Weight, kg** | 75.8 (15.3) | 63.3 (9.1) | <0.001 |
| **Diabetes** | 1910 (6.2) | 577 (17.8) | <0.001 |
| **Hypertension** | 22546 (73.2) | 2321 (71.6) | <0.001 |
| **CKD** | 986 (3.2) | 81 (2.5) | <0.001 |
| **Atrial fibrillation** | 893 (2.9) | 120 (3.7) | <0.001 |
| **Coronary heart disease** | 2680 (8.7) | 347 (10.7) | <0.001 |
| **Systolic blood pressure, mmHg** | 141.0 (14.2) | 131.6 (18.0) | <0.001 |
| **Diastolic blood pressure, mmHg** | 80.9 (7.0) | 64.8 (10.4) | <0.001 |

Data are presented as No. (%) or mean (SD). ID, infectious disease; CKD, chronic kidney disease; CHD, coronary heart disease. * P values were obtained from either a chi-square test or a Mann-Whitney U test comparing difference between UKB and ARIC.

# Supplementary Table 9. Association between pathway-based PRS for infectious disease and cardiovascular measurements in the UK biobank (CMR sub study) and ARIC study (visit 5).

|  |  | **UKB** | | | | | **ARIC** | | | | |
| --- | --- | --- | --- | --- | --- | --- | --- | --- | --- | --- | --- |
|  |  | **Mean ± SD (Low)** | **Mean ± SD (Medium)** | **Mean ± SD (High)** | **Beta (95% CI) per 1-SD increase** | **P value** | **Mean ± SD (Low)** | **Mean ± SD (Medium)** | **Mean ± SD (High)** | **Beta (95% CI) per 1-SD increase** | **P value** |
| **Overall PRS** | LVSVI, mL/m2 | 46.5±17.8 | 46.6±18.3 | 45.7±12.0 | -0.042 (-0.66, 0.57) | 0.894 |  |  |  |  |  |
|  | MWT, cm | 0.71±0.08 | 0.71±0.08 | 0.72±0.08 | 0.0051 (0.0041, 0.006) | <0.001 | 0.99±0.15 | 0.97±0.15 | 0.98±0.15 | -0.0031 (-0.0081, 0.002) | 0.238 |
|  | LVMI, g/m2 | 44.4±8.9 | 44.9±8.5 | 45.7±8.9 | 0.58 (0.47, 0.68) | <0.001 | 37.4±10.0 | 37.4±10.0 | 38.0±9.9 | 0.27 (-0.068, 0.61) | 0.117 |
|  | LVEDVI, mL/m2 | 77.2±47.8 | 76.6±85.1 | 75.1±31.6 | -0.57 (-1.2, 0.11) | 0.1000 | 45.7±14.0 | 45.5±14.1 | 44.5±14.0 | -0.55 (-1, -0.067) | 0.026 |
|  | LVMVR, g/mL | 0.66±0.23 | 0.67±0.23 | 0.69±0.25 | 0.0082 (0.0056, 0.011) | <0.001 | 0.95±0.54 | 0.88±1.29 | 0.91±4.64 | -0.075 (-0.17, 0.023) | 0.135 |
|  | LVEF, % | 55.8±6.7 | 55.6±6.8 | 55.1±7.0 | -0.17 (-0.25, -0.094) | <0.001 | 64.6±7.8 | 64.3±7.7 | 63.6±8.0 | -0.38 (-0.65, -0.12) | 0.005 |
|  | LS, % | -18.0±3.4 | -17.8±3.2 | -17.5±3.4 | 0.22 (0.18, 0.25) | <0.001 | -17.7±3.1 | -18.1±3.1 | -18.0±3.0 | -0.059 (-0.16, 0.045) | 0.267 |
|  | CS, % | -25.0±3.8 | -24.8±3.6 | -24.5±3.8 | 0.24 (0.2, 0.28) | <0.001 | -27.2±4.1 | -26.9±3.9 | -26.5±4.0 | 0.18 (0.041, 0.31) | 0.011 |
|  | LAVI, mL/m2 | 24.0±8.9 | 24.0±8.5 | 24.0±8.9 | 0.03 (-0.071,0.14) | 0.112 | 24.4±7.6 | 24.7±7.8 | 24.9±7.9 | 0.23 (-0.037, 0.5) | 0.092 |
|  | Septal e', cm/s |  |  |  |  |  | 5.8±1.5 | 5.7±1.5 | 5.8±1.4 | -0.0011 (-0.051, 0.049) | 0.965 |
|  | E/e' ratio |  |  |  |  |  | 12.7±4.2 | 12.7±4.1 | 13.1±4.1 | 0.2 (0.055, 0.34) | 0.006 |
| **TGF-β** | LVSVI, mL/m2 | 45.4±14.2 | 46.1±13.8 | 47.4±89.0 | 0.22 (-0.38, 0.81) | 0.471 |  |  |  |  |  |
|  | MWT, cm | 0.71±0.08 | 0.71±0.08 | 0.71±0.08 | 0.0025 (0.0016, 0.0035) | <0.001 | 0.99±0.15 | 0.97±0.15 | 0.98±0.14 | -0.00018 (-0.0052, 0.0048) | 0.945 |
|  | LVMI, g/m2 | 44.6±8.7 | 45.1±8.7 | 45.3±8.9 | 0.31 (0.21, 0.41) | <0.001 | 37.0±10.2 | 37.7±9.9 | 38.0±9.8 | 0.36 (0.021, 0.69) | 0.037 |
|  | LVEDVI, mL/m2 | 77.5±86.0 | 76.0±32.3 | 75.6±47.3 | -0.2 (-0.85, 0.45) | 0.545 | 45.3±14.1 | 45.1±13.9 | 45.2±14.1 | -0.03 (-0.5, 0.44) | 0.900 |
|  | LVMVR, g/mL | 0.67±0.23 | 0.67±0.24 | 0.68±0.24 | 0.0026 (7.4e-05, 0.005) | 0.044 | 0.93±0.54 | 0.91±1.24 | 0.89±4.75 | -0.072 (-0.17, 0.025) | 0.143 |
|  | LVEF, % | 55.7±6.9 | 55.5±6.7 | 55.3±6.8 | -0.037 (-0.11, 0.039) | 0.340 | 64.2±7.6 | 64.2±7.8 | 64.0±8.1 | 0.067 (-0.2, 0.33) | 0.619 |
|  | LS, % | -17.9±3.3 | -17.8±3.3 | -17.7±3.4 | 0.11 (0.077, 0.15) | <0.001 | -17.8±3.1 | -18.1±3.2 | -17.9±2.9 | -0.0018 (-0.11, 0.1) | 0.973 |
|  | CS, % | -25.0±3.7 | -24.8±3.7 | -24.7±3.8 | 0.12 (0.08, 0.16) | <0.001 | -27.0±4.1 | -26.9±3.9 | -26.7±4.0 | 0.11 (-0.024, 0.25) | 0.108 |
|  | LAVI, mL/m2 | 23.9±8.7 | 24.2±8.7 | 24.0±8.9 | 0.094 (-0.0063, 0.19) | 0.066 | 24.5±7.7 | 24.9±7.8 | 24.7±7.9 | 0.17 (-0.098, 0.43) | 0.218 |
|  | Septal e', cm/s |  |  |  |  |  | 5.7±1.5 | 5.7±1.5 | 5.8±1.4 | 0.037 (-0.012, 0.087) | 0.141 |
|  | E/e' ratio |  |  |  |  |  | 12.7±4.0 | 12.7±4.1 | 13.0±4.2 | 0.15 (0.0097, 0.29) | 0.036 |
| **Myocardial fibrosis** | LVSVI, mL/m2 | 47.2±90.0 | 45.9±13.1 | 45.9±12.0 | -0.38 (-0.97, 0.21) | 0.207 |  |  |  |  |  |
|  | MWT, cm | 0.71±0.08 | 0.71±0.08 | 0.71±0.08 | 0.00062 (-0.00029, 0.0015) | 0.179 | 0.98±0.15 | 0.98±0.15 | 0.98±0.15 | 0.00044 (-0.0046, 0.0054) | 0.863 |
|  | LVMI, g/m2 | 44.8±8.7 | 45.1±8.8 | 45.0±8.9 | 0.092 (-0.007, 0.19) | 0.069 | 37.7±10.1 | 37.5±9.9 | 37.6±9.9 | 0.024 (-0.31, 0.36) | 0.887 |
|  | LVEDVI, mL/m2 | 77.3±94.0 | 76.0±32.3 | 75.8±29.7 | -0.42 (-1.1, 0.22) | 0.198 | 44.9±14.3 | 45.6±14.1 | 45.1±13.8 | -0.067 (-0.54, 0.41) | 0.780 |
|  | LVMVR, g/mL | 0.67±0.23 | 0.68±0.23 | 0.68±0.24 | 0.0027 (0.00026, 0.0052) | 0.030 | 0.99±0.62 | 0.91±1.28 | 0.85±4.72 | -0.027 (-0.12, 0.07) | 0.580 |
|  | LVEF, % | 55.5±6.7 | 55.5±6.9 | 55.6±6.8 | -0.04 (-0.11, 0.035) | 0.293 | 64.3±7.6 | 63.9±8.0 | 64.2±7.9 | -0.17 (-0.43, 0.095) | 0.210 |
|  | LS, % | -17.9±3.3 | -17.7±3.3 | -17.8±3.4 | 0.027 (-0.011, 0.064) | 0.165 | -18.0±3.1 | -18.0±3.0 | -17.9±3.1 | 0.013 (-0.09, 0.12) | 0.805 |
|  | CS, % | -24.9±3.7 | -24.7±3.7 | -24.8±3.8 | 0.026 (-0.016, 0.068) | 0.228 | -26.8±4.1 | -26.7±3.9 | -27.0±4.0 | -0.12 (-0.26, 0.0099) | 0.069 |
|  | LAVI, mL/m2 | 23.9±8.7 | 24.2±8.8 | 24.0±8.9 | 0.063 (-0.036, 0.16) | 0.212 | 24.7±8.0 | 24.8±7.6 | 24.6±7.8 | 0.14 (-0.13, 0.4) | 0.311 |
|  | Septal e', cm/s |  |  |  |  |  | 5.7±1.5 | 5.8±1.5 | 5.8±1.5 | 0.046 (-0.0034, 0.096) | 0.068 |
|  | E/e' ratio |  |  |  |  |  | 12.7±4.0 | 12.9±4.3 | 12.9±4.0 | 0.1 (-0.035, 0.24) | 0.145 |
| **Acute inflammation** | LVSVI, mL/m2 | 46.6±16.9 | 46.7±88.3 | 45.6±14.5 | -0.027 (-0.63, 0.58) | 0.929 |  |  |  |  |  |
|  | MWT, cm | 0.71±0.08 | 0.71±0.08 | 0.71±0.08 | 0.003 (0.0021, 0.0039) | <0.001 | 0.98±0.15 | 0.98±0.16 | 0.98±0.14 | -0.001 (-0.0063, 0.0043) | 0.704 |
|  | LVMI, g/m2 | 44.7±8.8 | 44.9±8.6 | 45.4±8.9 | 0.31 (0.21, 0.41) | <0.001 | 37.9±9.9 | 37.1±9.9 | 37.8±10.1 | -0.0062 (-0.36, 0.35) | 0.973 |
|  | LVEDVI, mL/m2 | 77.2±41.0 | 76.6±83.2 | 75.2±45.1 | -0.14 (-0.8, 0.52) | 0.677 | 44.7±14.2 | 45.6±14.2 | 45.3±13.7 | 0.087 (-0.41, 0.59) | 0.734 |
|  | LVMVR, g/mL | 0.66±0.23 | 0.67±0.23 | 0.69±0.25 | 0.0055 (0.003, 0.008) | <0.001 | 0.84±4.83 | 0.91±1.12 | 1.00±0.69 | 0.067 (-0.035, 0.17) | 0.199 |
|  | LVEF, % | 55.6±6.7 | 55.6±6.8 | 55.4±6.8 | -0.14 (-0.22, -0.062) | <0.001 | 64.6±8.1 | 64.2±7.7 | 63.6±7.6 | -0.29 (-0.57, -0.011) | 0.042 |
|  | LS, % | -17.9±3.4 | -17.8±3.3 | -17.6±3.4 | 0.12 (0.086, 0.16) | <0.001 | -17.9±3.1 | -18.0±3.2 | -18.0±2.8 | -0.024 (-0.13, 0.085) | 0.660 |
|  | CS, % | -24.9±3.8 | -24.8±3.7 | -24.6±3.8 | 0.14 (0.1, 0.19) | <0.001 | -27.0±4.1 | -26.8±4.0 | -26.7±3.9 | 0.13 (-0.016, 0.27) | 0.082 |
|  | LAVI, mL/m2 | 23.9±8.8 | 24.0±8.6 | 24.1±8.9 | 0.05 (-0.0468-0.16) | 0.143 | 24.9±7.8 | 24.7±7.8 | 24.5±7.9 | -0.22 (-0.49, 0.061) | 0.126 |
|  | Septal e', cm/s |  |  |  |  |  | 5.8±1.5 | 5.7±1.5 | 5.7±1.5 | -0.018 (-0.071, 0.034) | 0.491 |
|  | E/e' ratio |  |  |  |  |  | 12.6±4.1 | 12.8±4.2 | 13.1±4.1 | 0.18 (0.034, 0.33) | 0.016 |

Model was adjusted for sex, age, level of education, smoking status, alcohol drinking, height, weight, hypertension, diabetes, chronic kidney disease and low-density lipoprotein cholesterol. LVSVI, left ventricular stroke volume index; MWT = mean wall thickness; LVMI = left ventricular mass index; LVEDVI, left ventricular end-diastolic volume index; LVMVR = left ventricular mass-to-volume ratio; LVEF = left ventricular ejection fraction; LS = longitudinal strain; CS = circumferential strain; LAVI = left atrial volume index


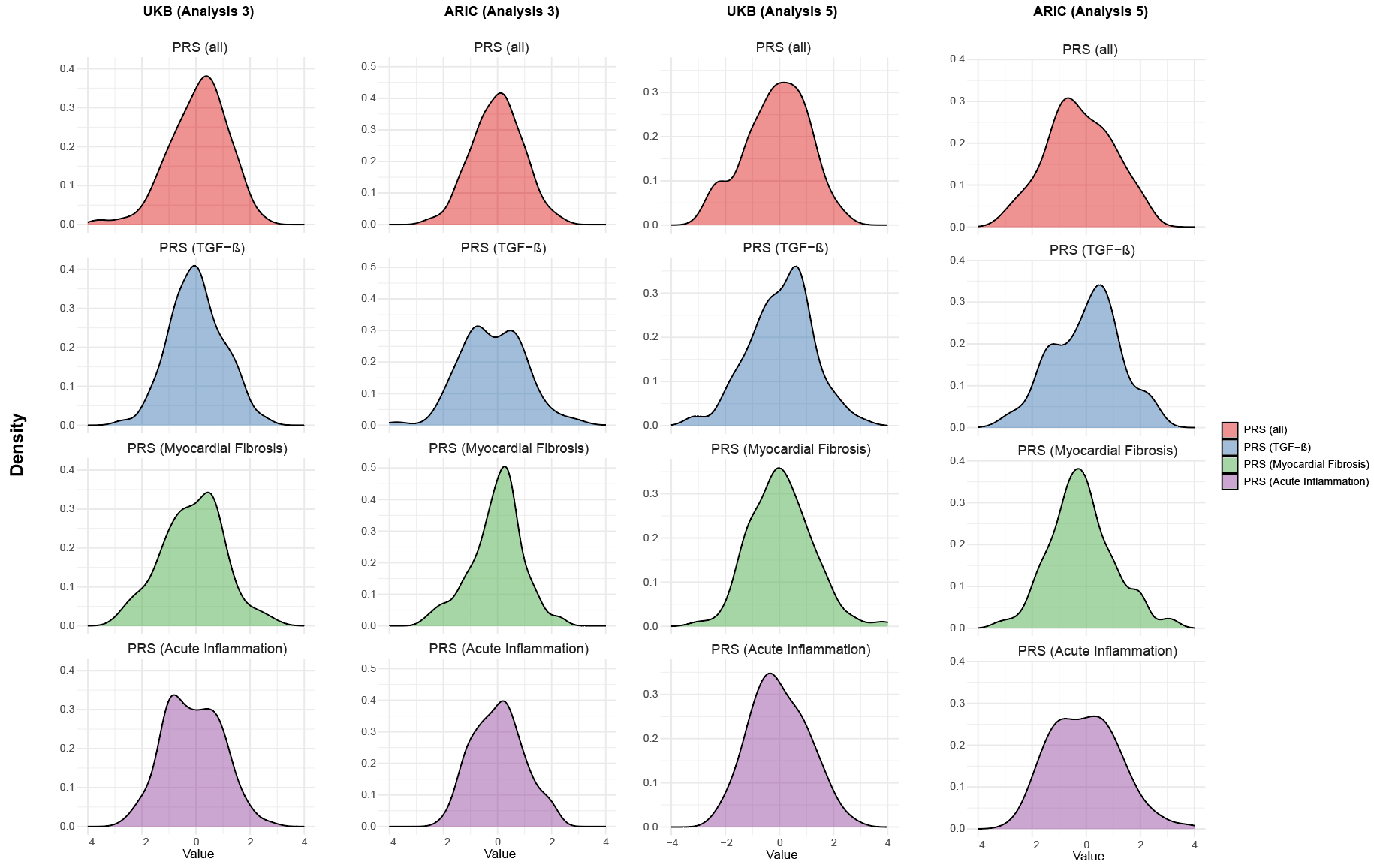


# Supplementary Figure 1. Distribution density plots of pathway-based polygenetic risk score in UK Biobank and Atherosclerosis Risk in Communities (ARIC) study across different stages of analysis


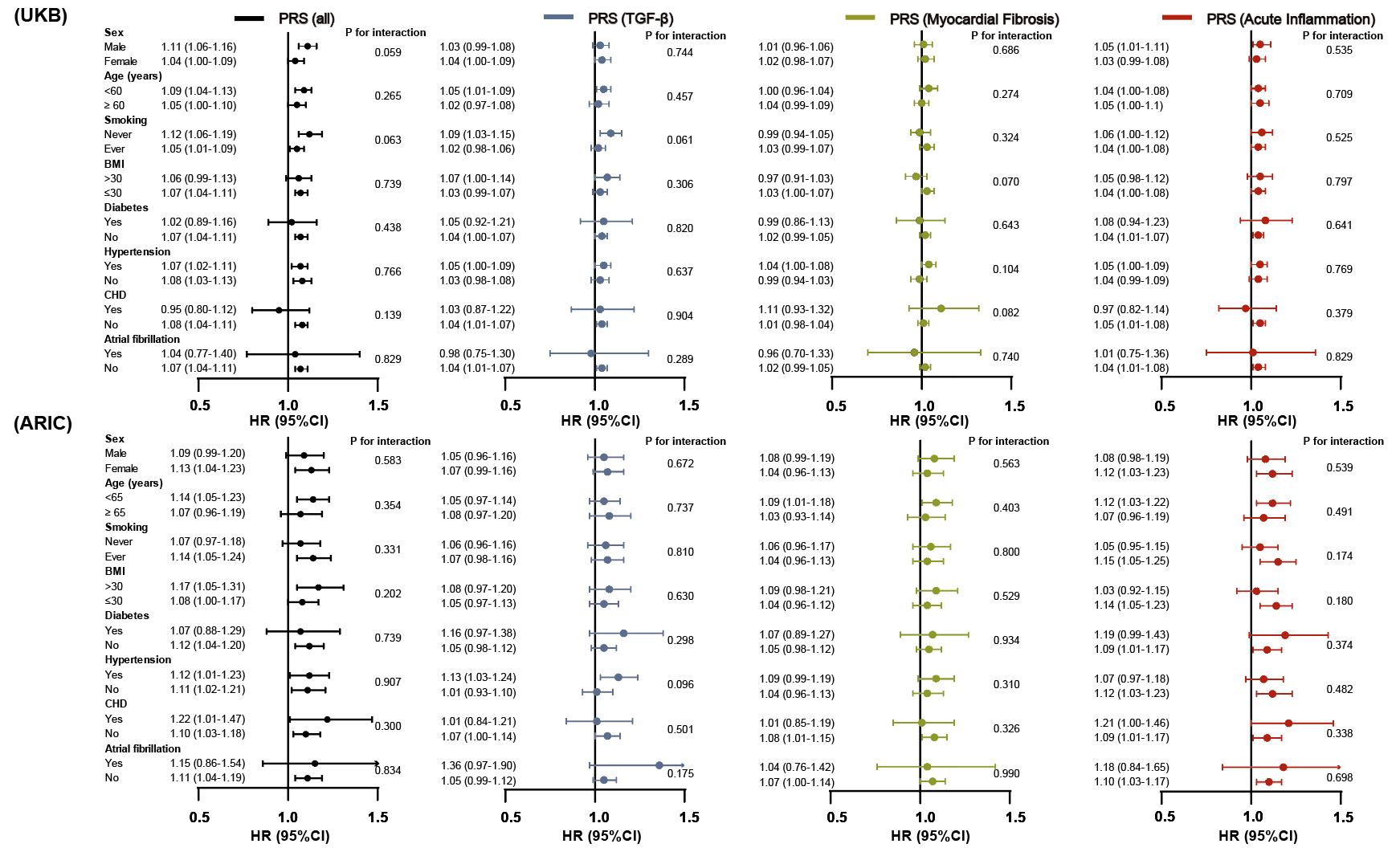
Supplementary Figure 2. Subgroup analyses of infectious diseases and risk of heart failure in the UK biobank and ARIC study.

Cox proportional hazards models were adjusted for sex, age (time scale), level of education, smoking status, alcohol drinking, height, weight, hypertension, diabetes, chronic kidney disease and low-density lipoprotein cholesterol. HR = hazard ratio; BMI = body mass index; CHD = coronary heart disease.

**Supplementary Table 10.** Classification of type-specific hospital-treated infectious diseases in the study on diagnosis level

**All infectious diseases**

A00 Cholera

A00.0 Cholera due to Vibrio cholerae 01, biovar cholerae

A00.1 Cholera due to Vibrio cholerae 01, biovar eltor

A00.9 Cholera, unspecified

A01* Typhoid and paratyphoid fevers

A02 Other salmonella infections

A02.0 Salmonella enteritis

A02.1 Salmonella sepsis

A02.2 Localized salmonella infections

A02.8 Other specified salmonella infections

A02.9 Salmonella infection, unspecified

A03 Shigellosis

A03.0 Shigellosis due to Shigella dysenteriae

A03.1 Shigellosis due to Shigella flexneri

A03.2 Shigellosis due to Shigella boydii

A03.3 Shigellosis due to Shigella sonnei

A03.8 Other shigellosis

A03.9 Shigellosis, unspecified

A04 Other bacterial intestinal infections

A04.0 Enteropathogenic Escherichia coli infection

A04.1 Enterotoxigenic Escherichia coli infection

A04.2 Enteroinvasive Escherichia coli infection

A04.3 Enterohaemorrhagic Escherichia coli infection

A04.4 Other intestinal Escherichia coli infections

A04.5 Campylobacter enteritis

A04.6 Enteritis due to Yersinia enterocolitica

A04.7 Enterocolitis due to Clostridium difficile

A04.8 Other specified bacterial intestinal infections

A04.9 Bacterial intestinal infection, unspecified

A05 Other bacterial foodborne intoxications, not elsewhere classified

A05.0 Foodborne staphylococcal intoxication

A05.1 Botulism

A05.2 Foodborne Clostridium perfringens [Clostridium welchii] intoxication

A05.3 Foodborne Vibrio parahaemolyticus intoxication

A05.4 Foodborne Bacillus cereus intoxication

A05.8 Other specified bacterial foodborne intoxications

A05.9 Bacterial foodborne intoxication, unspecified

A06 Amoebiasis

A06.0 Acute amoebic dysentery

A06.1 Chronic intestinal amoebiasis

A06.2 Amoebic nondysenteric colitis

A06.3 Amoeboma of intestine

A06.4 Amoebic liver abscess

A06.5 Amoebic lung abscess

A06.6 Amoebic brain abscess

A06.7 Cutaneous amoebiasis

A06.8 Amoebic infection of other sites

A06.9 Amoebiasis, unspecified

A07* Other protozoal intestinal diseases

A08.0 Rotaviral enteritis

A08.1 Acute gastroenteropathy due to Norwalk agent

A08.2 Adenoviral enteritis

A08.3 Other viral enteritis

A08.4 Viral intestinal infection, unspecified

A08.5 Other specified intestinal infections

A09* Other gastroenteritis and colitis of infectious and unspecified origin

A15* Respiratory tuberculosis, bacteriologically and histologically confirmed

A16* Respiratory tuberculosis, not confirmed bacteriologically or histologically

A17 Tuberculosis of nervous system

A17.0 Tuberculous meningitis

A17.1 Meningeal tuberculoma

A17.8 Other tuberculosis of nervous system

A17.9 Tuberculosis of nervous system, unspecified

A18* Tuberculosis of other organs

A19* Miliary tuberculosis

A20 Plague

A20.0 Bubonic plague

A20.1 Cellulocutaneous plague

A20.2 Pneumonic plague

A20.3 Plague meningitis

A20.7 Septicaemic plague

A20.8 Other forms of plague

A20.9 Plague, unspecified

A21 Tularaemia

A21.0 Ulceroglandular tularaemia

A21.1 Oculoglandular tularaemia

A21.2 Pulmonary tularaemia

A21.3 Gastrointestinal tularaemia

A21.7 Generalized tularaemia

A21.8 Other forms of tularaemia

A21.9 Tularaemia, unspecified

A22 Anthrax

A22.0 Cutaneous anthrax

A22.1 Pulmonary anthrax

A22.2 Gastrointestinal anthrax

A22.7 Anthrax sepsis

A22.8 Other forms of anthrax

A22.9 Anthrax, unspecified

A23 Brucellosis

A23.0 Brucellosis due to Brucella melitensis

A23.1 Brucellosis due to Brucella abortus

A23.2 Brucellosis due to Brucella suis

A23.3 Brucellosis due to Brucella canis

A23.8 Other brucellosis

A23.9 Brucellosis, unspecified

A24 Glanders and melioidosis

A24.0 Glanders

A24.1 Acute and fulminating melioidosis

A24.2 Subacute and chronic melioidosis

A24.3 Other melioidosis

A25 Rat-bite fevers

A25.0 Spirillosis

A25.1 Streptobacillosis

A25.9 Rat-bite fever, unspecified

A26 Erysipeloid

A26.0 Cutaneous erysipeloid

A26.7 Erysipelothrix sepsis

A26.8 Other forms of erysipeloid

A26.9 Erysipeloid, unspecified

A27* Leptospirosis

A28 Other zoonotic bacterial diseases, not elsewhere classified

A28.0 Pasteurellosis

A28.1 Cat-scratch disease

A28.2 Extraintestinal yersiniosis

A28.8 Other specified zoonotic bacterial diseases, not elsewhere classified

A28.9 Zoonotic bacterial disease, unspecified

A30* Leprosy [Hansen disease]

A31* Infection due to other mycobacteria

A32 Listeriosis

A32.0 Cutaneous listeriosis

A32.1 Listerial meningitis and meningoencephalitis

A32.7 Listerial sepsis

A32.8 Other forms of listeriosis

A32.9 Listeriosis, unspecified

A33* Tetanus neonatorum

A34* Obstetrical tetanus

A35* Other tetanus

A36* Diphtheria

A37* Whooping cough

A38* Scarlet fever

A39 Meningococcal infection

A39.0 Meningococcal meningitis

A39.1 Waterhouse-Friderichsen syndrome

A39.2 Acute meningococcaemia

A39.3 Chronic meningococcaemia

A39.4 Meningococcaemia, unspecified

A39.5 Meningococcal heart disease

A39.8 Other meningococcal infections

A39.9 Meningococcal infection, unspecified

A40 Streptococcal sepsis

A40.0 Sepsis due to streptococcus, group A

A40.1 Sepsis due to streptococcus, group B

A40.2 Sepsis due to streptococcus, group D

A40.3 Sepsis due to Streptococcus pneumoniae

A40.8 Other streptococcal sepsis

A40.9 Streptococcal sepsis, unspecified

A41 Other sepsis

A41.0 Sepsis due to Staphylococcus aureus

A41.1 Sepsis due to other specified staphylococcus

A41.2 Sepsis due to unspecified staphylococcus

A41.3 Sepsis due to Haemophilus influenzae

A41.4 Sepsis due to anaerobes

A41.5 Sepsis due to other Gram-negative organisms

A41.8 Other specified sepsis

A41.9 Sepsis, unspecified

A42 Actinomycosis

A42.0 Pulmonary actinomycosis

A42.1 Abdominal actinomycosis

A42.2 Cervicofacial actinomycosis

A42.7 Actinomycotic sepsis

A42.8 Other forms of actinomycosis

A42.9 Actinomycosis, unspecified

A43 Nocardiosis

A43.0 Pulmonary nocardiosis

A43.1 Cutaneous nocardiosis

A43.8 Other forms of nocardiosis

A44 Bartonellosis

A44.0 Systemic bartonellosis

A44.1 Cutaneous and mucocutaneous bartonellosis

A44.8 Other forms of bartonellosis

A44.9 Bartonellosis, unspecified

A46* Erysipelas

A48.0 Gas gangrene

A48.1 Legionnaires disease

A48.2 Nonpneumonic Legionnaires disease [Pontiac fever]

A48.3 Toxic shock syndrome

A48.4 Brazilian purpuric fever

A48.8 Other specified bacterial diseases

A49 Bacterial infection of unspecified site

A49.0 Staphylococcal infection, unspecified site

A49.1 Streptococcal infection, unspecified site

A49.2 Haemophilus influenzae infection, unspecified site

A49.3 Mycoplasma infection, unspecified site

A49.8 Other bacterial infections of unspecified site

A49.9 Bacterial infection, unspecified

A50 Congenital syphilis

A50.0 Early congenital syphilis, symptomatic

A50.1 Early congenital syphilis, latent

A50.2 Early congenital syphilis, unspecified

A50.3 Late congenital syphilitic oculopathy

A50.4 Late congenital neurosyphilis [juvenile neurosyphilis]

A50.5 Other late congenital syphilis, symptomatic

A50.6 Late congenital syphilis, latent

A50.7 Late congenital syphilis, unspecified

A50.9 Congenital syphilis, unspecified

A51 Early syphilis

A51.0 Primary genital syphilis

A51.1 Primary anal syphilis

A51.2 Primary syphilis of other sites

A51.3 Secondary syphilis of skin and mucous membranes

A51.4 Other secondary syphilis

A51.5 Early syphilis, latent

A51.9 Early syphilis, unspecified

A52 Late syphilis

A52.0 Cardiovascular syphilis

A52.1 Symptomatic neurosyphilis

A52.2 Asymptomatic neurosyphilis

A52.3 Neurosyphilis, unspecified

A52.7 Other symptomatic late syphilis

A52.8 Late syphilis, latent

A52.9 Late syphilis, unspecified

A53* Other and unspecified syphilis

A54 Gonococcal infection

A54.0 Gonococcal infection of lower genitourinary tract without periurethral or accessory gland abscess

A54.1 Gonococcal infection of lower genitourinary tract with periurethral and accessory gland abscess

A54.2 Gonococcal pelviperitonitis and other gonococcal genitourinary infections

A54.3 Gonococcal infection of eye

A54.4 Gonococcal infection of musculoskeletal system

A54.5 Gonococcal pharyngitis

A54.6 Gonococcal infection of anus and rectum

A54.8 Other gonococcal infections

A54.9 Gonococcal infection, unspecified

A55* Chlamydial lymphogranuloma (venereum)

A56.0 Chlamydial infection of lower genitourinary tract

A56.1 Chlamydial infection of pelviperitoneum and other genitourinary organs

A56.2 Chlamydial infection of genitourinary tract, unspecified

A56.3 Chlamydial infection of anus and rectum

A56.4 Chlamydial infection of pharynx

A56.8 Sexually transmitted chlamydial infection of other sites

A57* Chancroid

A58* Granuloma inguinale

A59* Trichomoniasis

A60* Anogenital herpesviral [herpes simplex] infection

A63.0 Anogenital (venereal) warts

A63.8 Other specified predominantly sexually transmitted diseases

A64* Unspecified sexually transmitted disease

A65* Nonvenereal syphilis

A66* Yaws

A67* Pinta [carate]

A68* Relapsing fevers

A69 Other spirochaetal infections

A69.0 Necrotizing ulcerative stomatitis

A69.1 Other Vincent infections

A69.2 Lyme disease

A69.8 Other specified spirochaetal infections

A69.9 Spirochaetal infection, unspecified

A70* Chlamydia psittaci infection

A71* Trachoma

A74.0 Chlamydial conjunctivitis

A74.8 Other chlamydial diseases

A74.9 Chlamydial infection, unspecified

A75 Typhus fever

A75.0 Epidemic louse-borne typhus fever due to Rickettsia prowazekii

A75.1 Recrudescent typhus [Brill disease]

A75.2 Typhus fever due to Rickettsia typhi

A75.3 Typhus fever due to Rickettsia tsutsugamushi

A75.9 Typhus fever, unspecified

A77 Spotted fever [tick-borne rickettsioses]

A77.0 Spotted fever due to Rickettsia rickettsii

A77.1 Spotted fever due to Rickettsia conorii

A77.2 Spotted fever due to Rickettsia sibirica

A77.3 Spotted fever due to Rickettsia australis

A77.8 Other spotted fevers

A77.9 Spotted fever, unspecified

A78* Q fever

A79 Other rickettsioses

A79.0 Trench fever

A79.1 Rickettsialpox due to Rickettsia akari

A79.8 Other specified rickettsioses

A79.9 Rickettsiosis, unspecified

A80* Acute poliomyelitis

A81.1 Subacute sclerosing panencephalitis

A81.2 Progressive multifocal leukoencephalopathy

A83* Mosquito-borne viral encephalitis

A84* Tick-borne viral encephalitis

A85* Other viral encephalitis, not elsewhere classified

A86* Unspecified viral encephalitis

A87* Viral meningitis

A88* Other viral infections of central nervous system, not elsewhere classified

A89* Unspecified viral infection of central nervous system

A90* Dengue fever [classical dengue]

A91* Dengue haemorrhagic fever

A92 Other mosquito-borne viral fevers

A92.0 Chikungunya virus disease

A92.1 O'nyong-nyong fever

A92.2 Venezuelan equine fever

A92.3 West Nile virus infection

A92.4 Rift Valley fever

A92.8 Other specified mosquito-borne viral fevers

A92.9 Mosquito-borne viral fever, unspecified

A93 Other arthropod-borne viral fevers, not elsewhere classified

A93.0 Oropouche virus disease

A93.1 Sandfly fever

A93.2 Colorado tick fever

A93.8 Other specified arthropod-borne viral fevers

A94* Unspecified arthropod-borne viral fever

A95* Yellow fever

A96* Arenaviral haemorrhagic fever

A97* Dengue

A98 Other viral haemorrhagic fevers, not elsewhere classified

A98.0 Crimean-Congo haemorrhagic fever

A98.1 Omsk haemorrhagic fever

A98.2 Kyasanur Forest disease

A98.3 Marburg virus disease

A98.4 Ebola virus disease

A98.5 Haemorrhagic fever with renal syndrome

A98.8 Other specified viral haemorrhagic fevers

A99* Unspecified viral haemorrhagic fever

B00 Herpesviral [herpes simplex] infections

B00.0 Eczema herpeticum

B00.1 Herpesviral vesicular dermatitis

B00.2 Herpesviral gingivostomatitis and pharyngotonsillitis

B00.3 Herpesviral meningitis

B00.4 Herpesviral encephalitis

B00.5 Herpesviral ocular disease

B00.7 Disseminated herpesviral disease

B00.8 Other forms of herpesviral infection

B00.9 Herpesviral infection, unspecified

B01 Varicella [chickenpox]

B01.0 Varicella meningitis

B01.1 Varicella encephalitis

B01.2 Varicella pneumonia

B01.8 Varicella with other complications

B01.9 Varicella without complication

B02 Zoster [herpes zoster]

B02.0 Zoster encephalitis

B02.1 Zoster meningitis

B02.2 Zoster with other nervous system involvement

B02.3 Zoster ocular disease

B02.7 Disseminated zoster

B02.8 Zoster with other complications

B02.9 Zoster without complication

B04* Monkeypox

B05 Measles

B05.0 Measles complicated by encephalitis

B05.1 Measles complicated by meningitis

B05.2 Measles complicated by pneumonia

B05.3 Measles complicated by otitis media

B05.4 Measles with intestinal complications

B05.8 Measles with other complications

B05.9 Measles without complication

B06 Rubella [German measles]

B06.0 Rubella with neurological complications

B06.8 Rubella with other complications

B06.9 Rubella without complication

B07* Viral warts

B08.0 Other orthopoxvirus infections

B08.1 Molluscum contagiosum

B08.2 Exanthema subitum [sixth disease]

B08.3 Erythema infectiosum [fifth disease]

B08.4 Enteroviral vesicular stomatitis with exanthem

B08.5 Enteroviral vesicular pharyngitis

B08.8 Other specified viral infections characterized by skin and mucous membrane lesions

B09* Unspecified viral infection characterized by skin and mucous membrane lesions

B15 Acute hepatitis A

B15.0 Hepatitis A with hepatic coma

B15.9 Hepatitis A without hepatic coma

B16 Acute hepatitis B

B16.0 Acute hepatitis B with delta-agent (coinfection) with hepatic coma

B16.1 Acute hepatitis B with delta-agent (coinfection) without hepatic coma

B16.2 Acute hepatitis B without delta-agent with hepatic coma

B16.9 Acute hepatitis B without delta-agent and without hepatic coma

B17* Other acute viral hepatitis

B18* Chronic viral hepatitis

B19 Unspecified viral hepatitis

B19.0 Unspecified viral hepatitis with hepatic coma

B19.9 Unspecified viral hepatitis without hepatic coma

B20* Human immunodeficiency virus [HIV] disease resulting in infectious and parasitic diseases

B21* Human immunodeficiency virus [HIV] disease resulting in malignant neoplasms

B21.0 HIV disease resulting in Kaposi sarcoma

B22 Human immunodeficiency virus [HIV] disease resulting in other specified diseases

B22.0 HIV disease resulting in encephalopathy

B22.1 HIV disease resulting in lymphoid interstitial pneumonitis

B22.2 HIV disease resulting in wasting syndrome

B22.7 HIV disease resulting in multiple diseases classified elsewhere

B23* Human immunodeficiency virus [HIV] disease resulting in other conditions

B24* Unspecified human immunodeficiency virus [HIV] disease

B25* Cytomegaloviral disease

B26 Mumps

B26.0 Mumps orchitis

B26.1 Mumps meningitis

B26.2 Mumps encephalitis

B26.3 Mumps pancreatitis

B26.8 Mumps with other complications

B26.9 Mumps without complication

B27 Infectious mononucleosis

B27.0 Gammaherpesviral mononucleosis

B27.1 Cytomegaloviral mononucleosis

B27.8 Other infectious mononucleosis

B27.9 Infectious mononucleosis, unspecified

B30* Viral conjunctivitis

B33 Other viral diseases, not elsewhere classified

B33.0 Epidemic myalgia

B33.1 Ross River disease

B33.2 Viral carditis

B33.3 Retrovirus infections, not elsewhere classified

B33.4 Hantavirus (cardio-)pulmonary syndrome [HPS] [HCPS]

B33.8 Other specified viral diseases

B34 Viral infection of unspecified site

B34.0 Adenovirus infection, unspecified site

B34.1 Enterovirus infection, unspecified site

B34.2 Coronavirus infection, unspecified site

B34.3 Parvovirus infection, unspecified site

B34.4 Papovavirus infection, unspecified site

B34.8 Other viral infections of unspecified site

B34.9 Viral infection, unspecified

B35* Dermatophytosis

B36* Other superficial mycoses

B37 Candidiasis

B37.0 Candidal stomatitis

B37.1 Pulmonary candidiasis

B37.2 Candidiasis of skin and nail

B37.3 Candidiasis of vulva and vagina

B37.4 Candidiasis of other urogenital sites

B37.5 Candidal meningitis

B37.6 Candidal endocarditis

B37.7 Candidal sepsis

B37.8 Candidiasis of other sites

B37.9 Candidiasis, unspecified

B38 Coccidioidomycosis

B38.0 Acute pulmonary coccidioidomycosis

B38.1 Chronic pulmonary coccidioidomycosis

B38.2 Pulmonary coccidioidomycosis, unspecified

B38.3 Cutaneous coccidioidomycosis

B38.4 Coccidioidomycosis meningitis

B38.7 Disseminated coccidioidomycosis

B38.8 Other forms of coccidioidomycosis

B38.9 Coccidioidomycosis, unspecified

B39 Histoplasmosis

B39.0 Acute pulmonary histoplasmosis capsulati

B39.1 Chronic pulmonary histoplasmosis capsulati

B39.2 Pulmonary histoplasmosis capsulati, unspecified

B39.3 Disseminated histoplasmosis capsulati

B39.4 Histoplasmosis capsulati, unspecified

B39.5 Histoplasmosis duboisii

B39.9 Histoplasmosis, unspecified

B40 Blastomycosis

B40.0 Acute pulmonary blastomycosis

B40.1 Chronic pulmonary blastomycosis

B40.2 Pulmonary blastomycosis, unspecified

B40.3 Cutaneous blastomycosis

B40.7 Disseminated blastomycosis

B40.8 Other forms of blastomycosis

B40.9 Blastomycosis, unspecified

B41 Paracoccidioidomycosis

B41.0 Pulmonary paracoccidioidomycosis

B41.7 Disseminated paracoccidioidomycosis

B41.8 Other forms of paracoccidioidomycosis

B41.9 Paracoccidioidomycosis, unspecified

B42 Sporotrichosis

B42.0 Pulmonary sporotrichosis

B42.1 Lymphocutaneous sporotrichosis

B42.7 Disseminated sporotrichosis

B42.8 Other forms of sporotrichosis

B42.9 Sporotrichosis, unspecified

B43 Chromomycosis and phaeomycotic abscess

B43.0 Cutaneous chromomycosis

B43.1 Phaeomycotic brain abscess

B43.2 Subcutaneous phaeomycotic abscess and cyst

B43.8 Other forms of chromomycosis

B43.9 Chromomycosis, unspecified

B44* Aspergillosis

B45 Cryptococcosis

B45.0 Pulmonary cryptococcosis

B45.1 Cerebral cryptococcosis

B45.2 Cutaneous cryptococcosis

B45.3 Osseous cryptococcosis

B45.7 Disseminated cryptococcosis

B45.8 Other forms of cryptococcosis

B45.9 Cryptococcosis, unspecified

B46 Zygomycosis

B46.0 Pulmonary mucormycosis

B46.1 Rhinocerebral mucormycosis

B46.2 Gastrointestinal mucormycosis

B46.3 Cutaneous mucormycosis

B46.4 Disseminated mucormycosis

B46.5 Mucormycosis, unspecified

B46.8 Other zygomycoses

B46.9 Zygomycosis, unspecified

B47 Mycetoma

B47.0 Eumycetoma

B47.1 Actinomycetoma

B47.9 Mycetoma, unspecified

B48 Other mycoses, not elsewhere classified

B48.0 Lobomycosis

B48.1 Rhinosporidiosis

B48.2 Allescheriasis

B48.3 Geotrichosis

B48.4 Penicillosis

B48.7 Opportunistic mycoses

B48.8 Other specified mycoses

B49* Unspecified mycosis

B50* Plasmodium falciparum malaria

B50.0 Plasmodium falciparum malaria with cerebral complications

B50.8 Other severe and complicated Plasmodium falciparum malaria

B50.9 Plasmodium falciparum malaria, unspecified

B51* Plasmodium vivax malaria

B52* Plasmodium malariae malaria

B53 Other parasitologically confirmed malaria

B53.0 Plasmodium ovale malaria

B53.1 Malaria due to simian plasmodia

B53.8 Other parasitologically confirmed malaria, not elsewhere classified

B54* Unspecified malaria

B55.0 Visceral leishmaniasis

B55.1 Cutaneous leishmaniasis

B55.2 Mucocutaneous leishmaniasis

B55.9 Leishmaniasis, unspecified

B56* African trypanosomiasis

B57* Chagas disease

B58 Toxoplasmosis

B58.0 Toxoplasma oculopathy

B58.1 Toxoplasma hepatitis

B58.2 Toxoplasma meningoencephalitis

B58.3 Pulmonary toxoplasmosis

B58.8 Toxoplasmosis with other organ involvement

B58.9 Toxoplasmosis, unspecified

B59* Pneumocystosis

B60.0 Babesiosis

B60.1 Acanthamoebiasis

B60.2 Naegleriasis

B60.8 Other specified protozoal diseases

B64* Unspecified protozoal disease

B65 Schistosomiasis [bilharziasis]

B65.0 Schistosomiasis due to Schistosoma haematobium [urinary schistosomiasis]

B65.1 Schistosomiasis due to Schistosoma mansoni [intestinal schistosomiasis]

B65.2 Schistosomiasis due to Schistosoma japonicum

B65.3 Cercarial dermatitis

B65.8 Other schistosomiases

B65.9 Schistosomiasis, unspecified

B66 Other fluke infections

B66.0 Opisthorchiasis

B66.1 Clonorchiasis

B66.2 Dicrocoeliasis

B66.3 Fascioliasis

B66.4 Paragonimiasis

B66.5 Fasciolopsiasis

B66.8 Other specified fluke infections

B66.9 Fluke infection, unspecified

B67* Echinococcosis

B68 Taeniasis

B68.0 Taenia solium taeniasis

B68.1 Taenia saginata taeniasis

B68.9 Taeniasis, unspecified

B69 Cysticercosis

B69.0 Cysticercosis of central nervous system

B69.1 Cysticercosis of eye

B69.8 Cysticercosis of other sites

B69.9 Cysticercosis, unspecified

B70 Diphyllobothriasis and sparganosis

B70.0 Diphyllobothriasis

B70.1 Sparganosis

B71 Other cestode infections

B71.0 Hymenolepiasis

B71.1 Dipylidiasis

B71.8 Other specified cestode infections

B71.9 Cestode infection, unspecified

B72* Dracunculiasis

B73* Onchocerciasis

B74* Filariasis

B75* Trichinellosis

B76* Hookworm diseases

B77* Ascariasis

B78* Strongyloidiasis

B79* Trichuriasis

B80* Enterobiasis

B81 Other intestinal helminthiases, not elsewhere classified

B81.0 Anisakiasis

B81.1 Intestinal capillariasis

B81.2 Trichostrongyliasis

B81.3 Intestinal angiostrongyliasis

B81.4 Mixed intestinal helminthiases

B81.8 Other specified intestinal helminthiases

B82 Unspecified intestinal parasitism

B82.0 Intestinal helminthiasis, unspecified

B82.9 Intestinal parasitism, unspecified

B83 Other helminthiases

B83.0 Visceral larva migrans

B83.1 Gnathostomiasis

B83.2 Angiostrongyliasis due to Parastrongylus cantonensis

B83.3 Syngamiasis

B83.4 Internal hirudiniasis

B83.8 Other specified helminthiases

B83.9 Helminthiasis, unspecified

B85* Pediculosis and phthiriasis

B86* Scabies

B87* Myiasis

B88 Other infestations

B88.0 Other acariasis

B88.1 Tungiasis [sandflea infestation]

B88.2 Other arthropod infestations

B88.3 External hirudiniasis

B88.8 Other specified infestations

B88.9 Infestation, unspecified

B89* Unspecified parasitic disease

B95* Streptococcus and staphylococcus as the cause of diseases classified to other chapters

B96.0 Mycoplasma pneumoniae [M. pneumoniae] as the cause of diseases classified to other chapters

B96.1 Klebsiella pneumoniae [K. pneumoniae] as the cause of diseases classified to other chapters

B96.2 Escherichia coli [E. coli] as the cause of diseases classified to other chapters

B96.3 Haemophilus influenzae [H. influenzae] as the cause of diseases classified to other chapters

B96.4 Proteus (mirabilis)(morganii) as the cause of diseases classified to other chapters

B96.5 Pseudomonas (aeruginosa) as the cause of diseases classified to other chapters

B96.6 Bacillus fragilis [B. fragilis] as the cause of diseases classified to other chapters

B96.7 Clostridium perfringens [C. perfringens] as the cause of diseases classified to other chapters

B96.8 Other specified bacterial agents as the cause of diseases classified to other chapters

B97 Viral agents as the cause of diseases classified to other chapters

B97.0 Adenovirus as the cause of diseases classified to other chapters

B97.1 Enterovirus as the cause of diseases classified to other chapters

B97.2 Coronavirus as the cause of diseases classified to other chapters

B97.3 Retrovirus as the cause of diseases classified to other chapters

B97.4 Respiratory syncytial virus as the cause of diseases classified to other chapters

B97.5 Reovirus as the cause of diseases classified to other chapters

B97.6 Parvovirus as the cause of diseases classified to other chapters

B97.7 Papillomavirus as the cause of diseases classified to other chapters

B97.8 Other viral agents as the cause of diseases classified to other chapters

B98.0 Helicobacter pylori [H.pylori] as the cause of diseases classified to other chapters

B98.1 Vibrio vulnificus as the cause of diseases classified to other chapters

B99* Other and unspecified infectious diseases

C46 Kaposi sarcoma

D73.3 Abscess of spleen

E32.1 Abscess of thymus

G00 Bacterial meningitis, not elsewhere classified

G00.0 Haemophilus meningitis

G00.1 Pneumococcal meningitis

G00.2 Streptococcal meningitis

G00.3 Staphylococcal meningitis

G00.8 Other bacterial meningitis

G00.9 Bacterial meningitis, unspecified

G01* Meningitis in bacterial diseases classified elsewhere

G02.0 Meningitis in viral diseases classified elsewhere

G02.1 Meningitis in mycoses

G02.8 Meningitis in other specified infectious and parasitic diseases classified elsewhere

G03* Meningitis due to other and unspecified causes

G04.1 Tropical spastic paraplegia

G04.2 Bacterial meningoencephalitis and meningomyelitis, not elsewhere classified

G05.0 Encephalitis, myelitis and encephalomyelitis in bacterial diseases classified elsewhere

G05.1 Encephalitis, myelitis and encephalomyelitis in viral diseases classified elsewhere

G05.2 Encephalitis, myelitis and encephalomyelitis in other infectious and parasitic diseases classified elsewhere

G06* Intracranial and intraspinal abscess and granuloma

G07* Intracranial and intraspinal abscess and granuloma in diseases classified elsewhere

H00* Hordeolum and chalazion

H01.0 Blepharitis

H05.0 Acute inflammation of orbit

H06.1 Parasitic infestation of orbit in diseases classified elsewhere

H10.0 Mucopurulent conjunctivitis

H10.5 Blepharoconjunctivitis

H13.0 Filarial infection of conjunctiva

H19.0 Scleritis and episcleritis in diseases classified elsewhere

H19.1 Herpesviral keratitis and keratoconjunctivitis

H19.2 Keratitis and keratoconjunctivitis in other infectious and parasitic diseases classified elsewhere

H22.0 Iridocyclitis in infectious and parasitic diseases classified elsewhere

H32.0 Chorioretinal inflammation in infectious and parasitic diseases classified elsewhere

H44.0 Purulent endophthalmitis

H60.0 Abscess of external ear

H60.1 Cellulitis of external ear

H60.2 Malignant otitis externa

H60.3 Other infective otitis externa

H62.0 Otitis externa in bacterial diseases classified elsewhere

H62.1 Otitis externa in viral diseases classified elsewhere

H62.2 Otitis externa in mycoses

H62.3 Otitis externa in other infectious and parasitic diseases classified elsewhere

H66.0 Acute suppurative otitis media

H67.0 Otitis media in bacterial diseases classified elsewhere

H67.1 Otitis media in viral diseases classified elsewhere

H70.0 Acute mastoiditis

H75.0 Mastoiditis in infectious and parasitic diseases classified elsewhere

I30.1 Infective pericarditis

I32.0 Pericarditis in bacterial diseases classified elsewhere

I32.1 Pericarditis in other infectious and parasitic diseases classified elsewhere

I33.0 Acute and subacute infective endocarditis

I40.0 Infective myocarditis

I41.0 Myocarditis in bacterial diseases classified elsewhere

I41.1 Myocarditis in viral diseases classified elsewhere

I41.2 Myocarditis in other infectious and parasitic diseases classified elsewhere

I43.0 Cardiomyopathy in infectious and parasitic diseases classified elsewhere

I52.0 Other heart disorders in bacterial diseases classified elsewhere

I52.1 Other heart disorders in other infectious and parasitic diseases classified elsewhere

I68.1 Cerebral arteritis in infectious and parasitic diseases classified elsewhere

J01.0 Acute maxillary sinusitis

J02 Acute pharyngitis

J02.0 Streptococcal pharyngitis

J02.8 Acute pharyngitis due to other specified organisms

J02.9 Acute pharyngitis, unspecified

J03 Acute tonsillitis

J03.0 Streptococcal tonsillitis

J03.8 Acute tonsillitis due to other specified organisms

J03.9 Acute tonsillitis, unspecified

J04* Acute laryngitis and tracheitis

J05.1 Acute epiglottitis

J09* Influenza due to certain identified influenza virus

J10* Influenza due to other identified influenza virus

J11* Influenza, virus not identified

J12* Viral pneumonia, not elsewhere classified

J13* Pneumonia due to Streptococcus pneumoniae

J14* Pneumonia due to Haemophilus influenzae

J15 Bacterial pneumonia, not elsewhere classified

J15.0 Pneumonia due to Klebsiella pneumoniae

J15.1 Pneumonia due to Pseudomonas

J15.2 Pneumonia due to staphylococcus

J15.3 Pneumonia due to streptococcus, group B

J15.4 Pneumonia due to other streptococci

J15.5 Pneumonia due to Escherichia coli

J15.6 Pneumonia due to other aerobic Gram-negative bacteria

J15.7 Pneumonia due to Mycoplasma pneumoniae

J15.8 Other bacterial pneumonia

J15.9 Bacterial pneumonia, unspecified

J16 Pneumonia due to other infectious organisms, not elsewhere classified

J16.0 Chlamydial pneumonia

J16.8 Pneumonia due to other specified infectious organisms

J17.0 Pneumonia in bacterial diseases classified elsewhere

J17.1 Pneumonia in viral diseases classified elsewhere

J17.2 Pneumonia in mycoses

J17.3 Pneumonia in parasitic diseases

J17.8 Pneumonia in other diseases classified elsewhere

J18* Pneumonia, organism unspecified

J20 Acute bronchitis

J20.0 Acute bronchitis due to Mycoplasma pneumoniae

J20.1 Acute bronchitis due to Haemophilus influenzae

J20.2 Acute bronchitis due to streptococcus

J20.3 Acute bronchitis due to coxsackievirus

J20.4 Acute bronchitis due to parainfluenza virus

J20.5 Acute bronchitis due to respiratory syncytial virus

J20.6 Acute bronchitis due to rhinovirus

J20.7 Acute bronchitis due to echovirus

J20.8 Acute bronchitis due to other specified organisms

J20.9 Acute bronchitis, unspecified

J21* Acute bronchiolitis

J21.0 Acute bronchiolitis due to respiratory syncytial virus

J21.1 Acute bronchiolitis due to human metapneumovirus

J21.8 Acute bronchiolitis due to other specified organisms

J21.9 Acute bronchiolitis, unspecified

J22* Unspecified acute lower respiratory infection

J36* Peritonsillar abscess

J39.0 Retropharyngeal and parapharyngeal abscess

J39.1 Other abscess of pharynx

J85.1 Abscess of lung with pneumonia

J85.2 Abscess of lung without pneumonia

J85.3 Abscess of mediastinum

J86* Pyothorax

K02* Dental caries

K04.4 Acute apical periodontitis of pulpal origin

K04.5 Chronic apical periodontitis

K04.6 Periapical abscess with sinus

K04.7 Periapical abscess without sinus

K05.0 Acute gingivitis

K05.2 Acute periodontitis

K05.3 Chronic periodontitis

K05.4 Periodontosis

K11.3 Abscess of salivary gland

K12.2 Cellulitis and abscess of mouth

K23.0 Tuberculous oesophagitis

K23.1 Megaoesophagus in Chagas disease

K35* Acute appendicitis

K57.0 Diverticular disease of small intestine with perforation and abscess

K57.2 Diverticular disease of large intestine with perforation and abscess

K57.4 Diverticular disease of both small and large intestine with perforation and abscess

K57.8 Diverticular disease of intestine, part unspecified, with perforation and abscess

K61* Abscess of anal and rectal regions

K63.0 Abscess of intestine

K65.0 Acute peritonitis

K67.0 Chlamydial peritonitis

K67.1 Gonococcal peritonitis

K67.2 Syphilitic peritonitis

K67.3 Tuberculous peritonitis

K67.8 Other disorders of peritoneum in infectious diseases classified elsewhere

K75.0 Abscess of liver

K77.0 Liver disorders in infectious and parasitic diseases classified elsewhere

L00* Staphylococcal scalded skin syndrome

L01* Impetigo

L02* Cutaneous abscess, furuncle and carbuncle

L03* Cellulitis

L04* Acute lymphadenitis

L05* Pilonidal cyst

L08* Other local infections of skin and subcutaneous tissue

L70.1 Acne conglobata

M00 Pyogenic arthritis

M00.0 Staphylococcal arthritis and polyarthritis

M00.1 Pneumococcal arthritis and polyarthritis

M00.2 Other streptococcal arthritis and polyarthritis

M00.8 Arthritis and polyarthritis due to other specified bacterial agents

M00.9 Pyogenic arthritis, unspecified

M01.0 Meningococcal arthritis

M01.1 Tuberculous arthritis

M01.2 Arthritis in Lyme disease

M01.3 Arthritis in other bacterial diseases classified elsewhere

M01.4 Rubella arthritis

M01.5 Arthritis in other viral diseases classified elsewhere

M01.6 Arthritis in mycoses

M01.8 Arthritis in other infectious and parasitic diseases classified elsewhere

M46.2 Osteomyelitis of vertebra

M46.3 Infection of intervertebral disc (pyogenic)

M46.5 Other infective spondylopathies

M49.0 Tuberculosis of spine

M49.1 Brucella spondylitis

M49.2 Enterobacterial spondylitis

M49.3 Spondylopathy in other infectious and parasitic diseases classified elsewhere

M60.0 Infective myositis

M63.0 Myositis in bacterial diseases classified elsewhere

M63.1 Myositis in protozoal and parasitic infections classified elsewhere

M63.2 Myositis in other infectious diseases classified elsewhere

M65.0 Abscess of tendon sheath

M65.1 Other infective (teno)synovitis

M71.0 Abscess of bursa

M71.1 Other infective bursitis

M72.6 Necrotizing fasciitis

M73.0 Gonococcal bursitis

M73.1 Syphilitic bursitis

M86* Osteomyelitis

M86.0 Acute haematogenous osteomyelitis

M86.1 Other acute osteomyelitis

M86.2 Subacute osteomyelitis

M86.3 Chronic multifocal osteomyelitis

M86.4 Chronic osteomyelitis with draining sinus

M86.5 Other chronic haematogenous osteomyelitis

M86.6 Other chronic osteomyelitis

M86.8 Other osteomyelitis

M86.9 Osteomyelitis, unspecified

N08.0 Glomerular disorders in infectious and parasitic diseases classified elsewhere

N10* Acute tubulo-interstitial nephritis

N13.6 Pyonephrosis

N15.1 Renal and perinephric abscess

N16.0 Renal tubulo-interstitial disorders in infectious and parasitic diseases classified elsewhere

N29.0 Late syphilis of kidney

N29.1 Other disorders of kidney and ureter in infectious and parasitic diseases classified elsewhere

N30.0 Acute cystitis

N34.0 Urethral abscess

N39.0 Urinary tract infection, site not specified

N41.0 Acute prostatitis

N41.2 Abscess of prostate

N45* Orchitis and epididymitis

N61* Inflammatory disorders of breast

N70.0 Acute salpingitis and oophoritis

N71.0 Acute inflammatory disease of uterus

N73.0 Acute parametritis and pelvic cellulitis

N73.3 Female acute pelvic peritonitis

N74 Female pelvic inflammatory disorders in diseases classified elsewhere

N74.0 Tuberculous infection of cervix uteri

N74.1 Female tuberculous pelvic inflammatory disease

N74.2 Female syphilitic pelvic inflammatory disease

N74.3 Female gonococcal pelvic inflammatory disease

N74.4 Female chlamydial pelvic inflammatory disease

N74.8 Female pelvic inflammatory disorders in other diseases classified elsewhere

N75.1 Abscess of Bartholin gland

N76.0 Acute vaginitis

N76.4 Abscess of vulva

N77.0 Ulceration of vulva in infectious and parasitic diseases classified elsewhere

N77.1 Vaginitis, vulvitis and vulvovaginitis in infectious and parasitic diseases classified elsewhere

O03.0 Spontaneous abortion: Incomplete, complicated by genital tract and pelvic infection

O03.5 Spontaneous abortion: Complete or unspecified, complicated by genital tract and pelvic infection

O04.0 Medical abortion: Incomplete, complicated by genital tract and pelvic infection

O04.5 Medical abortion: Complete or unspecified, complicated by genital tract and pelvic infection

O05.0 Other abortion: Incomplete, complicated by genital tract and pelvic infection

O05.5 Other abortion: Complete or unspecified, complicated by genital tract and pelvic infection

O06.0 Unspecified abortion: Incomplete, complicated by genital tract and pelvic infection

O06.5 Unspecified abortion: Complete or unspecified, complicated by genital tract and pelvic infection

O07.0 Failed medical abortion, complicated by genital tract and pelvic infection

O07.5 Other and unspecified failed attempted abortion, complicated by genital tract and pelvic infection

O08.0 Genital tract and pelvic infection following abortion and ectopic and molar pregnancy

O23.0 Infections of kidney in pregnancy

O23.1 Infections of bladder in pregnancy

O23.2 Infections of urethra in pregnancy

O23.3 Infections of other parts of urinary tract in pregnancy

O23.4 Unspecified infection of urinary tract in pregnancy

O23.5 Infections of the genital tract in pregnancy

O23.9 Other and unspecified genitourinary tract infection in pregnancy

O75.3 Other infection during labour

O85* Puerperal sepsis

O86* Other puerperal infections

O91.0 Infection of nipple associated with childbirth

O91.1 Abscess of breast associated with childbirth

O98 Maternal infectious and parasitic diseases classifiable elsewhere but complicating pregnancy, childbirth and the puerperium

O98.0 Tuberculosis complicating pregnancy, childbirth and the puerperium

O98.1 Syphilis complicating pregnancy, childbirth and the puerperium

O98.2 Gonorrhoea complicating pregnancy, childbirth and the puerperium

O98.3 Other infections with a predominantly sexual mode of transmission complicating pregnancy, childbirth and the puerperium

O98.4 Viral hepatitis complicating pregnancy, childbirth and the puerperium

O98.5 Other viral diseases complicating pregnancy, childbirth and the puerperium

O98.6 Protozoal diseases complicating pregnancy, childbirth and the puerperium

O98.7 Human immunodeficiency virus [HIV] disease complicating pregnancy, childbirth and the puerperium

O98.8 Other maternal infectious and parasitic diseases complicating pregnancy, childbirth and the puerperium

O98.9 Unspecified maternal infectious or parasitic disease complicating pregnancy, childbirth and the puerperium

P23 Congenital pneumonia

P23.0 Congenital pneumonia due to viral agent

P23.1 Congenital pneumonia due to Chlamydia

P23.2 Congenital pneumonia due to staphylococcus

P23.3 Congenital pneumonia due to streptococcus, group B

P23.4 Congenital pneumonia due to Escherichia coli

P23.5 Congenital pneumonia due to Pseudomonas

P23.6 Congenital pneumonia due to other bacterial agents

P23.8 Congenital pneumonia due to other organisms

P23.9 Congenital pneumonia, unspecified

P35.0 Congenital rubella syndrome

P35.1 Congenital cytomegalovirus infection

P35.2 Congenital herpesviral [herpes simplex] infection

P35.3 Congenital viral hepatitis

P36 Bacterial sepsis of newborn

P36.0 Sepsis of newborn due to streptococcus, group B

P36.1 Sepsis of newborn due to other and unspecified streptococci

P36.2 Sepsis of newborn due to Staphylococcus aureus

P36.3 Sepsis of newborn due to other and unspecified staphylococci

P36.4 Sepsis of newborn due to Escherichia coli

P36.5 Sepsis of newborn due to anaerobes

P36.8 Other bacterial sepsis of newborn

P36.9 Bacterial sepsis of newborn, unspecified

P37 Other congenital infectious and parasitic diseases

P37.0 Congenital tuberculosis

P37.1 Congenital toxoplasmosis

P37.2 Neonatal (disseminated) listeriosis

P37.3 Congenital falciparum malaria

P37.4 Other congenital malaria

P37.5 Neonatal candidiasis

P37.8 Other specified congenital infectious and parasitic diseases

P37.9 Congenital infectious and parasitic disease, unspecified

P38* Omphalitis of newborn with or without mild haemorrhage

P39 Other infections specific to the perinatal period

P39.0 Neonatal infective mastitis

P39.1 Neonatal conjunctivitis and dacryocystitis

P39.2 Intra-amniotic infection of fetus, not elsewhere classified

P39.3 Neonatal urinary tract infection

P39.4 Neonatal skin infection

P39.8 Other specified infections specific to the perinatal period

P39.9 Infection specific to the perinatal period, unspecified

R57.2 Septic shock

R65.0 Systemic Inflammatory Response Syndrome of infectious origin without organ failure

R65.1 Systemic Inflammatory Response Syndrome of infectious origin with organ failure

Z21 Asymptomatic human immunodeficiency virus [HIV] infection status

* indicates inclusion of underlying 4- and 5-digit codes.

**All bacterial infections**

A00* Cholera

A01* Typhoid and paratyphoid fevers

A02 Other salmonella infections

A02.0 Salmonella enteritis

A02.1 Salmonella sepsis

A02.2 Localized salmonella infections

A02.8 Other specified salmonella infections

A02.9 Salmonella infection, unspecified

A03 Shigellosis

A03.0 Shigellosis due to Shigella dysenteriae

A03.1 Shigellosis due to Shigella flexneri

A03.2 Shigellosis due to Shigella boydii

A03.3 Shigellosis due to Shigella sonnei

A03.8 Other shigellosis

A03.9 Shigellosis, unspecified

A04 Other bacterial intestinal infections

A04.0 Enteropathogenic Escherichia coli infection

A04.1 Enterotoxigenic Escherichia coli infection

A04.2 Enteroinvasive Escherichia coli infection

A04.3 Enterohaemorrhagic Escherichia coli infection

A04.4 Other intestinal Escherichia coli infections

A04.5 Campylobacter enteritis

A04.6 Enteritis due to Yersinia enterocolitica

A04.7 Enterocolitis due to Clostridium difficile

A04.8 Other specified bacterial intestinal infections

A04.9 Bacterial intestinal infection, unspecified

A05 Other bacterial foodborne intoxications, not elsewhere classified

A05.0 Foodborne staphylococcal intoxication

A05.1 Botulism

A05.2 Foodborne Clostridium perfringens [Clostridium welchii] intoxication

A05.3 Foodborne Vibrio parahaemolyticus intoxication

A05.4 Foodborne Bacillus cereus intoxication

A05.8 Other specified bacterial foodborne intoxications

A05.9 Bacterial foodborne intoxication, unspecified

A09* Other gastroenteritis and colitis of infectious and unspecified origin

A15* Respiratory tuberculosis, bacteriologically and histologically confirmed

A16* Respiratory tuberculosis, not confirmed bacteriologically or histologically

A17 Tuberculosis of nervous system

A17.0 Tuberculous meningitis

A17.1 Meningeal tuberculoma

A17.8 Other tuberculosis of nervous system

A17.9 Tuberculosis of nervous system, unspecified

A18* Tuberculosis of other organs

A19* Miliary tuberculosis

A20 Plague

A20.0 Bubonic plague

A20.1 Cellulocutaneous plague

A20.2 Pneumonic plague

A20.3 Plague meningitis

A20.7 Septicaemic plague

A20.8 Other forms of plague

A20.9 Plague, unspecified

A21 Tularaemia

A21.0 Ulceroglandular tularaemia

A21.1 Oculoglandular tularaemia

A21.2 Pulmonary tularaemia

A21.3 Gastrointestinal tularaemia

A21.7 Generalized tularaemia

A21.8 Other forms of tularaemia

A21.9 Tularaemia, unspecified

A22 Anthrax

A22.0 Cutaneous anthrax

A22.1 Pulmonary anthrax

A22.2 Gastrointestinal anthrax

A22.7 Anthrax sepsis

A22.8 Other forms of anthrax

A22.9 Anthrax, unspecified

A23 Brucellosis

A23.0 Brucellosis due to Brucella melitensis

A23.1 Brucellosis due to Brucella abortus

A23.2 Brucellosis due to Brucella suis

A23.3 Brucellosis due to Brucella canis

A23.8 Other brucellosis

A23.9 Brucellosis, unspecified

A24 Glanders and melioidosis

A24.0 Glanders

A24.1 Acute and fulminating melioidosis

A24.2 Subacute and chronic melioidosis

A24.3 Other melioidosis

A25 Rat-bite fevers

A25.0 Spirillosis

A25.1 Streptobacillosis

A25.9 Rat-bite fever, unspecified

A26 Erysipeloid

A26.0 Cutaneous erysipeloid

A26.7 Erysipelothrix sepsis

A26.8 Other forms of erysipeloid

A26.9 Erysipeloid, unspecified

A27* Leptospirosis

A28 Other zoonotic bacterial diseases, not elsewhere classified

A28.0 Pasteurellosis

A28.1 Cat-scratch disease

A28.2 Extraintestinal yersiniosis

A28.8 Other specified zoonotic bacterial diseases, not elsewhere classified

A28.9 Zoonotic bacterial disease, unspecified

A30* Leprosy [Hansen disease]

A31* Infection due to other mycobacteria

A32 Listeriosis

A32.0 Cutaneous listeriosis

A32.1 Listerial meningitis and meningoencephalitis

A32.7 Listerial sepsis

A32.8 Other forms of listeriosis

A32.9 Listeriosis, unspecified

A33* Tetanus neonatorum

A34* Obstetrical tetanus

A35* Other tetanus

A36* Diphtheria

A37* Whooping cough

A38* Scarlet fever

A39 Meningococcal infection

A39.0 Meningococcal meningitis

A39.1 Waterhouse-Friderichsen syndrome

A39.2 Acute meningococcaemia

A39.3 Chronic meningococcaemia

A39.4 Meningococcaemia, unspecified

A39.5 Meningococcal heart disease

A39.8 Other meningococcal infections

A39.9 Meningococcal infection, unspecified

A40 Streptococcal sepsis

A40.0 Sepsis due to streptococcus, group A

A40.1 Sepsis due to streptococcus, group B

A40.2 Sepsis due to streptococcus, group D

A40.3 Sepsis due to Streptococcus pneumoniae

A40.8 Other streptococcal sepsis

A40.9 Streptococcal sepsis, unspecified

A41 Other sepsis

A41.0 Sepsis due to Staphylococcus aureus

A41.1 Sepsis due to other specified staphylococcus

A41.2 Sepsis due to unspecified staphylococcus

A41.3 Sepsis due to Haemophilus influenzae

A41.4 Sepsis due to anaerobes

A41.5 Sepsis due to other Gram-negative organisms

A41.8 Other specified sepsis

A41.9 Sepsis, unspecified

A42 Actinomycosis

A42.0 Pulmonary actinomycosis

A42.1 Abdominal actinomycosis

A42.2 Cervicofacial actinomycosis

A42.7 Actinomycotic sepsis

A42.8 Other forms of actinomycosis

A42.9 Actinomycosis, unspecified

A43 Nocardiosis

A43.0 Pulmonary nocardiosis

A43.1 Cutaneous nocardiosis

A43.8 Other forms of nocardiosis

A44 Bartonellosis

A44.0 Systemic bartonellosis

A44.1 Cutaneous and mucocutaneous bartonellosis

A44.8 Other forms of bartonellosis

A44.9 Bartonellosis, unspecified

A46* Erysipelas

A48.0 Gas gangrene

A48.1 Legionnaires disease

A48.2 Nonpneumonic Legionnaires disease [Pontiac fever]

A48.3 Toxic shock syndrome

A48.4 Brazilian purpuric fever

A48.8 Other specified bacterial diseases

A49 Bacterial infection of unspecified site

A49.0 Staphylococcal infection, unspecified site

A49.1 Streptococcal infection, unspecified site

A49.2 Haemophilus influenzae infection, unspecified site

A49.3 Mycoplasma infection, unspecified site

A49.8 Other bacterial infections of unspecified site

A49.9 Bacterial infection, unspecified

A50 Congenital syphilis

A50.0 Early congenital syphilis, symptomatic

A50.1 Early congenital syphilis, latent

A50.2 Early congenital syphilis, unspecified

A50.3 Late congenital syphilitic oculopathy

A50.4 Late congenital neurosyphilis [juvenile neurosyphilis]

A50.5 Other late congenital syphilis, symptomatic

A50.6 Late congenital syphilis, latent

A50.7 Late congenital syphilis, unspecified

A50.9 Congenital syphilis, unspecified

A51 Early syphilis

A51.0 Primary genital syphilis

A51.1 Primary anal syphilis

A51.2 Primary syphilis of other sites

A51.3 Secondary syphilis of skin and mucous membranes

A51.4 Other secondary syphilis

A51.5 Early syphilis, latent

A51.9 Early syphilis, unspecified

A52 Late syphilis

A52.0 Cardiovascular syphilis

A52.1 Symptomatic neurosyphilis

A52.2 Asymptomatic neurosyphilis

A52.3 Neurosyphilis, unspecified

A52.7 Other symptomatic late syphilis

A52.8 Late syphilis, latent

A52.9 Late syphilis, unspecified

A53* Other and unspecified syphilis

A54 Gonococcal infection

A54.0 Gonococcal infection of lower genitourinary tract without periurethral or accessory gland abscess

A54.1 Gonococcal infection of lower genitourinary tract with periurethral and accessory gland abscess

A54.2 Gonococcal pelviperitonitis and other gonococcal genitourinary infections

A54.3 Gonococcal infection of eye

A54.4 Gonococcal infection of musculoskeletal system

A54.5 Gonococcal pharyngitis

A54.6 Gonococcal infection of anus and rectum

A54.8 Other gonococcal infections

A54.9 Gonococcal infection, unspecified

A55* Chlamydial lymphogranuloma (venereum)

A56.0 Chlamydial infection of lower genitourinary tract

A56.1 Chlamydial infection of pelviperitoneum and other genitourinary organs

A56.2 Chlamydial infection of genitourinary tract, unspecified

A56.3 Chlamydial infection of anus and rectum

A56.4 Chlamydial infection of pharynx

A56.8 Sexually transmitted chlamydial infection of other sites

A57* Chancroid

A58* Granuloma inguinale

A64* Unspecified sexually transmitted disease

A65* Nonvenereal syphilis

A66* Yaws

A67* Pinta [carate]

A68* Relapsing fevers

A69 Other spirochaetal infections

A69.0 Necrotizing ulcerative stomatitis

A69.1 Other Vincent infections

A69.2 Lyme disease

A69.8 Other specified spirochaetal infections

A69.9 Spirochaetal infection, unspecified

A70* Chlamydia psittaci infection

A71* Trachoma

A74.0 Chlamydial conjunctivitis

A74.8 Other chlamydial diseases

A74.9 Chlamydial infection, unspecified

A75 Typhus fever

A75.0 Epidemic louse-borne typhus fever due to Rickettsia prowazekii

A75.1 Recrudescent typhus [Brill disease]

A75.2 Typhus fever due to Rickettsia typhi

A75.3 Typhus fever due to Rickettsia tsutsugamushi

A75.9 Typhus fever, unspecified

A77 Spotted fever [tick-borne rickettsioses]

A77.0 Spotted fever due to Rickettsia rickettsii

A77.1 Spotted fever due to Rickettsia conorii

A77.2 Spotted fever due to Rickettsia sibirica

A77.3 Spotted fever due to Rickettsia australis

A77.8 Other spotted fevers

A77.9 Spotted fever, unspecified

A78* Q fever

A79 Other rickettsioses

A79.0 Trench fever

A79.1 Rickettsialpox due to Rickettsia akari

A79.8 Other specified rickettsioses

A79.9 Rickettsiosis, unspecified

B47.1 Actinomycetoma

B95* Streptococcus and staphylococcus as the cause of diseases classified to other chapters

B96.0 Mycoplasma pneumoniae [M. pneumoniae] as the cause of diseases classified to other chapters

B96.1 Klebsiella pneumoniae [K. pneumoniae] as the cause of diseases classified to other chapters

B96.2 Escherichia coli [E. coli] as the cause of diseases classified to other chapters

B96.3 Haemophilus influenzae [H. influenzae] as the cause of diseases classified to other chapters

B96.4 Proteus (mirabilis)(morganii) as the cause of diseases classified to other chapters

B96.5 Pseudomonas (aeruginosa) as the cause of diseases classified to other chapters

B96.6 Bacillus fragilis [B. fragilis] as the cause of diseases classified to other chapters

B96.7 Clostridium perfringens [C. perfringens] as the cause of diseases classified to other chapters

B96.8 Other specified bacterial agents as the cause of diseases classified to other chapters

B98.0 Helicobacter pylori [H.pylori] as the cause of diseases classified to other chapters

B98.1 Vibrio vulnificus as the cause of diseases classified to other chapters

D73.3 Abscess of spleen

E32.1 Abscess of thymus

G00 Bacterial meningitis, not elsewhere classified

G00.0 Haemophilus meningitis

G00.1 Pneumococcal meningitis

G00.2 Streptococcal meningitis

G00.3 Staphylococcal meningitis

G00.8 Other bacterial meningitis

G00.9 Bacterial meningitis, unspecified

G01* Meningitis in bacterial diseases classified elsewhere

G04.2 Bacterial meningoencephalitis and meningomyelitis, not elsewhere classified

G05.0 Encephalitis, myelitis and encephalomyelitis in bacterial diseases classified elsewhere

G06* Intracranial and intraspinal abscess and granuloma

H00* Hordeolum and chalazion

H01.0 Blepharitis

H05.0 Acute inflammation of orbit

H10.0 Mucopurulent conjunctivitis

H10.5 Blepharoconjunctivitis

H44.0 Purulent endophthalmitis

H60.0 Abscess of external ear

H60.1 Cellulitis of external ear

H60.2 Malignant otitis externa

H60.3 Other infective otitis externa

H62.0 Otitis externa in bacterial diseases classified elsewhere

H66.0 Acute suppurative otitis media

H67.0 Otitis media in bacterial diseases classified elsewhere

H70.0 Acute mastoiditis

I32.0 Pericarditis in bacterial diseases classified elsewhere

I33.0 Acute and subacute infective endocarditis

I41.0 Myocarditis in bacterial diseases classified elsewhere

I52.0 Other heart disorders in bacterial diseases classified elsewhere

J01.0 Acute maxillary sinusitis

J02 Acute pharyngitis

J02.0 Streptococcal pharyngitis

J02.8 Acute pharyngitis due to other specified organisms

J02.9 Acute pharyngitis, unspecified

J03 Acute tonsillitis

J03.0 Streptococcal tonsillitis

J03.8 Acute tonsillitis due to other specified organisms

J03.9 Acute tonsillitis, unspecified

J04* Acute laryngitis and tracheitis

J05.1 Acute epiglottitis

J13* Pneumonia due to Streptococcus pneumoniae

J14* Pneumonia due to Haemophilus influenzae

J15 Bacterial pneumonia, not elsewhere classified

J15.0 Pneumonia due to Klebsiella pneumoniae

J15.1 Pneumonia due to Pseudomonas

J15.2 Pneumonia due to staphylococcus

J15.3 Pneumonia due to streptococcus, group B

J15.4 Pneumonia due to other streptococci

J15.5 Pneumonia due to Escherichia coli

J15.6 Pneumonia due to other aerobic Gram-negative bacteria

J15.7 Pneumonia due to Mycoplasma pneumoniae

J15.8 Other bacterial pneumonia

J15.9 Bacterial pneumonia, unspecified

J16 Pneumonia due to other infectious organisms, not elsewhere classified

J16.0 Chlamydial pneumonia

J16.8 Pneumonia due to other specified infectious organisms

J17.0 Pneumonia in bacterial diseases classified elsewhere

J17.8 Pneumonia in other diseases classified elsewhere

J18* Pneumonia, organism unspecified

J20.0 Acute bronchitis due to Mycoplasma pneumoniae

J20.1 Acute bronchitis due to Haemophilus influenzae

J20.2 Acute bronchitis due to streptococcus

J22* Unspecified acute lower respiratory infection

J36* Peritonsillar abscess

J39.0 Retropharyngeal and parapharyngeal abscess

J39.1 Other abscess of pharynx

J85.1 Abscess of lung with pneumonia

J85.2 Abscess of lung without pneumonia

J85.3 Abscess of mediastinum

J86* Pyothorax

K02* Dental caries

K04.4 Acute apical periodontitis of pulpal origin

K04.5 Chronic apical periodontitis

K04.6 Periapical abscess with sinus

K04.7 Periapical abscess without sinus

K05.0 Acute gingivitis

K05.2 Acute periodontitis

K05.3 Chronic periodontitis

K05.4 Periodontosis

K11.3 Abscess of salivary gland

K12.2 Cellulitis and abscess of mouth

K23.0 Tuberculous oesophagitis

K35* Acute appendicitis

K57.0 Diverticular disease of small intestine with perforation and abscess

K57.2 Diverticular disease of large intestine with perforation and abscess

K57.4 Diverticular disease of both small and large intestine with perforation and abscess

K57.8 Diverticular disease of intestine, part unspecified, with perforation and abscess

K61* Abscess of anal and rectal regions

K63.0 Abscess of intestine

K65.0 Acute peritonitis

K67.0 Chlamydial peritonitis

K67.1 Gonococcal peritonitis

K67.2 Syphilitic peritonitis

K67.3 Tuberculous peritonitis

K75.0 Abscess of liver

L00* Staphylococcal scalded skin syndrome

L01* Impetigo

L02* Cutaneous abscess, furuncle and carbuncle

L03* Cellulitis

L04* Acute lymphadenitis

L05* Pilonidal cyst

L08* Other local infections of skin and subcutaneous tissue

L70.1 Acne conglobata

M00 Pyogenic arthritis

M00.0 Staphylococcal arthritis and polyarthritis

M00.1 Pneumococcal arthritis and polyarthritis

M00.2 Other streptococcal arthritis and polyarthritis

M00.8 Arthritis and polyarthritis due to other specified bacterial agents

M00.9 Pyogenic arthritis, unspecified

M01.0 Meningococcal arthritis

M01.1 Tuberculous arthritis

M01.2 Arthritis in Lyme disease

M01.3 Arthritis in other bacterial diseases classified elsewhere

M46.2 Osteomyelitis of vertebra

M46.3 Infection of intervertebral disc (pyogenic)

M46.5 Other infective spondylopathies

M49.0 Tuberculosis of spine

M49.1 Brucella spondylitis

M49.2 Enterobacterial spondylitis

M63.0 Myositis in bacterial diseases classified elsewhere

M65.0 Abscess of tendon sheath

M65.1 Other infective (teno)synovitis

M71.0 Abscess of bursa

M71.1 Other infective bursitis

M72.6 Necrotizing fasciitis

M73.0 Gonococcal bursitis

M73.1 Syphilitic bursitis

M86* Osteomyelitis

M86.0 Acute haematogenous osteomyelitis

M86.1 Other acute osteomyelitis

M86.2 Subacute osteomyelitis

M86.3 Chronic multifocal osteomyelitis

M86.4 Chronic osteomyelitis with draining sinus

M86.5 Other chronic haematogenous osteomyelitis

M86.6 Other chronic osteomyelitis

M86.8 Other osteomyelitis

M86.9 Osteomyelitis, unspecified

N10* Acute tubulo-interstitial nephritis

N13.6 Pyonephrosis

N15.1 Renal and perinephric abscess

N29.0 Late syphilis of kidney

N30.0 Acute cystitis

N34.0 Urethral abscess

N39.0 Urinary tract infection, site not specified

N41.0 Acute prostatitis

N41.2 Abscess of prostate

N45* Orchitis and epididymitis

N61* Inflammatory disorders of breast

N70.0 Acute salpingitis and oophoritis

N71.0 Acute inflammatory disease of uterus

N73.0 Acute parametritis and pelvic cellulitis

N73.3 Female acute pelvic peritonitis

N74.0 Tuberculous infection of cervix uteri

N74.1 Female tuberculous pelvic inflammatory disease

N74.2 Female syphilitic pelvic inflammatory disease

N74.3 Female gonococcal pelvic inflammatory disease

N74.4 Female chlamydial pelvic inflammatory disease

N75.1 Abscess of Bartholin gland

N76.0 Acute vaginitis

N76.4 Abscess of vulva

O03.0 Spontaneous abortion: Incomplete, complicated by genital tract and pelvic infection

O03.5 Spontaneous abortion: Complete or unspecified, complicated by genital tract and pelvic infection

O04.0 Medical abortion: Incomplete, complicated by genital tract and pelvic infection

O04.5 Medical abortion: Complete or unspecified, complicated by genital tract and pelvic infection

O05.0 Other abortion: Incomplete, complicated by genital tract and pelvic infection

O05.5 Other abortion: Complete or unspecified, complicated by genital tract and pelvic infection

O06.0 Unspecified abortion: Incomplete, complicated by genital tract and pelvic infection

O06.5 Unspecified abortion: Complete or unspecified, complicated by genital tract and pelvic infection

O07.0 Failed medical abortion, complicated by genital tract and pelvic infection

O07.5 Other and unspecified failed attempted abortion, complicated by genital tract and pelvic infection

O08.0 Genital tract and pelvic infection following abortion and ectopic and molar pregnancy

O23.0 Infections of kidney in pregnancy

O23.1 Infections of bladder in pregnancy

O23.2 Infections of urethra in pregnancy

O23.3 Infections of other parts of urinary tract in pregnancy

O23.4 Unspecified infection of urinary tract in pregnancy

O23.5 Infections of the genital tract in pregnancy

O23.9 Other and unspecified genitourinary tract infection in pregnancy

O75.3 Other infection during labour

O85* Puerperal sepsis

O86* Other puerperal infections

O91.0 Infection of nipple associated with childbirth

O91.1 Abscess of breast associated with childbirth

O98.0 Tuberculosis complicating pregnancy, childbirth and the puerperium

O98.1 Syphilis complicating pregnancy, childbirth and the puerperium

O98.2 Gonorrhoea complicating pregnancy, childbirth and the puerperium

O98.3 Other infections with a predominantly sexual mode of transmission complicating pregnancy, childbirth and the puerperium

O98.8 Other maternal infectious and parasitic diseases complicating pregnancy, childbirth and the puerperium

O98.9 Unspecified maternal infectious or parasitic disease complicating pregnancy, childbirth and the puerperium

P23 Congenital pneumonia

P23.1 Congenital pneumonia due to Chlamydia

P23.2 Congenital pneumonia due to staphylococcus

P23.3 Congenital pneumonia due to streptococcus, group B

P23.4 Congenital pneumonia due to Escherichia coli

P23.5 Congenital pneumonia due to Pseudomonas

P23.6 Congenital pneumonia due to other bacterial agents

P23.9 Congenital pneumonia, unspecified

P36 Bacterial sepsis of newborn

P36.0 Sepsis of newborn due to streptococcus, group B

P36.1 Sepsis of newborn due to other and unspecified streptococci

P36.2 Sepsis of newborn due to Staphylococcus aureus

P36.3 Sepsis of newborn due to other and unspecified staphylococci

P36.4 Sepsis of newborn due to Escherichia coli

P36.5 Sepsis of newborn due to anaerobes

P36.8 Other bacterial sepsis of newborn

P36.9 Bacterial sepsis of newborn, unspecified

P37.0 Congenital tuberculosis

P37.2 Neonatal (disseminated) listeriosis

P38* Omphalitis of newborn with or without mild haemorrhage

P39.0 Neonatal infective mastitis

P39.1 Neonatal conjunctivitis and dacryocystitis

P39.2 Intra-amniotic infection of fetus, not elsewhere classified

P39.3 Neonatal urinary tract infection

P39.4 Neonatal skin infection

**All viral infections**

A08.0 Rotaviral enteritis

A08.1 Acute gastroenteropathy due to Norwalk agent

A08.2 Adenoviral enteritis

A08.3 Other viral enteritis

A08.4 Viral intestinal infection, unspecified

A60* Anogenital herpesviral [herpes simplex] infection

A63.0 Anogenital (venereal) warts

A80* Acute poliomyelitis

A81.1 Subacute sclerosing panencephalitis

A81.2 Progressive multifocal leukoencephalopathy

A83* Mosquito-borne viral encephalitis

A84* Tick-borne viral encephalitis

A85* Other viral encephalitis, not elsewhere classified

A86* Unspecified viral encephalitis

A87* Viral meningitis

A88* Other viral infections of central nervous system, not elsewhere classified

A89* Unspecified viral infection of central nervous system

A90* Dengue fever [classical dengue]

A91* Dengue haemorrhagic fever

A92 Other mosquito-borne viral fevers

A92.0 Chikungunya virus disease

A92.1 O'nyong-nyong fever

A92.2 Venezuelan equine fever

A92.3 West Nile virus infection

A92.4 Rift Valley fever

A92.8 Other specified mosquito-borne viral fevers

A92.9 Mosquito-borne viral fever, unspecified

A93 Other arthropod-borne viral fevers, not elsewhere classified

A93.0 Oropouche virus disease

A93.1 Sandfly fever

A93.2 Colorado tick fever

A93.8 Other specified arthropod-borne viral fevers

A94* Unspecified arthropod-borne viral fever

A95* Yellow fever

A96* Arenaviral haemorrhagic fever

A97* Dengue

A98 Other viral haemorrhagic fevers, not elsewhere classified

A98.0 Crimean-Congo haemorrhagic fever

A98.1 Omsk haemorrhagic fever

A98.2 Kyasanur Forest disease

A98.3 Marburg virus disease

A98.4 Ebola virus disease

A98.5 Haemorrhagic fever with renal syndrome

A98.8 Other specified viral haemorrhagic fevers

A99* Unspecified viral haemorrhagic fever

B00 Herpesviral [herpes simplex] infections

B00.0 Eczema herpeticum

B00.1 Herpesviral vesicular dermatitis

B00.2 Herpesviral gingivostomatitis and pharyngotonsillitis

B00.3 Herpesviral meningitis

B00.4 Herpesviral encephalitis

B00.5 Herpesviral ocular disease

B00.7 Disseminated herpesviral disease

B00.8 Other forms of herpesviral infection

B00.9 Herpesviral infection, unspecified

B01 Varicella [chickenpox]

B01.0 Varicella meningitis

B01.1 Varicella encephalitis

B01.2 Varicella pneumonia

B01.8 Varicella with other complications

B01.9 Varicella without complication

B02 Zoster [herpes zoster]

B02.0 Zoster encephalitis

B02.1 Zoster meningitis

B02.2 Zoster with other nervous system involvement

B02.3 Zoster ocular disease

B02.7 Disseminated zoster

B02.8 Zoster with other complications

B02.9 Zoster without complication

B04* Monkeypox

B05 Measles

B05.0 Measles complicated by encephalitis

B05.1 Measles complicated by meningitis

B05.2 Measles complicated by pneumonia

B05.3 Measles complicated by otitis media

B05.4 Measles with intestinal complications

B05.8 Measles with other complications

B05.9 Measles without complication

B06 Rubella [German measles]

B06.0 Rubella with neurological complications

B06.8 Rubella with other complications

B06.9 Rubella without complication

B07* Viral warts

B08.0 Other orthopoxvirus infections

B08.1 Molluscum contagiosum

B08.2 Exanthema subitum [sixth disease]

B08.3 Erythema infectiosum [fifth disease]

B08.4 Enteroviral vesicular stomatitis with exanthem

B08.5 Enteroviral vesicular pharyngitis

B08.8 Other specified viral infections characterized by skin and mucous membrane lesions

B09* Unspecified viral infection characterized by skin and mucous membrane lesions

B15 Acute hepatitis A

B15.0 Hepatitis A with hepatic coma

B15.9 Hepatitis A without hepatic coma

B16 Acute hepatitis B

B16.0 Acute hepatitis B with delta-agent (coinfection) with hepatic coma

B16.1 Acute hepatitis B with delta-agent (coinfection) without hepatic coma

B16.2 Acute hepatitis B without delta-agent with hepatic coma

B16.9 Acute hepatitis B without delta-agent and without hepatic coma

B17* Other acute viral hepatitis

B18* Chronic viral hepatitis

B19 Unspecified viral hepatitis

B19.0 Unspecified viral hepatitis with hepatic coma

B19.9 Unspecified viral hepatitis without hepatic coma

B20* Human immunodeficiency virus [HIV] disease resulting in infectious and parasitic diseases

B21* Human immunodeficiency virus [HIV] disease resulting in malignant neoplasms

B21.0 HIV disease resulting in Kaposi sarcoma

B22 Human immunodeficiency virus [HIV] disease resulting in other specified diseases

B22.0 HIV disease resulting in encephalopathy

B22.1 HIV disease resulting in lymphoid interstitial pneumonitis

B22.2 HIV disease resulting in wasting syndrome

B22.7 HIV disease resulting in multiple diseases classified elsewhere

B23* Human immunodeficiency virus [HIV] disease resulting in other conditions

B24* Unspecified human immunodeficiency virus [HIV] disease

B25* Cytomegaloviral disease

B26 Mumps

B26.0 Mumps orchitis

B26.1 Mumps meningitis

B26.2 Mumps encephalitis

B26.3 Mumps pancreatitis

B26.8 Mumps with other complications

B26.9 Mumps without complication

B27 Infectious mononucleosis

B27.0 Gammaherpesviral mononucleosis

B27.1 Cytomegaloviral mononucleosis

B27.9 Infectious mononucleosis, unspecified

B30* Viral conjunctivitis

B33 Other viral diseases, not elsewhere classified

B33.0 Epidemic myalgia

B33.1 Ross River disease

B33.2 Viral carditis

B33.3 Retrovirus infections, not elsewhere classified

B33.4 Hantavirus (cardio-)pulmonary syndrome [HPS] [HCPS]

B33.8 Other specified viral diseases

B34 Viral infection of unspecified site

B34.0 Adenovirus infection, unspecified site

B34.1 Enterovirus infection, unspecified site

B34.2 Coronavirus infection, unspecified site

B34.3 Parvovirus infection, unspecified site

B34.4 Papovavirus infection, unspecified site

B34.8 Other viral infections of unspecified site

B34.9 Viral infection, unspecified

B97 Viral agents as the cause of diseases classified to other chapters

B97.0 Adenovirus as the cause of diseases classified to other chapters

B97.1 Enterovirus as the cause of diseases classified to other chapters

B97.2 Coronavirus as the cause of diseases classified to other chapters

B97.3 Retrovirus as the cause of diseases classified to other chapters

B97.4 Respiratory syncytial virus as the cause of diseases classified to other chapters

B97.5 Reovirus as the cause of diseases classified to other chapters

B97.6 Parvovirus as the cause of diseases classified to other chapters

B97.7 Papillomavirus as the cause of diseases classified to other chapters

B97.8 Other viral agents as the cause of diseases classified to other chapters

C46 Kaposi sarcoma

G02.0 Meningitis in viral diseases classified elsewhere

G04.1 Tropical spastic paraplegia

G05.1 Encephalitis, myelitis and encephalomyelitis in viral diseases classified elsewhere

H19.1 Herpesviral keratitis and keratoconjunctivitis

H62.1 Otitis externa in viral diseases classified elsewhere

H67.1 Otitis media in viral diseases classified elsewhere

I41.1 Myocarditis in viral diseases classified elsewhere

J09* Influenza due to certain identified influenza virus

J10* Influenza due to other identified influenza virus

J11* Influenza, virus not identified

J12* Viral pneumonia, not elsewhere classified

J17.1 Pneumonia in viral diseases classified elsewhere

J20.3 Acute bronchitis due to coxsackievirus

J20.4 Acute bronchitis due to parainfluenza virus

J20.5 Acute bronchitis due to respiratory syncytial virus

J20.6 Acute bronchitis due to rhinovirus

J20.7 Acute bronchitis due to echovirus

J21* Acute bronchiolitis

J21.0 Acute bronchiolitis due to respiratory syncytial virus

J21.1 Acute bronchiolitis due to human metapneumovirus

J21.8 Acute bronchiolitis due to other specified organisms

J21.9 Acute bronchiolitis, unspecified

M01.4 Rubella arthritis

M01.5 Arthritis in other viral diseases classified elsewhere

O98.4 Viral hepatitis complicating pregnancy, childbirth and the puerperium

O98.5 Other viral diseases complicating pregnancy, childbirth and the puerperium

O98.7 Human immunodeficiency virus [HIV] disease complicating pregnancy, childbirth and the puerperium

P23.0 Congenital pneumonia due to viral agent

P35.0 Congenital rubella syndrome

P35.1 Congenital cytomegalovirus infection

P35.2 Congenital herpesviral [herpes simplex] infection

P35.3 Congenital viral hepatitis

Z21 Asymptomatic human immunodeficiency virus [HIV] infection status

**Fungal infections (mycoses)**

B35* Dermatophytosis

B36* Other superficial mycoses

B37 Candidiasis

B37.0 Candidal stomatitis

B37.1 Pulmonary candidiasis

B37.2 Candidiasis of skin and nail

B37.3 Candidiasis of vulva and vagina

B37.4 Candidiasis of other urogenital sites

B37.5 Candidal meningitis

B37.6 Candidal endocarditis

B37.7 Candidal sepsis

B37.8 Candidiasis of other sites

B37.9 Candidiasis, unspecified

B38 Coccidioidomycosis

B38.0 Acute pulmonary coccidioidomycosis

B38.1 Chronic pulmonary coccidioidomycosis

B38.2 Pulmonary coccidioidomycosis, unspecified

B38.3 Cutaneous coccidioidomycosis

B38.4 Coccidioidomycosis meningitis

B38.7 Disseminated coccidioidomycosis

B38.8 Other forms of coccidioidomycosis

B38.9 Coccidioidomycosis, unspecified

B39 Histoplasmosis

B39.0 Acute pulmonary histoplasmosis capsulati

B39.1 Chronic pulmonary histoplasmosis capsulati

B39.2 Pulmonary histoplasmosis capsulati, unspecified

B39.3 Disseminated histoplasmosis capsulati

B39.4 Histoplasmosis capsulati, unspecified

B39.5 Histoplasmosis duboisii

B39.9 Histoplasmosis, unspecified

B40 Blastomycosis

B40.0 Acute pulmonary blastomycosis

B40.1 Chronic pulmonary blastomycosis

B40.2 Pulmonary blastomycosis, unspecified

B40.3 Cutaneous blastomycosis

B40.7 Disseminated blastomycosis

B40.8 Other forms of blastomycosis

B40.9 Blastomycosis, unspecified

B41 Paracoccidioidomycosis

B41.0 Pulmonary paracoccidioidomycosis

B41.7 Disseminated paracoccidioidomycosis

B41.8 Other forms of paracoccidioidomycosis

B41.9 Paracoccidioidomycosis, unspecified

B42 Sporotrichosis

B42.0 Pulmonary sporotrichosis

B42.1 Lymphocutaneous sporotrichosis

B42.7 Disseminated sporotrichosis

B42.8 Other forms of sporotrichosis

B42.9 Sporotrichosis, unspecified

B43 Chromomycosis and phaeomycotic abscess

B43.0 Cutaneous chromomycosis

B43.1 Phaeomycotic brain abscess

B43.2 Subcutaneous phaeomycotic abscess and cyst

B43.8 Other forms of chromomycosis

B43.9 Chromomycosis, unspecified

B44* Aspergillosis

B45 Cryptococcosis

B45.0 Pulmonary cryptococcosis

B45.1 Cerebral cryptococcosis

B45.2 Cutaneous cryptococcosis

B45.3 Osseous cryptococcosis

B45.7 Disseminated cryptococcosis

B45.8 Other forms of cryptococcosis

B45.9 Cryptococcosis, unspecified

B46 Zygomycosis

B46.0 Pulmonary mucormycosis

B46.1 Rhinocerebral mucormycosis

B46.2 Gastrointestinal mucormycosis

B46.3 Cutaneous mucormycosis

B46.4 Disseminated mucormycosis

B46.5 Mucormycosis, unspecified

B46.8 Other zygomycoses

B46.9 Zygomycosis, unspecified

B47.0 Eumycetoma

B48 Other mycoses, not elsewhere classified

B48.0 Lobomycosis

B48.1 Rhinosporidiosis

B48.2 Allescheriasis

B48.3 Geotrichosis

B48.4 Penicillosis

B48.7 Opportunistic mycoses

B48.8 Other specified mycoses

B49* Unspecified mycosis

B59* Pneumocystosis

G02.1 Meningitis in mycoses

H62.2 Otitis externa in mycoses

J17.2 Pneumonia in mycoses

M01.6 Arthritis in mycoses

P37.5 Neonatal candidiasis

**Parasitic infections**

A06 Amoebiasis

A06.0 Acute amoebic dysentery

A06.1 Chronic intestinal amoebiasis

A06.2 Amoebic nondysenteric colitis

A06.3 Amoeboma of intestine

A06.4 Amoebic liver abscess

A06.5 Amoebic lung abscess

A06.6 Amoebic brain abscess

A06.7 Cutaneous amoebiasis

A06.8 Amoebic infection of other sites

A06.9 Amoebiasis, unspecified

A07* Other protozoal intestinal diseases

A59* Trichomoniasis

B50* Plasmodium falciparum malaria

B50.0 Plasmodium falciparum malaria with cerebral complications

B50.8 Other severe and complicated Plasmodium falciparum malaria

B50.9 Plasmodium falciparum malaria, unspecified

B51* Plasmodium vivax malaria

B52* Plasmodium malariae malaria

B53 Other parasitologically confirmed malaria

B53.0 Plasmodium ovale malaria

B53.1 Malaria due to simian plasmodia

B53.8 Other parasitologically confirmed malaria, not elsewhere classified

B54* Unspecified malaria

B55.0 Visceral leishmaniasis

B55.1 Cutaneous leishmaniasis

B55.2 Mucocutaneous leishmaniasis

B55.9 Leishmaniasis, unspecified

B56* African trypanosomiasis

B57* Chagas disease

B58 Toxoplasmosis

B58.0 Toxoplasma oculopathy

B58.1 Toxoplasma hepatitis

B58.2 Toxoplasma meningoencephalitis

B58.3 Pulmonary toxoplasmosis

B58.8 Toxoplasmosis with other organ involvement

B58.9 Toxoplasmosis, unspecified

B60.0 Babesiosis

B60.1 Acanthamoebiasis

B60.2 Naegleriasis

B60.8 Other specified protozoal diseases

B64* Unspecified protozoal disease

B65 Schistosomiasis [bilharziasis]

B65.0 Schistosomiasis due to Schistosoma haematobium [urinary schistosomiasis]

B65.1 Schistosomiasis due to Schistosoma mansoni [intestinal schistosomiasis]

B65.2 Schistosomiasis due to Schistosoma japonicum

B65.3 Cercarial dermatitis

B65.8 Other schistosomiases

B65.9 Schistosomiasis, unspecified

B66 Other fluke infections

B66.0 Opisthorchiasis

B66.1 Clonorchiasis

B66.2 Dicrocoeliasis

B66.3 Fascioliasis

B66.4 Paragonimiasis

B66.5 Fasciolopsiasis

B66.8 Other specified fluke infections

B66.9 Fluke infection, unspecified

B67* Echinococcosis

B68 Taeniasis

B68.0 Taenia solium taeniasis

B68.1 Taenia saginata taeniasis

B68.9 Taeniasis, unspecified

B69 Cysticercosis

B69.0 Cysticercosis of central nervous system

B69.1 Cysticercosis of eye

B69.8 Cysticercosis of other sites

B69.9 Cysticercosis, unspecified

B70 Diphyllobothriasis and sparganosis

B70.0 Diphyllobothriasis

B70.1 Sparganosis

B71 Other cestode infections

B71.0 Hymenolepiasis

B71.1 Dipylidiasis

B71.8 Other specified cestode infections

B71.9 Cestode infection, unspecified

B72* Dracunculiasis

B73* Onchocerciasis

B74* Filariasis

B75* Trichinellosis

B76* Hookworm diseases

B77* Ascariasis

B78* Strongyloidiasis

B79* Trichuriasis

B80* Enterobiasis

B81 Other intestinal helminthiases, not elsewhere classified

B81.0 Anisakiasis

B81.1 Intestinal capillariasis

B81.2 Trichostrongyliasis

B81.3 Intestinal angiostrongyliasis

B81.4 Mixed intestinal helminthiases

B81.8 Other specified intestinal helminthiases

B82 Unspecified intestinal parasitism

B82.0 Intestinal helminthiasis, unspecified

B82.9 Intestinal parasitism, unspecified

B83 Other helminthiases

B83.0 Visceral larva migrans

B83.1 Gnathostomiasis

B83.2 Angiostrongyliasis due to Parastrongylus cantonensis

B83.3 Syngamiasis

B83.4 Internal hirudiniasis

B83.8 Other specified helminthiases

B83.9 Helminthiasis, unspecified

B85* Pediculosis and phthiriasis

B86* Scabies

B87* Myiasis

B88 Other infestations

B88.0 Other acariasis

B88.1 Tungiasis [sandflea infestation]

B88.2 Other arthropod infestations

B88.3 External hirudiniasis

B88.8 Other specified infestations

B88.9 Infestation, unspecified

B89* Unspecified parasitic disease

H06.1 Parasitic infestation of orbit in diseases classified elsewhere

H13.0 Filarial infection of conjunctiva

J17.3 Pneumonia in parasitic diseases

K23.1 Megaoesophagus in Chagas disease

M63.1 Myositis in protozoal and parasitic infections classified elsewhere

O98.6 Protozoal diseases complicating pregnancy, childbirth and the puerperium

P37.1 Congenital toxoplasmosis

P37.3 Congenital falciparum malaria

P37.4 Other congenital malaria

**Reference**

1. Littlejohns, T. J.; Holliday, J.; Gibson, L. M.; Garratt, S.; Oesingmann, N.; Alfaro-Almagro, F., et al., The UK Biobank imaging enhancement of 100,000 participants: rationale, data collection, management and future directions. *Nat. Commun.* **2020,** *11* (1), 2624.

2. Petersen, S. E.; Matthews, P. M.; Francis, J. M.; Robson, M. D.; Zemrak, F.; Boubertakh, R., et al., UK Biobank's cardiovascular magnetic resonance protocol. *J. Cardiovasc. Magn. Reson.* **2016,** *18* (1), 8.

3. Bai, W.; Sinclair, M.; Tarroni, G.; Oktay, O.; Rajchl, M.; Vaillant, G., et al., Automated cardiovascular magnetic resonance image analysis with fully convolutional networks. *J. Cardiovasc. Magn. Reson.* **2018,** *20* (1), 65.

4. Schulz-Menger, J.; Bluemke, D. A.; Bremerich, J.; Flamm, S. D.; Fogel, M. A.; Friedrich, M. G., et al., Standardized image interpretation and post-processing in cardiovascular magnetic resonance-2020 update: Society for Cardiovascular Magnetic Resonance (SCMR): Board of Trustees Task Force on Standardized Post-Processing. *J. Cardiovasc. Magn. Reson.* **2020,** *22* (1), 19.

5. Kleiber, M., Body size and metabolic rate. *Physiol. Rev.* **1947,** *27* (4), 511-541.

6. Bailey, B.; Briars, G., Estimating the surface area of the human body. *Stat. Med.* **1996,** *15* (13), 1325-1332.

7. Wain, L. V.; Shrine, N.; Miller, S.; Jackson, V. E.; Ntalla, I.; Artigas, M. S., et al., Novel insights into the genetics of smoking behaviour, lung function, and chronic obstructive pulmonary disease (UK BiLEVE): a genetic association study in UK Biobank. *The Lancet Respiratory Medicine* **2015,** *3* (10), 769-781.

8. Pankow, J. S.; Tang, W.; Pankratz, N.; Guan, W.; Weng, L.-C.; Cushman, M., et al., Identification of genetic variants linking protein C and lipoprotein metabolism: the ARIC study (Atherosclerosis Risk in Communities). *Arterioscler. Thromb. Vasc. Biol.* **2017,** *37* (3), 589-597.

9. Taliun, D.; Harris, D. N.; Kessler, M. D.; Carlson, J.; Szpiech, Z. A.; Torres, R., et al., Sequencing of 53,831 diverse genomes from the NHLBI TOPMed Program. *Nature* **2021,** *590* (7845), 290-299.

10. Byun, J.; Han, Y.; Gorlov, I. P.; Busam, J. A.; Seldin, M. F.; Amos, C. I., Ancestry inference using principal component analysis and spatial analysis: a distance-based analysis to account for population substructure. *BMC Genomics* **2017,** *18*, 1-12.
